# Enantioselective fluorination of α-branched aldehydes and subsequent conversion to α-hydroxyacetals *via* stereospecific C–F bond cleavage†
†Electronic supplementary information (ESI) available: Experimental details including characterization date, copies of ^1^H, ^13^C, ^19^F NMR and HPLC traces. See DOI: 10.1039/c5sc03486h


**DOI:** 10.1039/c5sc03486h

**Published:** 2015-11-16

**Authors:** Kazutaka Shibatomi, Kazumasa Kitahara, Takuya Okimi, Yoshiyuki Abe, Seiji Iwasa

**Affiliations:** a Department of Environmental and Life Sciences , Toyohashi University of Technology , 1-1 Hibarigaoka, Tempaku-cho , Toyohashi 441-8580 , Japan . Email: shiba@ens.tut.ac.jp

## Abstract

The highly enantioselective fluorination of α-branched aldehydes was achieved using newly developed chiral primary amine catalyst **1**. Furthermore, the C–F bond cleavage of the resulting α-fluoroaldehydes proceeded smoothly under alcoholic alkaline conditions to yield the corresponding α-hydroxyacetals in a stereospecific manner. Accordingly, the one-pot conversion of α-branched aldehydes into α-hydroxyacetals was achieved for the first time in high enantioselectivity.

## Abstract 2

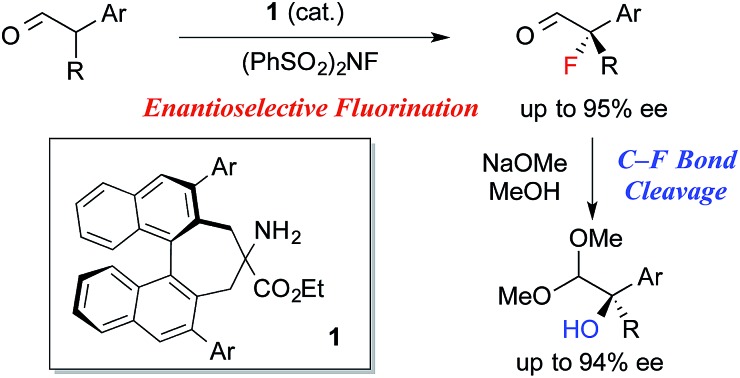
The highly enantioselective fluorination of α-branched aldehydes was achieved using newly developed chiral primary amine catalyst.

## 

Enantioselective construction of fluorinated chiral stereogenic centers is synthetically important, because the resulting fluorides are expected to be useful intermediates for fluorinated drugs and agricultural agents.^1^ Despite the extraordinary interest in practical synthetic methodologies towards chiral tertiary fluorides, until very recently, catalytic enantioselective methods capable of introducing fluorine atoms onto a tertiary carbon center have been primarily limited to the fluorination of active methine compounds.^2^–^4^ The chiral secondary amine-catalyzed electrophilic fluorination of aldehydes is a highly useful method for the construction of fluorinated stereogenic centers.^5^ Although this method yields α-fluoroaldehydes with high enantioselectivity when α-monosubstituted aldehydes are used as substrates, fluorination of α-branched aldehydes with secondary amine catalysts generally exhibits low enantioselectivity.5a,5b To the best of our knowledge, there are only three reports on the enantioselective fluorination of α-branched aldehydes yielding tertiary fluorides with acceptable enantiopurity.^6^–^8^ Notably, Jørgensen and co-workers reported the asymmetric fluorination of α-alkyl-α-aryl aldehydes achieving high enantioselectivity (up to 90% ee) with a new primary amine catalyst with non-biaryl atropisomeric chirality.^6^ However, the isolated yields of the fluorinated products were not satisfactory for some reasons. Although we also reported the asymmetric fluorination of α-chloroaldehydes *via* the kinetic resolution mechanism, affording α-chloro-α-fluoroaldehydes with high enantioselectivities, moderate enantioselectivities were observed when α,α-dialkylaldehydes were employed.^7^ Here, we report the organocatalytic fluorination of α-branched aldehydes, using a newly developed chiral primary amine catalyst **1**; this approach affords the corresponding α-fluoroaldehydes in high chemical yields and enantioselectivities (Scheme 1). We also found that the resulting α-fluoroaldehydes could be converted into α-hydroxyacetals, bearing chiral tertiary alcohol moieties, and their optical purity could be maintained, which suggested that the reaction proceeded *via* a stereospecific C–F bond cleavage. These results shed new light on C–F bond activation,^9^ and will be useful because the resulting chiral tertiary alcohols may be valuable intermediates in the synthesis of biologically active compounds.

**Scheme 1:**
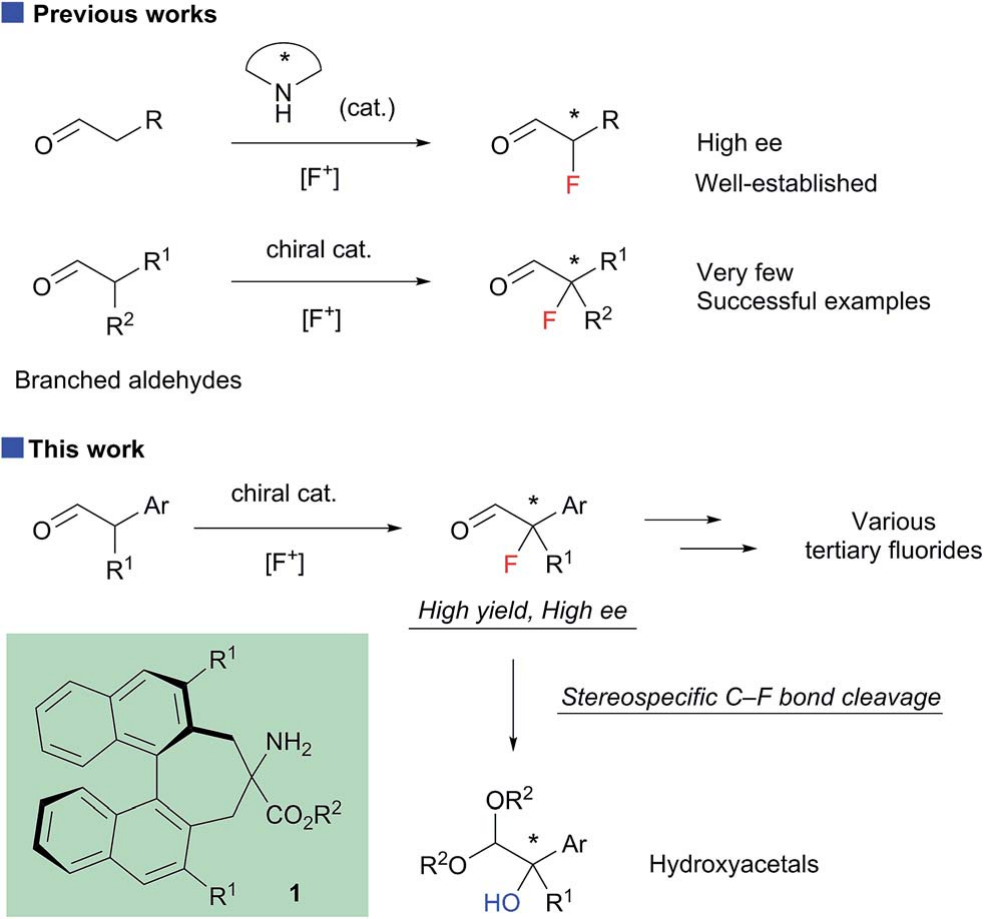
Asymmetric α-fluorination of aldehydes.

The structure of the new chiral primary amine catalyst **1** is shown in Scheme 2.^10^ An ester moiety and substituents at the 3,3′-positions on the binaphthyl backbone are expected to influence the chirality of the resulting products. Catalyst **1** was synthesized according to the procedure shown in Scheme 2. First, (*R*)-3,3′-diaryl-2,2′-bis(bromomethyl)-1,1′-binaphthyl (**2**) was prepared from commercially available (*R*)-BINOL *via* a reported procedure.^11^ Compound **2** was then converted into the desired amino ester **1***via* alkylative cyclization with ethyl isocyanoacetate and subsequent acid hydrolysis of the isocyano group.

**Scheme 2:**
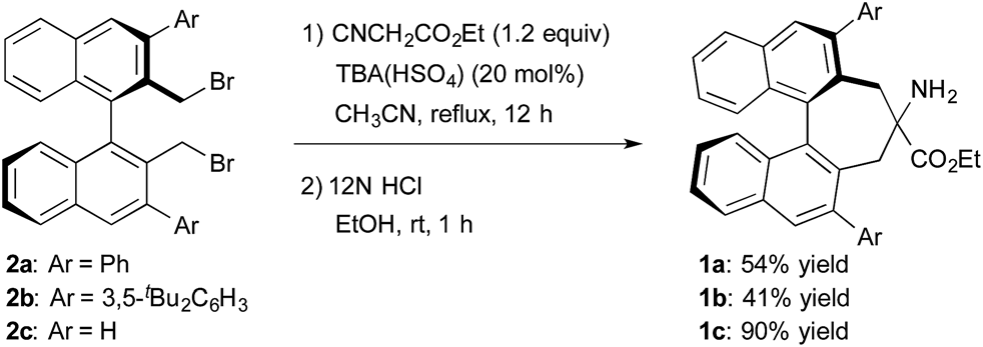
Synthesis of primary amine catalysts.

Next, **1** was applied in the enantioselective fluorination of α-branched aldehydes (Table 1). Fluorination of 2-phenylpropanal (**3a**) was carried out with *N*-fluorobenzenesulfonimide (NFSI) in the presence of 10 mol% **1a** to yield 2-fluoro-2-phenylpropanal (**4a**) in a high conversion. The fluorinated product was isolated after reduction to primary alcohol **5a**, due to difficulties in the purification of **4a**. Thus, **5a** was isolated in a sufficiently high chemical yield, but with poor enantioselectivity (entry 1). To our delight, the enantioselectivity of the fluorination dramatically improved to 90% ee by employing catalyst **1b**, which has bulky aryl substituents at the 3,3′-positions (entry 2). As expected, the use of catalyst **1c** without aryl substituents in the 3,3′-positions yielded a nearly racemic product (entry 3). The optimal solvent for the reaction was found to be toluene (entries 4–7). The enantioselectivity and reaction rate were slightly increased by adding 10 mol% 3,5-dinitrobenzoic acid as a co-catalyst (entry 10). We also confirmed that chiral primary amines **6** and **7**, which were reported to induce high enantioselectivity in the amination of α-branched aldehydes,^12^ were ineffective in the fluorination of **3a** (entries 11 and 12). The absolute configuration of **5a** was determined to be *S*, by comparison of its optical rotation with that of the reported value.^6^

**Table 1:** Optimization of reaction conditions^*a*^

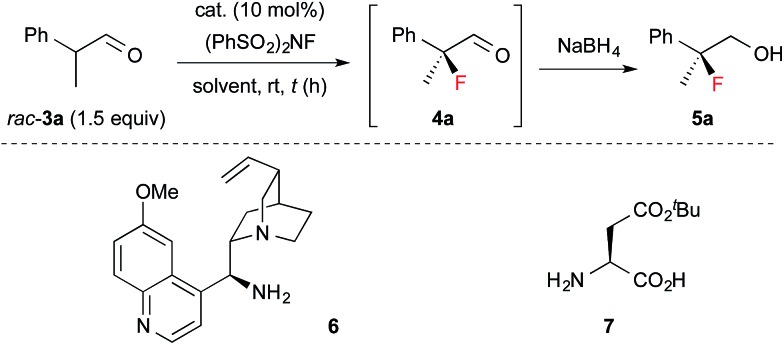					
Entry	Catalyst	Solvent	Time (h)	Yield^*b*^ (%)	ee^*c*^ (%)
1	**1a**	Toluene	24	79	51 (*S*)
2	**1b**	Toluene	2	97	90 (*S*)
3	**1c**	Toluene	24	71	3
4	**1b**	CH_2_Cl_2_	18	86	74 (*S*)
5	**1b**	EtOAc	4	99	82 (*S*)
6	**1b**	^*t*^BuOMe	3	97	86 (*S*)
7	**1b**	MeOH	48	<10	n.d.
8^*d*^	**1b**	Toluene	6	82	88 (*S*)
9^*e*^	**1b**	Toluene	48	73	93 (*S*)
10^*e*^ ^,^^*f*^	**1b**	Toluene	48	86	95 (*S*)
11^*g*^	**6**	CHCl_3_	24	76	13 (*R*)
12^*h*^	**7**	THF	2	98	13 (*S*)

^*a*^Reactions were carried out with 1.5 equiv. of *rac*-**3a** based on NFSI in the presence of 10 mol% **1** unless otherwise noted.

^*b*^Isolated yield of **5a**.

^*c*^Absolute configuration of the major enantiomer is specified in parenthesis.

^*d*^1.5 equiv. of NFSI was used based on *rac*-**3a**.

^*e*^At 0 °C.

^*f*^10 mol% 3,5-(NO_2_)_2_C_6_H_3_CO_2_H was used as a co-catalyst.

^*g*^5 mol% catalyst was used with 15 mol% TFA.

^*h*^20 mol% catalyst.

Encouraged by the results obtained with amine catalyst **1b**, we attempted to expand the substrate scope of the fluorination reaction. As summarized in Table 2, various α-alkyl-α-aryl aldehydes were successfully fluorinated to afford the corresponding α-fluoroaldehydes in high yields with high enantioselectivities. On the other hand, the reaction with α,α-dialkyl aldehyde **3o** yielded the product with good enantioselectivity but in poor yield, while the reaction with **3p** showed disappointingly low enantioselectivity. Although it was observed that the reaction with **3f** yield the corresponding fluoroaldehyde **4f** in good conversion by NMR measurement of the reaction mixture, reduction of **4f** to **5f** gave a complicated mixture, thus we could not determine those enantiopurity.

**Table 2:** Substrate scope of fluorination of **3**^*a*^

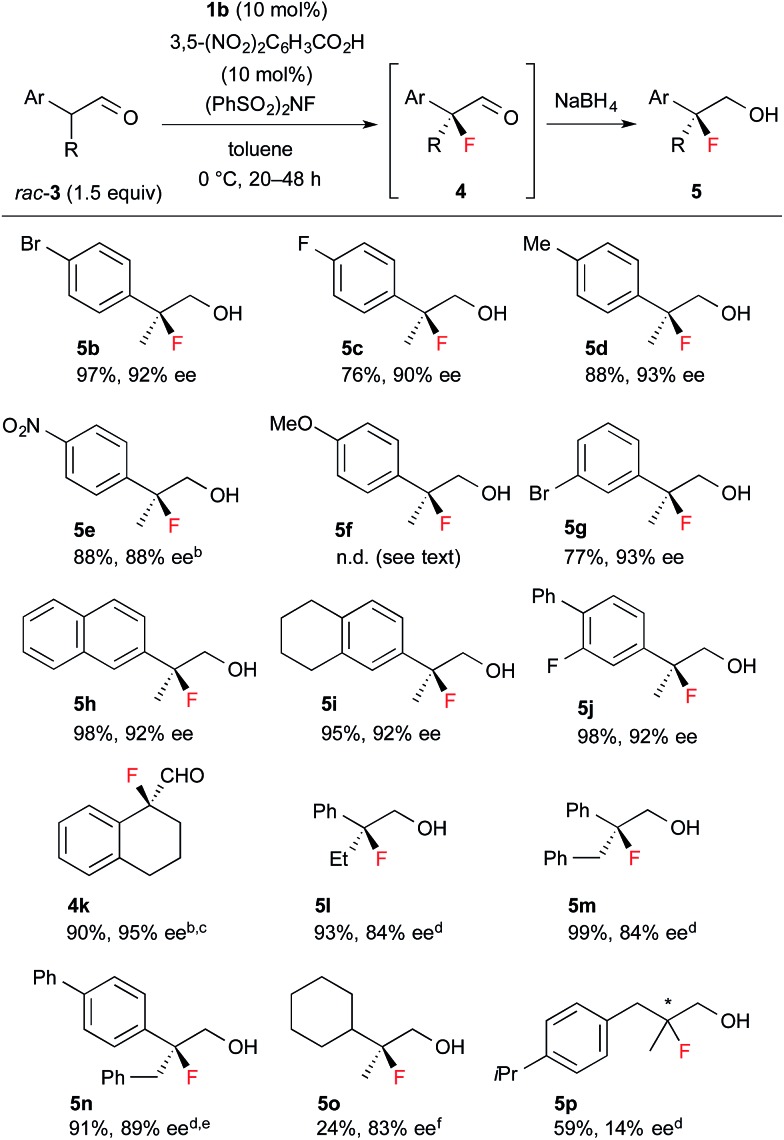

^*a*^Reactions were carried out with 1.5 equiv. of *rac*-**3** based on NFSI in the presence of 10 mol% **1b** and 3,5-(NO_2_)_2_C_6_H_3_CO_2_H. Isolated yield of **5** are described, except for **4k**.

^*b*^Purified product contained *ca.* 5% of an inseparable by-product.

^*c*^At rt. for 2 h.

^*d*^At rt. for 12–24 h.

^*e*^20 mol% catalyst.

^*f*^30 mol% catalyst.

The resulting fluorides can be converted into a variety of other tertiary fluorides (Scheme 3). First, allyl fluorides **8** were synthesized by Horner–Wadsworth–Emmons reaction of α-fluoroaldehydes **4** in good yield. Next, fluorohydrine **5j** was oxidized to carboxylic acid **9**,^13^ which is a fluorinated analogue of a non-steroidal anti-inflammatory agent, flurbiprofen.

**Scheme 3:**
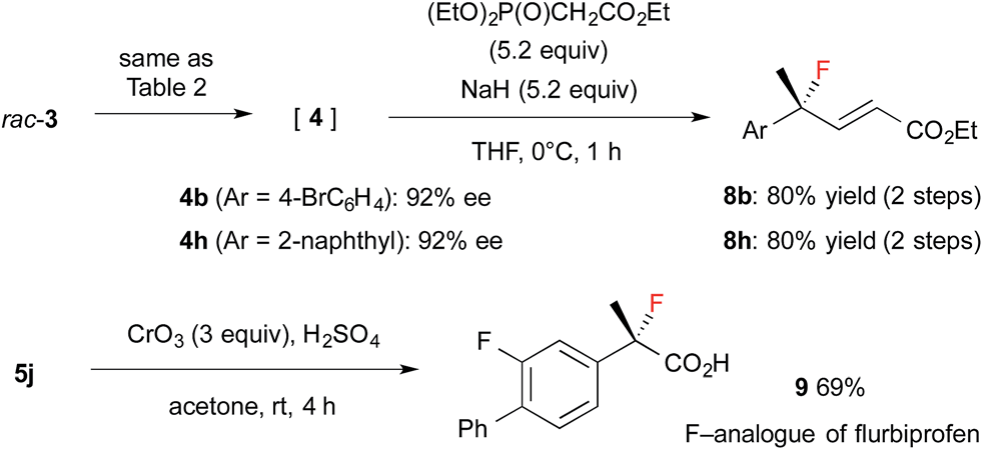
Synthesis applications of α-fluoroaldehydes.

We further investigated the synthetic utility of α-fluoroaldehydes **4**. Although, in general, the cleavage of carbon–fluorine bonds is not facile due to the strength of the bond, methods for C–F bond activation have recently garnered significant interest.^9^ The S_N_2-type nucleophilic substitution of sp^3^-alkylfluorides is known to be a challenging reaction; in particular, there are very few examples of the substitution of tertiary alkylfluorides.^14^ We recently reported that the S_N_2 reaction of α-chloro-α-keto esters with sodium azide and alkylthiols proceeds smoothly, despite the fact that the reaction occurs at a tertiary carbon.^15^ This finding encouraged us to examine the nucleophilic substitution of α-fluoroaldehydes **4**. First, typical nucleophiles such as sodium azide and alkylthiols were surveyed, but the desired product was not obtained. Eventually, we found that treatment of **4a** with NaOMe in methonal yielded the corresponding α-hydroxyacetal **10a** in a good conversion (Table 3).^16^ Due to the difficulties in purifying **4a**, enantioselective fluorination of **3a** and subsequent hydroxyacetalization were performed in a one-pot fashion. Notably, the enantiopurity of **10a** was nearly the same as that of **4a**. This result indicated that the C–F bond cleavage occurred in a stereospecific manner. As summarized in Table 3, various α-hydroxyacetals **10** were synthesized in good yields with high enantioselectivities *via* the sequential fluorination–alkaline treatment. When the second step was carried out with NaH in ethylene glycol, the corresponding α-hydroxy cyclic acetal **12** was obtained. The present method would be a good alternative to direct oxidation of α-branched aldehydes.^8^,^17^ Our method does not require the use of any explosive oxidant and simultaneously protects the carbonyl group. The resulting **10a** could be easily converted into α-hydroxy ester **13** without loss of enantiopurity (Scheme 4). The absolute configuration of **13** was determined to be *R*, by comparison of reported optical rotation values;^18^ these results confirmed that this transformation involved the Walden inversion.

**Table 3:** Asymmetric synthesis of α-hydroxyacetals **10**^*a*^

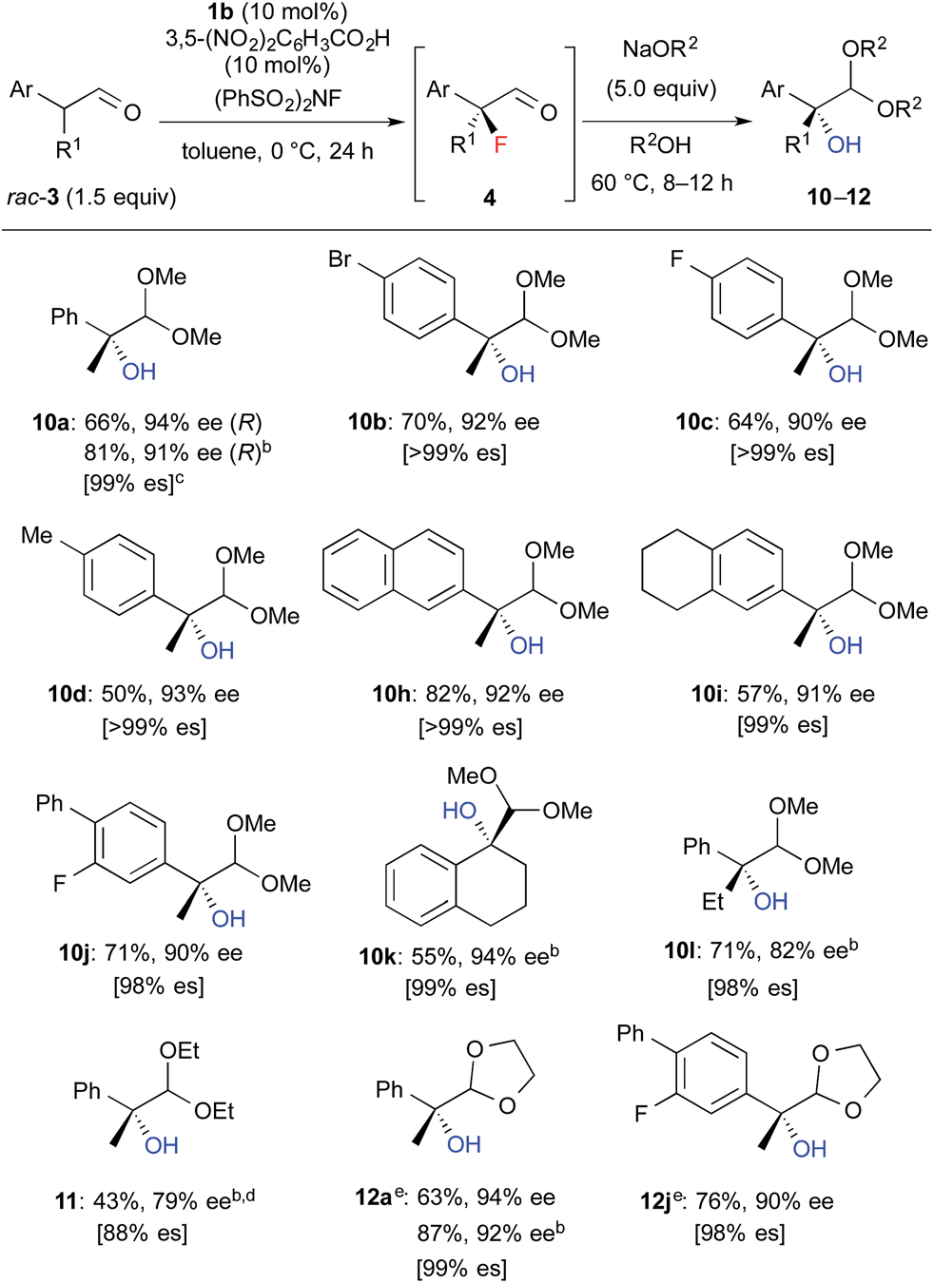

^*a*^Isolated yields of **10–12** from **3** are described.

^*b*^The first step was carried out at rt.

^*c*^es = (ee of **10–12**)/(ee of **4**).

^*d*^The second step was carried out at rt. under reflux conditions. Purified product contained *ca.* 10% of an inseparable by-product.

^*e*^The second step was carried out with NaH in ethylene glycol instead of NaOR^2^/R^2^OH.

**Scheme 4:**
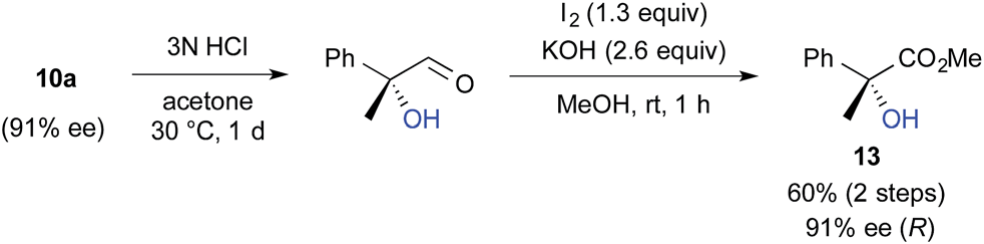
Synthesis of α-hydroxyesters.

The proposed reaction mechanism for the formation of hydroxyacetals **10** is shown in Scheme 5. ^1^H NMR studies revealed that α-fluoroaldehyde **4** is in equilibrium with hemiacetal I in d_4_-methanol. Upon treatment with NaOMe, epoxide II is formed *via* intramolecular S_N_2 displacement, which involves the stereospecific cleavage of C–F bond. Then, regeneration of the carbonyl moiety and subsequent acetalization or direct S_N_2-type ring opening of II with methoxide affords hydroxyacetal **10**.

**Scheme 5:**
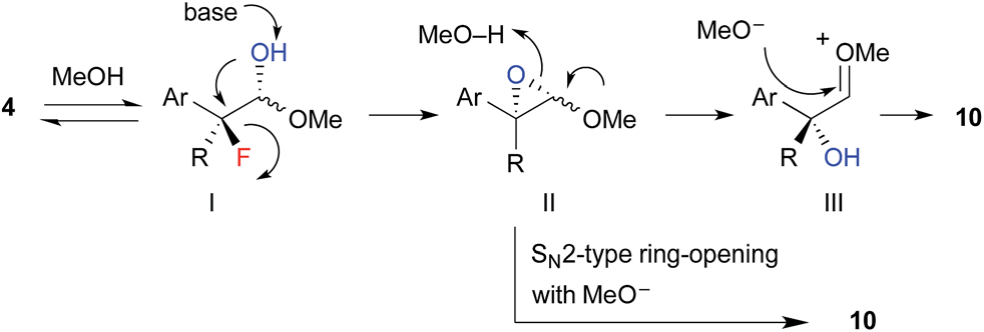
Proposed reaction mechanism.

## Conclusions

In conclusion, we developed a new class of chiral primary amine catalysts and successfully applied them in the enantioselective fluorination of α-branched aldehydes. Further, we found that the resulting fluoroaldehydes could be converted into the corresponding α-hydroxyacetals *via* stereospecific C–F bond cleavage.

## Supplementary Material

Supplementary information

## References

[cit1] (a) Gouverneur V. and Müller K., Fluorine in Pharmaceutical and Medicinal Chemistry, Imperial College Press, London, 2012.

[cit2] (d) Shibata N. Kohno J. Takai K. Ishimaru T. Nakamura S. Toru T. Kanemasa S., Angew. Chem., Int. Ed., 2005, 44 , 4204 , ; see also a very recent review on asymmetric fluorination: . 10.1002/anie.200501041 15942966

[cit3] Phipps R. J., Hiramatsu K., Toste F. D. (2012). J. Am. Chem. Soc..

[cit4] Bélanger É., Cantin K., Messe O., Tremblay M., Paquin J.-F. (2007). J. Am. Chem. Soc..

[cit5] Marigo M., Fielenbach D., Braunton A., Kjœrsgaard A., Jørgensen K. A. (2005). Angew. Chem., Int. Ed..

[cit6] Brandes S., Niess B., Bella M., Prieto A., Overgaard J., Jørgensen K. A. (2006). Chem. –Eur. J..

[cit7] Shibatomi K., Yamamoto H. (2008). Angew. Chem., Int. Ed..

[cit8] Very recently, new primary amine-catalyzed enantioselective fluorination of α-alkyl-α-aryl aldehydes achieving up to 86% ee was published, see: Witten M. R. Jacobsen E. N., Org. Lett., 2015, 17 , 2772 . 25952578 10.1021/acs.orglett.5b01193 PMC5098907

[cit9] Amii H., Uneyama K. (2009). Chem. Rev..

[cit10] Mazaleyrat J.-P., Gaucher A., Wakselman M., Tchertanov L., Guilhem J. (1996). Tetrahedron Lett..

[cit11] Ooi T., Kameda M., Maruoka K. (1999). J. Am. Chem. Soc..

[cit12] Liu C., Zhu Q., Huang K.-W., Lu Y. (2011). Org. Lett..

[cit13] Fujisawa H., Fujiwara T., Takeuchi Y., Omata K. (2005). Chem. Pharm. Bull..

[cit14] Zhang L., Zhang W., Liu J., Hu J. (2009). J. Org. Chem..

[cit15] Shibatomi K., Narayama A., Soga Y., Muto T., Iwasa S. (2011). Org. Lett..

[cit16] For the formation of α-hydroxyacetals with α-chloroaldehydes, see: Arasaki H. Iwata M. Nishimura D. Itoh A. Masaki Y., Synlett, 2004 , 546 .

[cit17] Demoulin N., Lifchits O., List B. (2012). Tetrahedron.

[cit18] Wieland L. C., Deng H., Snapper M. L., Hoveyda A. H. (2005). J. Am. Chem. Soc..

