# Supplementary material for: Enantioselective fluorination of α-branched aldehydes and subsequent conversion to α-hydroxyacetals via stereospecific C–F bond cleavage
Source: Chem Sci. 2015 Nov 16;7(2):1388–92. doi: 10.1039/c5sc03486h (PMC5975931; doi:10.1039/c5sc03486h)
Supplement: Supplementary file 1 [file SC-007-C5SC03486H-s001.pdf]

Electronic Supplementary Material (ESI) for Chemical Science.
This journal is © The Royal Society of Chemistry 2015

Enantioselective Fluorination of α-Branched Aldehydes and Subsequent Conversion to α-Hydroxyacetals via Stereospecific C–F Bond Cleavage

Kazutaka Shibatomi,* Kazumasa Kitahara, Takuya Okimi, Yoshiyuki Abe, Seiji Iwasa

Department of Environmental and Life Sciences, Toyohashi University of Technology, 1-1 Hibarigaoka, Tempaku-cho, Toyohashi 441-8580, Japan

shiba@ens.tut.ac.jp

Supplementary Information

Table of contents

General methods 2

Synthesis of chiral primary amine catalysts **1** (Scheme 2). 2

Highly enantioselective fluorination of α-branched aldehydes (Table 2). 6

Derivatization of α-fluoroaldehydes (Scheme 3). 16

Synthesis of α-hydroxyacetals **10** (Table 3). 18

^{1}H NMR measurement of hemiacetal derived from **4a**. 25

Synthesis of (*R*)-α-hydroxyester **13** (Scheme 4). 25

References 26

NMR spectra and HPLC traces 27

General methods: All non-aqueous reactions were carried out in flame-dried glassware under argon atmosphere and stirred using magnetic stir-plates. Thin-layer chromatography analyses were performed using Merck pre-coated silica gel plates with 254 indicator. Visualization was accomplished by UV light (254 nm), potassium permanganate, phosphomolybdic acid, or anisaldehyde. Flash column chromatography was performed using silica gel 60 (mesh 230-400) supplied by Kanto Chemical Co., Inc. ^{1}H, ^{13}C, and ^{19}F NMR spectra were recorded on a JEOL JNM-ECX400 (400 MHz ^{1}H, 100 MHz ^{13}C, 376 MHz ^{19}F) or a JEOL JNM-ECX500 (500 MHz ^{1}H, 126 MHz ^{13}C, 470 MHz ^{19}F). Chemical shift values (δ) are reported in ppm (tetramethylsilane δ 0.00 ppm, residual benzene δ 7.15 ppm or methanol δ 3.31 for ^{1}H; hexafluorobenzene δ –162.20 ppm for ^{19}F; residual chloroform δ 77.0 ppm, benzene δ 128.0 or methanol δ 49.0 ppm for ^{13}C). Optical rotations were measured on a JASCO P-1030 digital polarimeter. GC analysis was performed with a Shimadzu model 2014 instrument. Analytical HPLC was performed on a JASCO PU1586 with a UV-1575 UV/Vis detector using a chiral column. DART mass (positive mode) analyses were performed on a LC-TOF JMS-T100LP. We confirmed that the optical purity of selected products **5a** and **10a** did not change even after chromatographic purification using silica gel and subsequent solvent evaporation.[1]

Materials: Commercial grade reagents and solvents were used without further purification unless otherwise noted. Anhydrous *t*-butyl methyl ether (TBME), ethyl acetate, dimethylformamide (DMF), methanol and ethylene glycol were purchased from Aldrich. Anhydrous acetonitrile and acetone were purchased from Wako Pure Chemical Industries, Ltd. Anhydrous toluene, dichloromethane, tetrahydrofurane (THF), and benzene were purchased from Kanto Chemical Co. Inc. and used after purification by GLASS-Contour Solvent Dispensing System, but benzene was used without purification. Chiral primary amine catalysts **1c** was synthesized from (*R*)-BINOL according to the reported procedure.[2]

<u>**Synthesis of chiral primary amine catalysts 1 (Scheme 2).**</u>

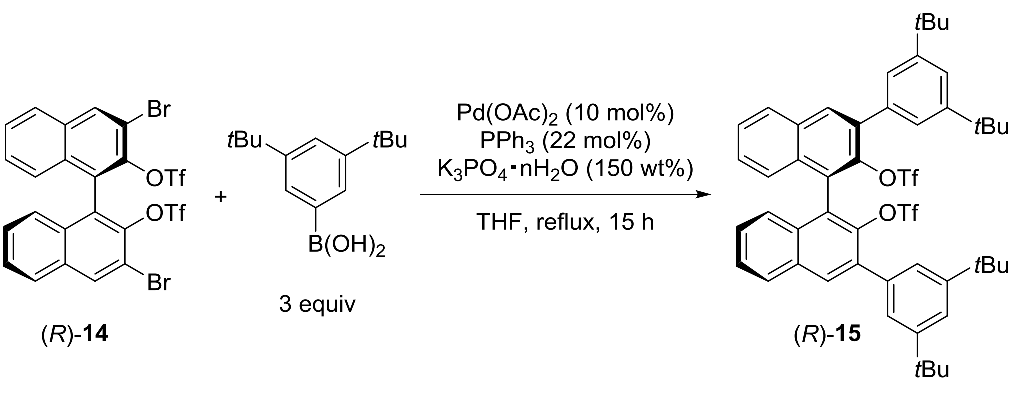

(*R*)-**14**　　(*R*)-**15**

A solution of (R)-**14**[3] (3 mmol), 3,5-di-*t*-Bu-phynylboronic acid (9 mmol, 3 equiv), $Pd(OAc)_2$ (67.4 mg, 0.3 mmol, 10 mol%), PPh_3 (173.1 mg, 0.66 mmol, 22 mol%), and $K_3PO_4 \cdot nH_2O$ (150 wt%) in dry THF (30 mL) was degassed by bubbling argon through this solution for 30 min. The solution was refluxed for 15 h under argon atmosphere. The resulting mixture was poured into saturated aq.NH_4Cl, and the whole mixture was filtered to remove the catalyst, then extracted with ethyl acetate. The organic extracts were dried over Na_2SO_4 and concentrated. The crude mixture was purified by silica gel column chromatography (hexane : CH_2Cl_2 = 5:1) to give 75% yield of (R)-**15** (white solid). ^{1}H NMR (400 MHz, $CDCl_3$): δ 8.14 (s, 2H), 7.99 (d, J = 7.9 Hz, 2H), 7.58–7.36 (m, 12H), 1.40 (s, 36H); ^{13}C NMR (100 MHz, $CDCl_3$): δ 150.9, 144.3, 135.5, 135.3, 132.9, 132.5, 132.5, 128.3, 127.6, 127.4, 127.3, 125.5, 124.0, 122.2, 119.2, 116.0, 34.9, 31.3; ^{19}F NMR (376MHz, $CDCl_3$): δ –75.8; $[\alpha]_D^{23}$ –194.3 (c = 6.4, $CHCl_3$); HRMS (DART): Anal. For $C_{50}H_{53}F_6O_6S_2^{+1}$ $[M+H]^+$ Calcd.: 927.3188, Found: 927.3185.

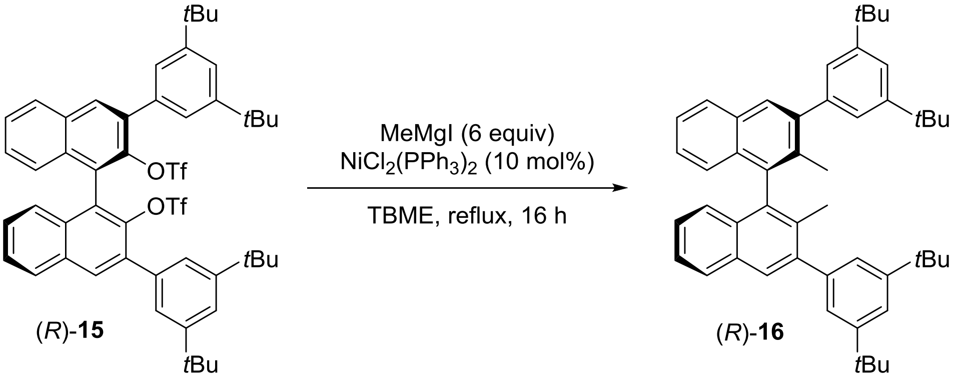

(R)-**15** (R)-**16**

To a solution of (R)-**15** (1.46 mmol) and $NiCl_2(PPh_3)_2$ (95.5 mg, 0.146 mmol, 10 mol%) in TBME (14.6 mL) was added 3M ethereal solution of MeMgI (2.92 mL, 8.76 mmol, 6 equiv) at 0 ºC. The solution was refluxed for 16 h under argon atmosphere. This mixture was poured into ice-cooled 1M HCl, and the whole mixture was filtered to remove the catalyst. The filtrate was poured into saturated aq.$NaHCO_3$, and extracted with dichloromethane. The organic extracts were dried over Na_2SO_4 and concentrated. The crude mixture was purified by silica gel column chromatography (hexane : CH_2Cl_2 = 10:1) to give 91% yield of (R)-**16** (white solid). ^{1}H NMR (500 MHz, $CDCl_3$): δ 7.94–7.87 (m, 4H), 7.48–7.45 (m, 2H), 7.41–7.38 (m, 2H), 7.36–7.34 (m, 4H), 7.25–7.18 (m, 4H), 2.03 (s, 6H), 1.40 (s, 36H); ^{13}C NMR (126 MHz, $CDCl_3$): δ 150.2, 142.4, 141.4, 136.6, 132.9, 132.1, 132.0, 128.2, 127.9, 126.0, 125.8, 125.3, 123.9, 120.7, 35.0, 31.6, 18.4; $[\alpha]_D^{21}$ +51.5 (c = 9.5, $CHCl_3$); HRMS (DART): Anal. For $C_{50}H_{58}^{+1}$ $[M+H]^+$ Calcd.: 659.4617, Found: 659.4616.

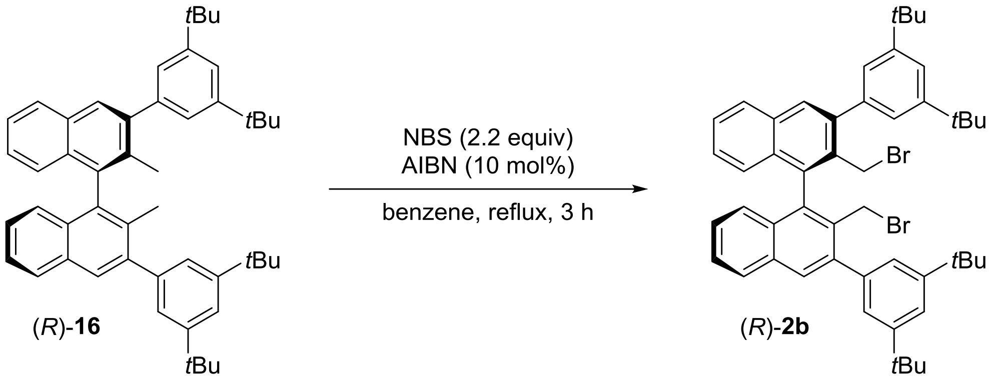

A solution of (*R*)-**16** (3.24 mmol), *N*-bromosuccinimide (NBS) (1.27 g, 7.13 mmol, 2.2 equiv), and 2,2'-azobis(isobutyronitrile) (AIBN) (53.2 mg, 0.324 mmol, 10 mol%) in benzene (16.2 mL) was refluxed for 3 h. After being cooled to room temperature, the mixture was poured into water and extracted with ethyl acetate. The organic extracts were dried over Na_2SO_4 and concentrated. The crude mixture was purified by silica gel column chromatography (hexane : CH_2Cl_2 = 10:1) to give 97% yield of (*R*)-**2b** (white solid). ^{1}H NMR (400 MHz, $CDCl_3$): δ 7.98–7.88 (m, 4H), 7.50–7.46 (m, 8H), 7.29–7.18 (m, 4H), 4.29 (s, 4H), 1.40 (s, 36H); ^{13}C NMR (100 MHz, $CDCl_3$): δ 150.3, 142.2, 139.6, 136.5, 133.2, 132.5, 131.8, 130.1, 127.8, 127.4, 127.1, 126.2, 124.0, 121.2, 35.0, 32.7, 31.6; $[\alpha]_D^{22}$ +31.7 (c = 9.4, $CHCl_3$); HRMS (DART): Anal. For $C_{50}H_{57}Br_2^{+1}$ $[M+H]^+$ Calcd.: 815.2827, Found: 815.2827.

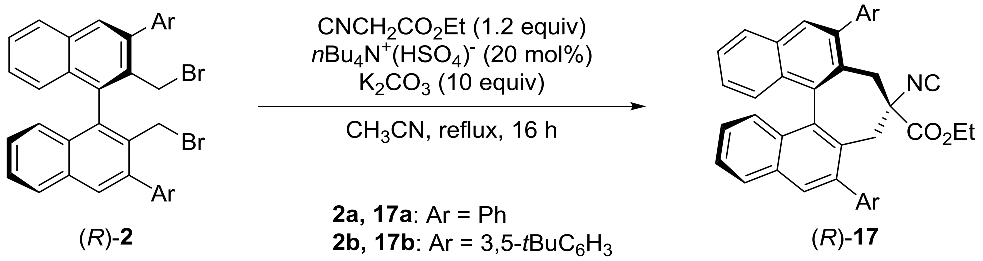

To a suspension of (*R*)-**2a**[3] or (*R*)-**2b** (5.25 mmol), tetrabutylammonium hydrogen sulfate (356.5 mg, 1.05 mmol, 20 mol%) and K_2CO_3 (7.26 g, 52.5 mmol, 10 equiv) in CH_3CN (105 mL) was added ethyl isocyanoacetate (688 μL, 6.30 mmol, 1.2 equiv) at 0 ºC. The solution was refluxed for 16 h under argon atmosphere. The resulting mixture was filtered and the filtrate was concentrated. The residue was purified by column chromatography on silica gel to afford (*R*)-**17**.

(*R*)-**17a**: The crude mixture was purified by silica gel column chromatography (hexane : ethyl acetate = 10 : 1) to give 69% of (*R*)-**17a** (white solid). ^{1}H NMR (400 MHz, $CDCl_3$): δ 7.95 (s, 1H), 7.93 (dd, *J* = 22.9, 8.2 Hz, 2H), 7.82 (s, 1H), 7.51–7.20 (m, 16H), 3.88–3.80 (m, 1H), 3.77 (d, *J* = 13.7 Hz, 1H), 3.56–3.48 (m, 1H), 3.32 (d, *J* = 14.3 Hz, 1H), 3.09 (d, *J* = 14.3 Hz, 1H),

2.84 (d, J = 14.0 Hz, 1H), 0.90 (t, J = 7.3 Hz, 3H); ^{13}C NMR (100 MHz, $CDCl_3$): δ 166.7, 159.8, 141.0, 140.6, 139.8, 136.0, 135.5, 131.2, 131.1, 130.6, 130.1, 130.1, 129.6, 129.4, 128.3, 128.2, 128.2, 128.1, 127.2, 127.2, 127.1, 127.0, 126.2, 126.1, 126.1, 126.1, 70.7, 62.6, 38.9, 34.8. 13.4; $[\alpha]_D^{30}$ +3.5 (c = 0.74, $CHCl_3$); HRMS (DART): Anal. For $C_{39}H_{30}N_1O_2^{+1}$ $[M+H]^+$ Calcd.: 544.2277, Found: 544.2280.

(*R*)-**17b**: The crude mixture was purified by silica gel column chromatography (hexane : CH_2Cl_2 = 3:1) to give 48% yield of (*R*)-**17b** (white solid). ^{1}H NMR (400 MHz, $CDCl_3$): δ 7.98–7.92 (m, 3H), 7.88 (s, 1H), 7.65–7.14 (m, 12H), 4.00 (d, J = 14.0 Hz, 1H), 3.76–3.68 (m, 1H), 3.37–3.27 (m, 2H), 3.06 (d, J = 14.3 Hz, 1H), 2.91 (d, J = 14.0 Hz, 1H), 1.37 (s, 36H), 0.82 (t, J = 7.0 Hz, 3H); ^{13}C NMR (100 MHz, $CDCl_3$): δ 166.8, 160.2, 150.5, 142.2, 140.7, 139.8, 139.6, 136.3, 135.6, 133.0, 132.6, 131.1, 131.0, 130.5, 129.8, 129.5, 129.5, 128.2, 127.3, 127.1, 126.1, 126.0, 125.9, 125.8, 124.7, 121.2, 120.9, 70.6, 62.1, 39.0, 34.9, 34.6, 31.5; $[\alpha]_D^{23}$ +13.0 (c = 6.5, $CHCl_3$); HRMS (DART): Anal. For $C_{55}H_{62}N_1O_2^{+1}$ $[M+H]^+$ Calcd.: 768.4781, Found: 768.4781.

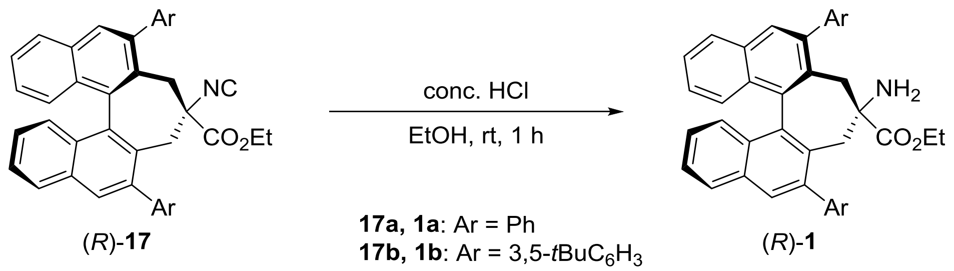

To a solution of (*R*)-**17** (2.3 mmol) in ethanol (230 mL) was added conc. HCl (6.1 mL) at 0 ºC. The solution was stirred at room temperature for 1 h under argon atmosphere. The resulting mixture was poured into ice-cooled saturated aq.$NaHCO_3$ and extracted with CH_2Cl_2. The organic extracts were dried over Na_2SO_4 and concentrated. The residue was purified by column chromatography on silica gel to afford (*R*)-**1**.

(*R*)-**1a**: The crude mixture was purified by silica gel column chromatography (hexane : ethyl acetate = 3 : 1) to afford 79% of (*R*)-**1a** (white solid). ^{1}H NMR (500 MHz, C_6D_6): δ 7.81 (d, J = 23.7 Hz, 2H), 7.74 (dd, J =16.8, 8.0 Hz, 2H), 7.60 (dd, J = 8.4, 3.1 Hz, 2H), 7.39–7.09 (m, 12H), 7.05–6.99 (m, 2H), 3.66–3.60 (m, 1H), 3.46 (d, J = 13.8 Hz, 1H), 3.40–3.34 (m, 2H), 3.12 (d, J = 13.8 Hz, 1H), 2.51 (d, J = 13.4 Hz, 1H), 0.98 (s, 2H), 0.58 (t, J = 6.9 Hz, 3H); ^{13}C NMR (125 MHz, C_6D_6): δ 174.4, 142.5, 142.1, 142.0, 140.7, 136.2, 135.9, 134.0, 133.9, 133.0, 132.9, 132.0, 131.9, 130.7, 130.6, 129.5, 129.3, 128.7, 128.3, 128.2, 128.1, 127.8, 127.7, 127.6, 127.1, 126.9, 126.2, 126.2, 126.0, 125.9, 68.8, 60.6, 40.0, 36.6; $[\alpha]_D^{28}$ –16.8 (c = 0.9, $CHCl_3$); HRMS (DART): Anal. For. $C_{38}H_{32}N_1O_2^{+1}$ $[M+H]^+$ Calcd.: 534.2433, Found: 534.2431.

(*R*)-**1b**: The crude mixture was purified by silica gel column chromatography (hexane : CH_2Cl_2 = 1:1) to give 86% yield of (*R*)-**1b** (white solid). ^{1}H NMR (400 MHz, C_6D_6): δ 8.02 (s, 2H), 7.76 (dd, *J* = 8.4, 8.0 Hz, 2H), 7.63–7.51 (m, 8H), 7.24–7.18 (m, 2H), 7.01–6.94 (m, 2H), 3.83 (d, *J* = 13.4 Hz, 1H), 3.60–3.54 (m, 1H), 3.49 (d, *J* = 13.7 Hz, 1H), 3.36–3.28 (m, 1H), 3.24 (d, *J* = 13.7 Hz, 1H), 2.69 (d, *J* = 13.4 Hz, 1H), 1.31 (s, 36H), 0.55 (t, *J* = 7.6 Hz, 3H); ^{13}C NMR (100 MHz, C_6D_6): δ 174.2, 150.5, 143.0, 142.0, 141.6, 141.4, 136.5, 136.1, 134.3, 134.0, 133.2, 132.9, 132.0, 132.0, 129.9, 129.1, 128.6, 126.2, 126.1, 125.9, 125.9, 125.5, 125.4, 120.9, 120.6, 68.8, 60.4, 40.3, 36.6, 35.0, 31.6, 13.7; The enantiopurity was determined by HPLC (hexane : 2-propanol = 300 : 1; 0.5 mL/min; using a CHIRALPAK IB-3 column (0.46 cmφ × 25 cm)): 8.7 min (minor) and 9.4 min (major); $[\alpha]_D^{27}$ –14.1 (c = 1.1, $CHCl_3$); HRMS (DART): Anal. For $C_{54}H_{64}N_1O_2^{+1}$ $[M+H]^+$ Calcd.: 758.4937, Found: 758.4935.

<u>Highly enantioselective fluorination of α-branched aldehydes 3 (Table 2).</u>

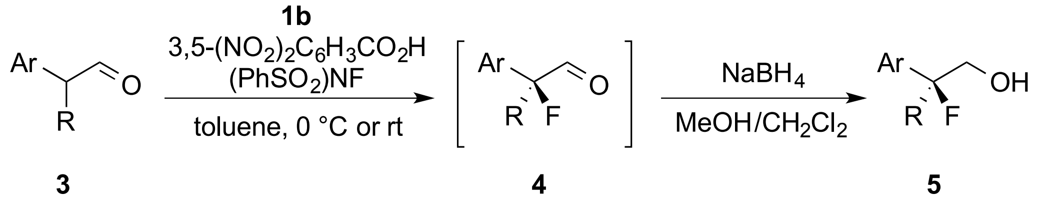

General procedure for fluorination of α-branched aldehydes: All of aldehydes were purified before the reactions by flash column chromatography on silica gel or Kugelrohr distillation. To a solution of catalyst **1b** (20 mg, 0.026 mmol, 10 mol%) in toluene (0.54 mL) was added 3,5-dinitrobenzoic acid (5.5 mg, 0.026 mmol, 10 mol%), aldehydes **3** (0.39 mmol, 1.5 equiv), and N-fluorobenzenesulfonimide (NFSI) (0.26 mmol, 82 mg, 1 equiv) at 0 °C. The reaction mixture was stirred at room temperature or at 0 °C, then poured into $MeOH/CH_2Cl_2$ (1 : 4, 1.3 mL) at 0 °C. To this solution, $NaBH_4$ (1.6 mmol, 6 equiv) was added, and the mixture was stirred at room temperature for 1 h. The reaction was quenched with saturated aq.NH_4Cl, and the mixture was extracted with Et_2O. The organic layer was dried over Na_2SO_4, concentrated, and chromatographed on silica gel to give **5**.

(*S*)-2-fluoro-2-phenylpropan-1-ol [(*S*)-5a, 95% *ee*]

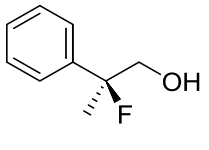

The reaction was carried out at 0 °C and stirred for 48 h. The crude mixture was purified by silica gel column chromatography (hexane : ethyl acetate = 3:1) to give 86% yield of (*S*)-**5a** (white solid). ^{1}H NMR (400 MHz, $CDCl_3$): δ 7.40–7.27 (m, 5H), 3.88–3.69 (m, 2H), 2.50 (s,

1H), 1.69 (d, J = 23.2 Hz, 3H); ^{13}C NMR (100 MHz, $CDCl_3$): δ 141.5 (d, J = 21.1 Hz), 128.4, 127.8, 124.4 (d, J = 9.6 Hz), 97.8 (d, J = 172.5 Hz), 69.5 (d, J = 24.9 Hz), 23.1 (d, J = 24.0 Hz); ^{19}F NMR (376 MHz, $CDCl_3$): δ –157.6 (m); $[\alpha]_D^{22}$ +14.3 (c = 0.87, $CHCl_3$); HRMS (DART): Anal. For $C_9H_{11}F_1O_1{}^{+1}$ $[M+NH_4]^+$ Calcd.: 172.1138, Found: 172.1135. The enantiopurity was determined after conversion into the corresponding benzoate (*S*)-**18a**. The absolute configuration of the major enantiomer was determined to be *S* by comparing the specific rotation with that in the literature.[4]

General procedure for benzoylation of 5: A flame-dried flask under argon was charged with **5** (0.30 mmol) and CH_2Cl_2 (1.0 mL). Triethylamine (0.60 mmol), benzoyl chloride (0.45 mmol), and 4-dimethylaminopyridine (0.03 mmol) were added to this solution, and the mixture was stirred for 1 h at 0 °C. The mixture was diluted by saturated aq.$NaHCO_3$, and extracted with CH_2Cl_2. The organic layer was dried over Na_2SO_4 and concentrated under reduced pressure. The crude mixture was purified by silica gel column chromatography to afford **18**.

(*S*)-2-fluoro-2-phenylpropyl benzoate [(*S*)-18a, 95% *ee*]

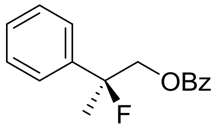

The crude mixture was purified by flash column chromatography on silica gel (hexane : ethylacetate = 20:1) to afford the desired benzoate (*S*)-**18a** in 85 % yield (white solid). ^{1}H NMR (400 MHz, $CDCl_3$): δ 8.01 (dd, J = 8.2, 1.2 Hz, 2H), 7.58–7.54 (m, 1H), 7.46–7.38 (m, 6H), 7.36–7.32 (m, 1H), 4.63–4.50 (m, 2H), 1.81 (d, J = 22.3 Hz, 3H); ^{13}C NMR (100 MHz, $CDCl_3$): δ 166.1, 141.1 (d, J = 22.0 Hz), 133.1, 129.7, 128.4, 128.4, 128.0, 124.5, 124.4, 95.6 (d, J = 176.4 Hz), 69.7 (d, J = 24.9 Hz), 23.6 (d, J = 24.9 Hz); ^{19}F NMR (376 MHz, $CDCl_3$): δ –153.8 (m); $[\alpha]_D^{20}$ +12.1 (c = 1.6, $CHCl_3$); HRMS (DART): Anal. For $C_{16}H_{15}F_1O_2{}^{+1}$ $[M+NH_4]^+$ Calcd.: 276.1400, Found: 276.1400. The enantiopurity was determined by HPLC (hexane : 2-propanol = 99 : 1; 0.5 mL/min; using a CHIRALPAK ID column (0.46 cmφ × 25 cm)): 16.7 min (major) and 19.0 min (minor).

2-(4-bromophenyl)-2-fluoropropan-1-ol (5b, 92% *ee*)

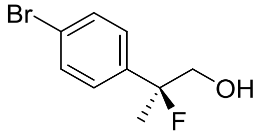

The reaction was stirred for 20 h at 0 °C. The crude mixture was purified by silica gel column chromatography (hexane : diethyl ether = 2:1) to give 98% yield of **5b** (white solid). ^{1}H NMR

(400 MHz, $CDCl_3$): δ 7.52 (d, J = 8.9 Hz, 2H), 7.24 (d, J = 8.5 Hz, 2H), 3.87–3.70 (m, 2H), 1.83 (t, J = 6.6 Hz, 1H), 1.68 (d, J = 22.6 Hz, 3H); ^{13}C NMR (100 MHz, $CDCl_3$): δ 140.6 (d, J = 22.0 Hz), 131.6, 126.3 (d, J = 9.6 Hz), 121.9, 97.5 (d, J = 172.5 Hz), 69.3 (d, J = 24.9 Hz), 23.1 (d, J = 24.9 Hz); ^{19}F NMR (376 MHz, $CDCl_3$): δ –157.8 (m); $[\alpha]_D^{20}$ +18.4 (c = 0.31, $CHCl_3$); HRMS (DART): Anal. For $C_9H_{10}Br_1F_1O_1^{+1}$ $[M+NH_4]^+$ Calcd.: 250.0243, Found: 250.0245. The enantiopurity was determined after conversion into the corresponding benzoate **18b**.

2-(4-bromophenyl)-2-fluoropropyl benzoate (18b, 92% *ee*)

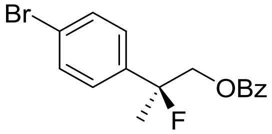

According to the general procedure, **5b** was converted into **18b**, the crude mixture was purified by flash column chromatography on silica gel (hexane : ethylacetate = 20:1) to afford the desired benzoate **18b** in 93 % yield (white solid). ^{1}H NMR (400 MHz, $CDCl_3$): δ 7.99 (d, J = 7.3 Hz, 2H), 7.58–7.51 (m, 3H), 7.45–7.41 (m, 2H), 7.32 (d, J = 8.5 Hz, 2H), 4.63–4.48 (m, 2H), 1.79 (d, J = 22.3 Hz, 3H); ^{13}C NMR (100 MHz, $CDCl_3$): δ 165.9, 140.2 (d, J = 22.0 Hz), 133.3, 131.6, 129.7, 129.5, 128.4, 126.3 (d, J = 9.6 Hz), 122.2, 95.3 (d, J = 176.4 Hz), 69.3 (d, J = 25.9 Hz), 23.6 (d, J = 24.0 Hz); ^{19}F NMR (376 MHz, $CDCl_3$): δ –153.9 (m); $[\alpha]_D^{29}$ +19.0 (c = 1.6, $CHCl_3$); HRMS (DART): Anal. For $C_{16}H_{14}Br_1F_1O_2^{+1}$ $[M+NH_4]^+$ Calcd.: 354.0505, Found: 354.0503. The enantiopurity was determined by HPLC (hexane : 2-propanol = 99 : 1; 0.5 mL/min; using a CHIRALPAK ID column (0.46 cmφ × 25 cm)): 17.8 min (major) and 21.2 min (minor).

2-fluoro-2-(4-fluorophenyl)propan-1-ol [5c, 90% *ee*]

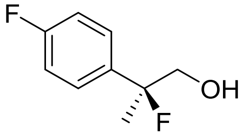

The reaction was carried out at 0 °C and stirred for 48 h. The crude mixture was purified by silica gel column chromatography (hexane : diethyl ether = 2:1–1:1) to give 76% yield of **5c** (white solid; including ca. 3% of an inseparable by-product). ^{1}H NMR (500 MHz, $CDCl_3$): δ 7.34–7.32 (m, 2H), 7.08–7.04 (m, 2H), 3.85–3.69 (m, 2H), 1.94 (bs, 1H), 1.68 (d, J = 22.6 Hz, 3H); ^{13}C NMR (126 MHz, $CDCl_3$): δ 162.3 (d, J = 247.1 Hz), 137.3 (dd, J = 22.2, 3.0 Hz), 126.3 (t, J = 8.4 Hz), 115.3 (d, J = 21.6 Hz), 97.6 (d, J = 171.5 Hz), 69.5 (d, J = 26.4 Hz), 23.2 (d, J = 25.2 Hz); ^{19}F NMR (470 MHz, $CDCl_3$): δ –115.2 (s), –156.3 (m); $[\alpha]_D^{23}$ +10.9 (c = 0.80, $CHCl_3$); HRMS (DART): Anal. For $C_9H_{10}F_2O_1^{+1}$ $[M+NH_4]^+$ Calcd.: 190.1044, Found: 190.1044. The enantiopurity was determined by HPLC (hexane : 2-propanol = 20 : 1; 1.0

mL/min; using a CHIRALPAK AD-H column (0.46 cmφ × 25 cm)): 11.5 min (minor) and 12.8 min (major).

2-fluoro-2-(p-tolyl)propan-1-ol [5d, 93% *ee*]

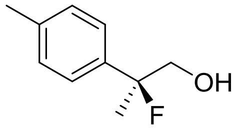

The reaction was carried out at 0 °C and stirred for 48 h. The crude mixture was purified by silica gel column chromatography (hexane : diethyl ether = 2:1–1:1) to give 88% yield of **5d** (white solid). ^{1}H NMR (500 MHz, $CDCl_3$): δ 7.25 (d, J = 8.0 Hz, 2H), 7.19 (d, J = 8.0 Hz, 2H), 3.86–3.68 (m, 2H), 2.35 (s, 3H), 1.92 (t, J = 6.1 Hz, 1H), 1.68 (d, J = 22.6 Hz, 3H); ^{13}C NMR (126 MHz, $CDCl_3$): δ 138.5 (d, J = 21.6 Hz), 137.6, 129.1, 124.4 (d, J = 8.4 Hz), 97.8 (d, J = 171.5 Hz), 69.6 (d, J = 25.2 Hz), 23.1 (d, J = 24.0 Hz), 21.0; ^{19}F NMR (470 MHz, $CDCl_3$): δ –157.0 (m); $[\alpha]_D^{23}$ +14.4 (c = 1.25, $CHCl_3$); HRMS (DART): Anal. For $C_{10}H_{13}F_1O_1^{+1}$ $[M+H]^+$ Calcd.: 169.1029, Found: 169.1029. The enantiopurity was determined by HPLC (hexane : 2-propanol = 20 : 1; 1.0 mL/min; using a CHIRALPAK AD-H column (0.46 cmφ × 25 cm)): 12.2 min (major) and 14.7 min (minor).

2-fluoro-2-(4-nitrophenyl)propan-1-ol (5e, 88% *ee*)

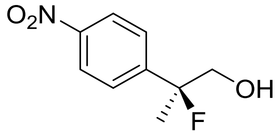

The reaction was stirred for 48 h at 0 °C. The crude mixture was purified by silica gel chromatography (hexane : diethyl ether = 1 : 1–1 : 2) to give 88% yield of **5e** (white solid; including small amount of inseparable by-product). ^{1}H NMR (500 MHz, $CDCl_3$): δ 8.25 (d, J = 8.4 Hz, 2H), 7.55 (d, J = 8.8 Hz, 2H), 3.91–3.80 (m, 2H), 2.16 (s, 1H), 1.72 (d, J = 22.6 Hz, 3H); ^{13}C NMR (126 MHz, $CDCl_3$): δ 148.9 (d, J = 21.6 Hz), 147.4, 125.6 (d, J = 9.6 Hz), 123.6, 97.5 (d, J = 173.9 Hz), 69.0 (d, J = 25.2 Hz), 23.2 (d, J = 24.0 Hz); ^{19}F NMR (470 MHz, $CDCl_3$): δ –158.0 (m); $[\alpha]_D^{28}$ +19.0 (c = 1.1, $CHCl_3$); HRMS (DART): Anal. For $C_{19}H_{10}FNO_3^{+1}$ $[M+NH_4]^+$ Calcd.: 217.0988, Found: 217.0989. The enantiopurity was determined by HPLC (hexane : 2-propanol = 9 : 1; 1 mL/min; using a CHIRALCEL OD-H column (0.46 cmφ × 25 cm)): 9.3 min (minor) and 10.4 min (major).

2-(3-bromophenyl)-2-fluoropropan-1-ol (5g, 93% *ee*)

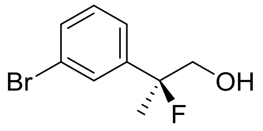

The reaction was stirred for 48 h at 0 °C. The crude mixture was purified by silica gel column chromatography (hexane : diethyl ether = 2:1) to give 77% yield of **5g** (colorless oil). ^{1}H NMR (400 MHz, $CDCl_3$): δ 7.53 (s, 1H), 7.48–7.41 (m, 1H), 7.32–7.20 (m, 2H), 3.87–3.70 (m, 2H), 1.92 (bs, 1H), 1.67 (d, J = 22.6 Hz, 3H); ^{13}C NMR (100 MHz, $CDCl_3$): δ 143.8 (d, J = 22.0 Hz), 130.9, 130.0, 127.7 (d, J = 10.5 Hz), 123.1 (d, J = 8.6 Hz), 122.7, 97.3 (d, J = 174.4 Hz), 69.3 (d, J = 24.9 Hz), 23.2 (d, J = 24.0 Hz); ^{19}F NMR (376 MHz, $CDCl_3$): δ –157.8 (m); $[\alpha]_D^{20}$ +12.3 (c = 0.32, $CHCl_3$); HRMS (DART): Anal. For $C_9H_{10}BrFO^{+1}$ $[M+NH_4]^+$ Calcd.: 250.0245, Found: 250.0243. The enantiopurity was determined by HPLC (hexane : 2-propanol = 50 : 1; 1 mL/min; using a CHIRALPAK ID-3 column (0.46 cmφ × 25 cm)): 12.8 min (major) and 27.2 min (minor).

2-fluoro-2-(naphthalen-2-yl)propan-1-ol (5h, 92% *ee*)

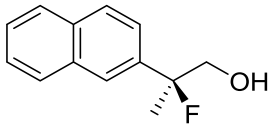

The reaction was stirred for 21 h at 0 °C. The crude mixture was purified by silica gel column chromatography (hexane : diethyl ether = 2:1) to give 98% yield of **5h** (white solid). ^{1}H NMR (400 MHz, $CDCl_3$): δ 7.86–7.82 (m, 4H), 7.52–7.47 (m, 2H), 7.42 (d, J = 8.8 Hz, 1H), 3.97–3.78 (m, 2H), 2.01 (bs, 1H), 1.77 (d, J = 22.6 Hz, 3H); ^{13}C NMR (100 MHz, $CDCl_3$): δ 138.8 (d, J = 21.1 Hz), 132.9, 132.7, 128.3, 128.2, 127.6, 126.4, 126.3, 123.5 (d, J = 10.5 Hz), 122.4 (d, J = 8.6 Hz), 98.0 (d, J = 172.5 Hz), 69.4 (d, J = 25.9 Hz), 23.2 (d, J = 24.9 Hz); ^{19}F NMR (376 MHz, $CDCl_3$): δ –157.2 (m); $[\alpha]_D^{20}$ +16.4 (c = 1.4, $CHCl_3$); HRMS (DART): Anal. For $C_{13}H_{13}F_1O_1^{+1}$ $[M+NH_4]^+$ Calcd.: 222.1294, Found: 222.1294. The enantiopurity was determined by HPLC (hexane : 2-propanol = 99 : 1; 2 mL/min; using a CHIRALPAK ID column (0.46 cmφ × 25 cm)): 15.2 min (major) and 25.0 min (minor).

2-fluoro-2-(5,6,7,8-tetrahydronaphthalen-2-yl)propan-1-ol (5i, 92% *ee*)

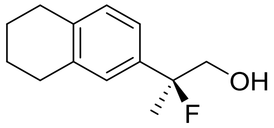

The reaction was stirred for 20 h at 0 °C. The crude mixture was purified by silica gel column chromatography (hexane : diethyl ether = 2:1) to give 95% yield of **5i** (white solid). ^{1}H NMR

(400 MHz, $CDCl_3$): δ 7.10–7.05 (m, 3H), 3.88–3.69 (m, 2H), 2.91–2.76 (m, 4H), 1.82–1.79 (m, 5H), 1.67 (d, J = 22.6 Hz, 3H); ^{13}C NMR (100 MHz, $CDCl_3$): δ 138.6 (d, J = 21.1 Hz), 137.2, 136.9, 129.2, 125.2 (d, J = 9.6 Hz), 121.5 (d, J = 8.6 Hz), 97.8 (d, J = 171.6 Hz), 69.6 (d, J = 24.9 Hz), 29.5, 29.0, 23.3, 23.1; ^{19}F NMR (376 MHz, $CDCl_3$): δ –157.2 (m); $[\alpha]_D^{20}$ +17.3 (c = 0.63, $CHCl_3$); HRMS (DART): Anal. For $C_{13}H_{17}F_1O_1^{+1}$ $[M+NH_4]^+$ Calcd.: 226.1607, Found: 226.1608. The enantiopurity was determined by HPLC (hexane : 2-propanol = 50 : 1; 1 mL/min; using a CHIRALPAK IC-3 column (0.46 cmφ × 25 cm)): 18.1 min (major) and 21.3 min (minor).

2-fluoro-2-(2-fluoro-[1,1'-biphenyl]-4-yl)propan-1-ol (5j, 92% *ee*)

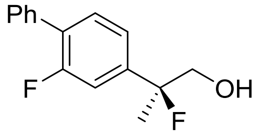

The reaction was stirred for 24 h at 0 °C. The crude mixture was purified by silica gel column chromatography (hexane : diethyl ether = 2:1) to give 98% yield of **5j** (white solid). ^{1}H NMR (500 MHz, $CDCl_3$): δ 7.56–7.53 (m, 2H), 7.48–7.41 (m, 3H), 7.39–7.36 (m, 1H), 7.22–7.16 (m, 2H), 3.92–3.76 (m, 2H), 1.97–1.94 (m, 1H), 1.72 (d, J = 22.6 Hz, 3H); ^{13}C NMR (126 MHz, $CDCl_3$): δ 159.6 (d, J = 248.3 Hz), 143.0 (dd, J = 22.8, 7.2 Hz), 135.2, 130.8 (d, J = 3.6 Hz), 128.9 (d, J = 3.6 Hz), 128.5, 127.8, 120.4 (dd, J = 9.6, 3.6 Hz), 112.8 (d, J = 10.8 Hz), 112.6 (d, J = 9.6 Hz), 97.3 (d, J = 172.7 Hz), 69.3 (d, J = 25.2 Hz), 23.2 (d, J = 25.2 Hz); ^{19}F NMR (470 MHz, $CDCl_3$): δ –117.7 (m), –157.3 (m); $[\alpha]_D^{21}$ +20.5 (c = 1.1, $CHCl_3$); HRMS (DART): Anal. For $C_{15}H_{14}F_2O_1^{+1}$ $[M+NH_4]^+$ Calcd.: 266.1357, Found: 266.1354. The enantiopurity was determined after conversion into the corresponding benzoate **18j**.

2-fluoro-2-(2-fluoro-[1,1'-biphenyl]-4-yl)propyl benzoate (18j, 92% *ee*)

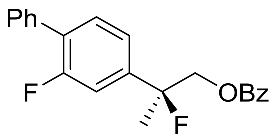

According to the general procedure, **5j** was converted into **18j**, the crude mixture was purified by flash column chromatography on silica gel (hexane : ethylacetate = 20:1) to afford the desired benzoate **18j** in 97 % yield (white solid). ^{1}H NMR (400 MHz, $CDCl_3$): δ 8.03 (d, J = 7.3 Hz, 2H), 7.59–7.54 (m, 3H), 7.50–7.42 (m, 5H), 7.40–7.37 (m, 1H), 7.30–7.26 (m, 2H), 4.66–4.53 (m, 2H), 1.83 (d, J = 22.3 Hz, 3H); ^{13}C NMR (100 MHz, $CDCl_3$): δ 166.0, 159.6 (d, J = 248.2 Hz), 142.6 (dd, J = 23.0, 7.7 Hz), 135.1, 133.3, 130.8 (d, J = 3.8 Hz), 129.7, 129.5, 129.0 (d, J = 2.9 Hz), 128.7 (d, J = 13.3 Hz), 128.5 (d, J = 3.8 Hz), 127.9, 120.5 (dd, J = 8.6, 2.9 Hz), 112.9 (d, J = 10.5 Hz), 112.7 (d, J = 9.6 Hz), 95.1 (d, J = 177.3 Hz), 69.4 (d, J = 24.9 Hz), 23.6

(d, J = 24.9 Hz); ^{19}F NMR (376 MHz, $CDCl_3$): δ –117.6 (m), –153.3 (m); $[\alpha]_D^{20}$ +23.8 (c = 0.74, $CHCl_3$); HRMS (DART): Anal. For $C_{22}H_{18}F_2O_2^{+1}$ $[M+NH_4]^+$ Calcd.: 370.1619, Found: 370.1618. The enantiopurity was determined by HPLC (hexane : 2-propanol = 100 : 1; 0.5 mL/min; using a CHIRALPAK IB-3 column (0.46 cmφ × 25 cm)): 18.2 min (minor) and 21.7 min (major).

1-fluoro-1,2,3,4-tetrahydronaphthalene-1-carbaldehyde (4k, 95% *ee*)

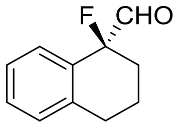

After completion of fluorination (stirred for 2 h at room temperature), the reaction mixture was added saturated aq.$NaHCO_3$ at 0 °C. The mixture was extracted with Et_2O, and the organic later was dried over Na_2SO_4, concentrated and purified by silica gel column chromatography (pantane : diethyl ether = 10 : 0 – 10 : 1) to afford 90% yield of **4k** (colorless oil; including small amount of impurities). ^{1}H NMR (400 MHz, $CDCl_3$): δ 9.80 (d, J = 6.1 Hz, 1H), 7.34–7.20 (m, 4H), 2.92–2.74 (m, 2H), 2.28–2.09 (m, 2H), 2.05–1.90 (m, 2H); ^{13}C NMR (100 MHz, $CDCl_3$): δ 197.9 (d, J = 38.3 Hz), 138.9 (d, J = 3.8 Hz), 130.3 (d, J = 21.1 Hz), 129.7 (d, J = 3.8 Hz), 129.6, 128.6 (d, J = 3.8 Hz), 126.7 (d, J = 1.9 Hz), 95.5 (d, J = 181.2 Hz), 29.5 (d, J = 21.1 Hz), 28.9, 18.5 (d, J = 2.9 Hz); ^{19}F NMR (376 MHz, $CDCl_3$): δ –142.4 (t, J = 23.1 Hz); $[\alpha]_D^{22}$ –18.5 (c = 0.49, $CHCl_3$); HRMS (DART): Anal. For $C_{11}H_{15}F_1N_1O_1^{+1}$ $[M+NH_4]^+$ Calcd.: 196.1138, Found 196.1131; The enantiopurity was determined by GC (100–150 °C, 5 °C/min; using a β-DEX 120 column): 21.5 min (minor) and 21.8 min (major).

2-fluoro-2-phenylbutan-1-ol (5l, 84% *ee*)

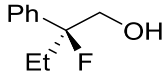

The reaction was stirred for 12 h at room temperature. The crude mixture was purified by silica gel column chromatography (hexane : diethyl ether = 2:1) to give 93% yield of **5l** (colorless oil). ^{1}H NMR (400 MHz, $CDCl_3$): δ 7.40–7.29 (m, 5H), 3.90–3.78 (m, 2H), 2.21–2.09 (m, 1H), 1.98–1.80 (m, 2H), 0.81 (t, J = 7.4 Hz, 3H); ^{13}C NMR (100 MHz, $CDCl_3$): δ 139.7 (d, J = 21.1 Hz), 128.3, 127.6, 124.8 (d, J = 9.6 Hz), 100.3 (d, J = 175.4 Hz), 68.7 (d, J = 24.0 Hz), 28.8 (d, J = 23.0 Hz), 7.1 (d, J = 5.8 Hz); ^{19}F NMR (376 MHz, $CDCl_3$): δ –170.6 (m); $[\alpha]_D^{23}$ +2.90 (c = 1.8, $CHCl_3$); HRMS (DART): Anal. For $C_{10}H_{13}F_1O_1^{+1}$ $[M+NH_4]^+$ Calcd.: 186.1294, Found: 186.1294. The enantiopurity was determined after conversion into the corresponding benzoate **18l**.

2-fluoro-2-phenylbutyl benzoate (18l, 84% *ee*)

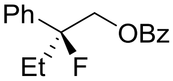

According to the general procedure, **5l** was converted into **18l**. The crude mixture was purified by flash column chromatography on silica gel (hexane : ethylacetate = 20:1) to afford the desired benzoate **18l** in 92 % yield (white solid). ^{1}H NMR (500 MHz, $CDCl_3$): δ 7.97 (d, *J* = 8.2 Hz, 2H), 7.57–7.53 (m, 1H), 7.43–7.37 (m, 6H), 7.35–7.30 (m, 1H), 4.68–4.53 (m, 2H), 2.30–2.18 (m, 1H), 2.11–1.94 (m, 1H), 0.86 (t, *J* = 7.5 Hz, 3H); ^{13}C NMR (100 MHz, $CDCl_3$): δ 166.1, 139.4 (d, *J* = 22.0 Hz), 133.1, 129.7, 129.7, 128.4, 127.7, 124.9, 124.8, 98.1 (d, *J* = 179.2 Hz), 69.1 (d, *J* = 24.9 Hz), 29.3 (d, *J* = 24.0 Hz), 7.1 (d, *J* = 4.8Hz); ^{19}F NMR (376 MHz, $CDCl_3$): δ –167.6 (m); $[\alpha]_D^{20}$ +8.90 (c = 0.61, $CHCl_3$); HRMS (DART): Anal. For $C_{17}H_{17}F_1O_2^{+1}$ $[M+NH_4]^+$ Calcd.: 290.1556, Found: 290.1558. The enantiopurity was determined by HPLC (hexane : 2-propanol = 100 : 1; 0.5 mL/min; using a CHIRALCEL OJ-H column (0.46 cmφ × 25 cm)): 22.5 min (minor) and 25.8 min (major).

2-fluoro-2,3-diphenylpropan-1-ol (5m, 84% *ee*)

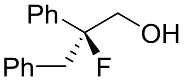

The reaction was stirred for 12 h at room temperature. The crude mixture was purified by silica gel column chromatography (hexane : diethyl ether = 2:1) to give 99% yield of **5m** (white solid). ^{1}H NMR (400 MHz, $CDCl_3$): δ 7.34–7.16 (m, 8H), 7.01–6.99 (m, 2H), 3.99–3.84 (m, 2H), 3.35 (dd, *J* = 17.7, 14.0 Hz, 1H), 3.22 (dd, *J* = 26.1, 14.2 Hz, 1H), 1.86 (t, *J* = 6.6 Hz, 1H); ^{13}C NMR (100 MHz, $CDCl_3$): δ 139.6 (d, *J* = 21.1 Hz), 134.9 (d, *J* = 3.8 Hz), 130.5, 128.2, 127.9, 127.7, 126.6, 124.9, 124.8, 99.3 (d, *J* = 177.3 Hz), 67.5 (d, *J* = 24.0 Hz), 43.1 (d, *J* = 23.0 Hz); ^{19}F NMR (376 MHz, $CDCl_3$): δ –165.5 (m); $[\alpha]_D^{20}$ –35.1 (c = 1.1, $CHCl_3$); HRMS (DART): Anal. For $C_{15}H_{15}F_1O_1^{+1}$ $[M+NH_4]^+$ Calcd.: 248.1451, Found: 248.1453. The enantiopurity was determined after conversion into the corresponding benzoate **18m.**

2-fluoro-2,3-diphenylpropyl benzoate (18m, 84% *ee*)

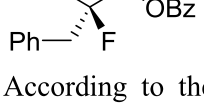

According to the general procedure, **5m** was converted into **18m**. The crude mixture was purified by flash column chromatography on silica gel (hexane : ethylacetate = 20:1) to afford the desired benzoate **18m** in 95 % yield (white solid). ^{1}H NMR (400 MHz, $CDCl_3$): δ 7.95 (d, *J* = 8.2 Hz, 2H), 7.56–7.52 (m, 1H), 7.42–7.39 (m, 2H), 7.35–7.26 (m, 5H), 7.22–7.16 (m, 3H), 7.06–7.04 (m, 2H), 4.72–4.59 (m, 2H), 3.44 (dd, *J* = 19.7, 14.2 Hz, 1H), 3.33 (dd, *J* = 25.2, 14.2

Hz, 1H); ^{13}C NMR (100 MHz, $CDCl_3$): δ 166.0, 139.4 (d, J = 21.1 Hz), 134.5 (d, J = 1.9 Hz), 133.1, 130.5, 129.6, 128.4, 128.2, 128.0 127.9, 126.8, 124.9, 124.8, 97.2 (d, J = 182.1 Hz), 68.5 (d, J = 24.9 Hz), 43.8 (d, J = 23.0 Hz); ^{19}F NMR (376 MHz, $CDCl_3$): δ –162.3 (m); $[\alpha]_D^{20}$ −16.5 (c = 1.3, $CHCl_3$); HRMS (DART): Anal. For $C_{22}H_{19}F_1O_2^{+1}$ $[M+NH_4]^+$ Calcd.: 352.1713, Found: 352.1714. The enantiopurity was determined by HPLC (hexane : 2-propanol = 100 : 1; 0.7 mL/min; using a CHIRALPAK ID column (0.46 cmφ × 25 cm)): 15.4 min (minor) and 17.5 min (major).

2-([1,1'-biphenyl]-4-yl)-2-fluoro-3-phenylpropan-1-ol (5n, 89% *ee*)

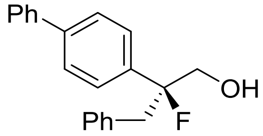

The reaction was stirred for 20 h at 0 °C using 20 mol% of **1b**. The crude mixture was purified by silica gel column chromatography (hexane : dethyl ether = 2:1) to give 91% yield of **5n** (89% *ee*) with a trace amount of impurity. Subsequent recrystallization from dichloromethane/hexane gave pure product with 93% *ee* (white solid). ^{1}H NMR (400 MHz, $CDCl_3$): δ 7.56–7.50 (m, 4H), 7.42–7.38 (m, 2H), 7.33–7.29 (m, 1H), 7.26 (d, J = 10.4 Hz, 2H), 7.17–7.16 (m, 3H), 7.03–7.01 (m, 2H), 3.95–3 .83 (m, 2H), 3.35 (dd, J = 18.0, 14.0 Hz, 1H), 3.22 (dd, J = 25.8, 14.2 Hz), 2.15 (bs, 1H); ^{13}C NMR (100 MHz, $CDCl_3$): δ 140.3 (d, J = 4.7 Hz), 138.7 (d, J = 21.9 Hz), 134.9 (d, J = 2.9 Hz), 130.5, 128.7, 127.9, 127.4, 127.0,. 126.8, 126.6, 125.4, 125.3, 99.2 (d, J = 176.4 Hz), 67.3 (d, J = 23.8 Hz), 43.0 (d, J = 23.9 Hz); ^{19}F NMR (376 MHz, $CDCl_3$): δ –164.9 (m); $[\alpha]_D^{20}$ −51.9 (c = 2.0, $CHCl_3$); HRMS (DART): Anal. For $C_{21}H_{19}F_1O_1$ $[M+NH_4]^+$ Calcd.: 324.1764, Found: 324.1761. The enantiopurity was determined by HPLC (hexane : 2-propanol = 30: 1; 1 mL/min; using a CHIRALPAK ID-3 column (0.46 cmφ × 25 cm)): 21.1 min (minor) and 24.6 min (major).

2-cyclohexyl-2-fluoropropan-1-ol [5o, 83% *ee*]

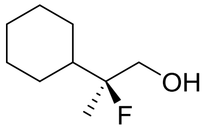

The reaction was carried out at 0 °C and stirred for 48 h with 30 mol% catalyst **1b** in the absence of 3,5-$(NO_2)_2C_6H_3CO_2H$. The crude mixture was purified by silica gel column chromatography (hexane : diethyl ether = 2:1) to give 24% yield of **5o** (colorless oil, mixture of **5o** and 2-cyclohexylpropan-1-ol). ^{1}H NMR (500 MHz, $CDCl_3$): δ 3.69 (dd, J = 21.4, 12.2 Hz, 1H), 3.57 (dd, J = 23.0, 11.8 Hz, 1H), 1.84–1.62 (m, 7H), 1.28–1.20 (m, 1H), 1.24 (d, 3H), 1.18–1.07 (m, 2H), 1.02–0.94 (m, 1H); ^{13}C NMR (126 MHz, $CDCl_3$): δ 99.8 (d, J = 167.9 Hz),

66.8 (d, J = 24.0 Hz), 42.9 (d, J = 21.6 Hz), 27.6 (d, J = 7.2 Hz), 26.4–26.3 (3C), 17.6 (d, J = 25.2 Hz); ^{19}F NMR (470 MHz, $CDCl_3$): δ –158.1; HRMS (DART): Anal. For $C_9H_{17}FO_1^{+1}$ $[M+NH_4]^+$ Calcd.: 178.1605, Found: 178.1607. The enantiopurity was determined by GC (100 °C–130 °C, 8 °C/min, then 60 min at 130 °C) using a β-DEX 120 column: 20.0 (major) and 22.4 (minor).

2-fluoro-3-(4-isopropylphenyl)-2-methylpropan-1-ol (5p, 14% *ee*)

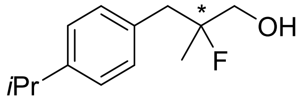

The reaction was stirred for 24 h at room temperature. The crude mixture was purified by silica gel column chromatography (hexane : diethyl ether = 2:1) to give 59% yield of **5p** (colorless oil). ^{1}H NMR (400 MHz, $CDCl_3$): δ 7.16 (s, 4H), 3.58 (dd, J = 19.5, 5.5 Hz, 2H), 3.01–2.82 (m, 3H), 1.88 (m, 1H), 1.30–1.23 (m, 9H); ^{13}C NMR (100 MHz, $CDCl_3$): δ 147.3, 133.2 (d, J = 5.8 Hz), 130.3, 126.3, 97.4 (d, J = 169.7 Hz), 67.5 (d, J = 24.0 Hz), 41.9 (d, J = 23.0 Hz), 33.7, 24.0, 20.9 (d, J = 24.0 Hz); ^{19}F NMR (376 MHz, $CDCl_3$): δ –154.7 (m); HRMS (DART): Anal. For $C_{13}H_{19}F_1O_1^{+1}$ $[M+NH_4]^+$ Calcd.: 210.1420, Found: 210.1419. The enantiopurity was determined after conversion into the corresponding benzoate **18p**.

2-fluoro-3-(4-isopropylphenyl)-2-methylpropyl benzoate (18p, 14% *ee*)

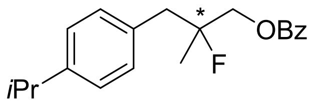

According to the general procedure, **5p** was converted into **18p**. The crude mixture was purified by silica gel column chromatography (hexane : ethyl acetate = 10 : 1) to afford 91% of **18p** (colorless oil). ^{1}H NMR (400 MHz, $CDCl_3$): δ 8.09 (d, J = 8.2 Hz, 2H), 7.59 (t, J = 7.9 Hz, 1H), 7.47 (t, J = 7.6 Hz, 2H), 7.16 (s, 4H), 4.35 (dd, J = 37.0, 11.9 Hz, 1H), 4.30 (dd, J = 37.2, 11.9 Hz,1H), 3.06 (d, J = 19.5 Hz, 2H), 2.88 (sept, J = 7.0, 1H), 1.41 (d, J = 21.5 Hz, 3H), 1.24 (d, J = 7.0 Hz, 6H); ^{13}C NMR (125 MHz, $CDCl_3$): δ 166.1, 147.5, 133.2, 132.6 (d, J = 4.8 Hz), 130.2, 129.8, 129.7, 128.5, 126.4, 95.0 (d, J = 175.1 Hz), 68.1 (d, J = 25.2 Hz), 42.8 (d, J = 22.8 Hz), 33.7, 24.0, 21.7 (d, J = 24.0 Hz); ^{19}F NMR (376 MHz, $CDCl_3$): δ –151.6; HRMS (DART): Anal. For $C_{20}H_{24}F_1O_2^{+1}$ $[M+H^+]$ Calcd.: 315.1760, Found: 315.1763; The enantiopurity was determined by HPLC (hexane : 2-propanol = 100 : 1; 0.5 mL/min; using a CHIRALCEL OJ-H column (0.46 cmφ × 25 cm)): 26.5 min (minor) and 27.9 min (major).

<u>**Derivatization of α-fluoroaldehydes (Scheme 3).**</u>

Horner–Wadsworth–Emmons reaction of α-fluoroaldehydes.

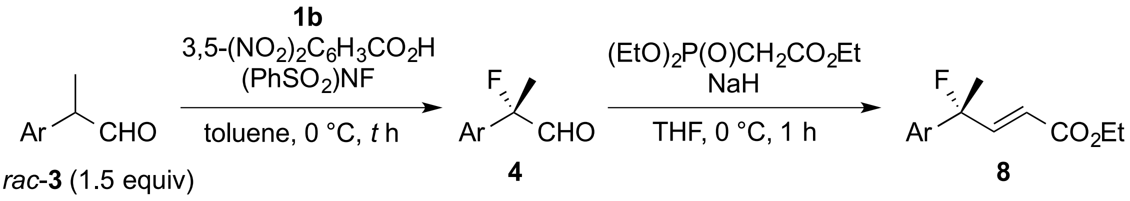

To a solution of catalyst **1b** (20 mg, 0.026 mmol, 10 mol%) in toluene (0.54 mL) was added 3,5-dinitrobenzoic acid (5.5 mg, 0.026 mmol, 10 mol%), aldehydes **3** (0.39 mmol, 1.5 equiv), and NFSI (0.26 mmol, 82 mg, 1 equiv) at 0 ºC. The mixture was stirred for 24 h at 0 °C, then poured into aq. $NaHCO_3$, and extracted by Et_2O. The organic layer was dried over Na_2SO_4 and concentrated under the reduced pressure gave **4** as the crude product. To a solution of $(EtO)_2P(O)CH_2CO_2Et$ (1.35 mmol) in THF (0.7 mL) was added NaH (60%, 1.35 mmol) at 0 °C. After the mixture was stirred for 0.5 h at 0 °C, a solution of **4** in THF (1.0 mL) was added to the mixture, then stirred for another 1 h. The mixture was quenched with saturated aq.NH_4Cl and extracted with Et_2O. The organic layer was washed with brine, dried over Na_2SO_4, and concentrated. The crude mixture was purified by flash column chromatography on silica gel to afford **8**.

ethyl (*E*)-4-(4-bromophenyl)-4-fluoropent-2-enoate (8b, 92% *ee*)

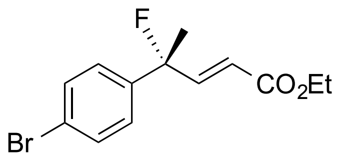

The crude mixture was purified by flash column chromatography on silica gel (hexane : ethyl acetate = 30 : 1) to afford 80% yield of **8b** (colorless oil). ^{1}H NMR (500 MHz, $CDCl_3$): δ 7.51 (d, *J* = 8.0 Hz, 2H), 7.26 (d, *J* = 8.4 Hz, 2H), 7.05 (dd, *J* = 18.7, 15.7 Hz, 1H), 6.09 (d, *J* = 15.7 Hz, 1H), 4.20 (q, *J* = 7.13 Hz, 2H), 1.80 (d, *J* = 21.8 Hz, 3H), 1.29, (t, *J* = 7.3 Hz, 3H); ^{13}C NMR (125 MHz, $CDCl_3$): δ 166.0, 148.0 (d, *J* = 22.8 Hz), 140.5 (d, *J* = 22.8 Hz), 131.7, 126.4 (d, *J* = 8.4 Hz), 122.3, 119.8 (d, *J* = 10.8 Hz), 94.8 (d, *J* = 176.3 Hz), 60.8, 26.5 (d, *J* = 25.2 Hz), 14.2; ^{19}F NMR (470 MHz, $CDCl_3$): δ –146.2 (m); $[\alpha]_D^{29}$ +17.1 (c = 1.2, $CHCl_3$); HRMS (DART): Anal. For $C_{13}H_{18}Br_1F_1N_1O_2^{+1}$ $[M+NH_4]^+$ Calcd.: 318.0505, Found: 318.0504.

ethyl (*E*)-4-fluoro-4-(naphthalen-2-yl)pent-2-enoate (8h, 92% *ee*)

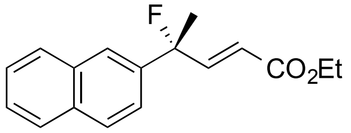

The crude mixture was purified by flash column chromatography on silica gel (hexane : ethyl acetate = 30 : 1) to afford 80% yield of **8h** (white solid). ^{1}H NMR (500 MHz, $CDCl_3$): δ 7.85–7.81 (m, 4H), 7.51–7.46 (m, 3H), 7.20 (dd, *J* = 18.7, 15.7 Hz, 1H), 6.16 (dd, *J* = 15.6, 0.8 Hz, 1H), 4.20 (q, *J* = 7.3 Hz, 2H), 1.92 (d, *J* = 22.2 Hz, 3H), 1.27 (t, *J* = 6.9 Hz, 3H); ^{13}C NMR (125 MHz, $CDCl_3$): δ 166.2, 148.7 (d, *J* = 24.0 Hz), 138.7 (d, *J* = 22.8 Hz), 132.9 (d, *J* = 10.8 Hz), 128.5, 128.3, 127.6, 126.5, 123.4 (d, *J* = 9.6 Hz), 122.6 (d, *J* = 6.0 Hz), 119.6 (d, *J* = 10.8 Hz), 95.3 (d, *J* = 175.1 Hz), 60.7, 26.5 (d, *J* = 25.2 Hz), 14.1; ^{19}F NMR (470 MHz, $CDCl_3$): δ –145.5 (m); $[\alpha]_D^{29}$ +24.5 (c = 1.1, $CHCl_3$); HRMS (DART): Anal. For $C_{17}H_{21}F_1N_1O^{+1}$ $[M+NH_4]^+$ Calcd.: 290.1556, Found: 290.1554.

Synthesis of fluorinated analogue of flurbiprofen

2-fluoro-2-(2-fluoro-[1,1'-biphenyl]-4-yl)propanoic acid (9, 92% *ee*)

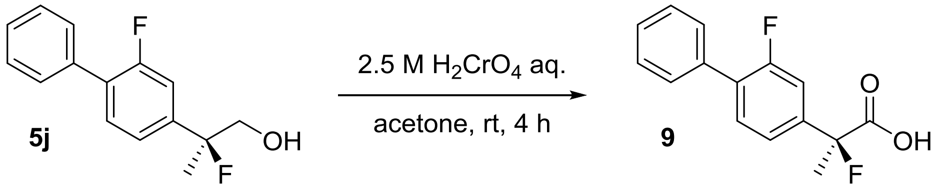

A solution of **5j** (0.106 mmol) in acetone (1.06 mL) was added to 2.5 M aq.H_2CrO_4 (3 mmol, 128 μL) at 0 °C. After the mixture was stirred for 4 h at room temperature, 2-propanol was added to this mixture. The mixture was filtered, extracted by CH_2Cl_2, and the organic layer was washed by 1.2N HCl twice and brine, dried over Na_2SO_4 and concentrated. The crude mixture was purified by silica gel column chromatography (hexane : ethyl acetate = 4 : 1 – 1 : 4) to afford 69% yield of **9** (white solid; including small amount of impurities). ^{1}H NMR (500 MHz, CD_3OD): δ 7.54–7.49 (m, 3H), 7.45–7.40 (m, 3H), 7.39–7.34 (m, 2H), 1.93 (d, *J* = 22.2 Hz, 3H); ^{13}C NMR (126 MHz, CD_3OD): δ 173.7 (d, *J* = 27.6 Hz), 160.8 (d, *J* = 247.1 Hz), 142.7 (d, *J* = 7.2 Hz), 142.5 (d, *J* = 7.2 Hz), 136.4, 132.0 (d, *J* = 2.4 Hz), 130.5 (d, *J* = 13.2 Hz), 130.0 (d, *J* = 2.4 Hz), 129.0, 122.1 (d, *J* = 3.6 Hz), 122.0 (d, *J* = 3.6 Hz), 113.9 (d, *J* = 9.2 Hz), 113.7 (d, *J* = 9.2 Hz), 95.1 (d, *J* = 184.7 Hz), 25.0 (d, 24.0 Hz); ^{19}F NMR (470 MHz, CD_3OD): δ –116.1, –147.9 (q, *J* = 22.0 Hz); $[\alpha]_D^{27}$ +28.6 (c = 0.84, $CHCl_3$); HRMS (DART): Anal. For $C_{15}H_{16}F_2N_1O_2^{+1}$ $[M+NH_4]^+$ Calcd.: 280.1149, Found 280.1143.

Synthesis of α-hydroxyacetals 10 (Table 3).

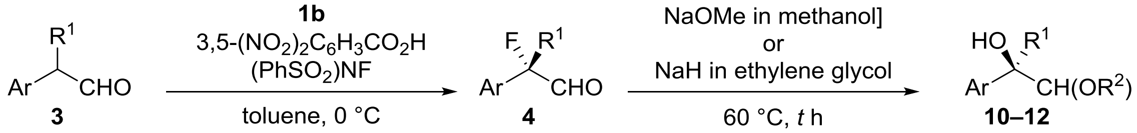

General procedure: Enantioselective fluorination of **3** was carried out according to the procedure described in page S6. After completion of the reaction, MeOH (2.64 mL)/NaOMe (1.32 mmol, 5 equiv.) or ethylene glycol (2.64 mL)/NaH (1.32 mmol, 5 equiv) were added at 0 °C. The mixture was stirred at room temperature, then diluted by adding sat.$NaHCO_3$ aq., and extracted with Et_2O. The organic layer was dried over Na_2SO_4 and concentrated under reduced pressure. The crude product was purified by silica gel chromatography to give α-hydroxylacetals **10–12**.

(*R*)-1,1-dimethoxy-2-phenylpropan-2-ol [(*R*)-10a, 94% *ee*]

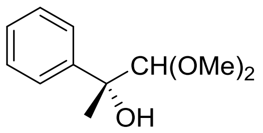

The reaction was stirred for 10 h. The crude mixture was purified by silica gel column chromatography (hexane : ethyl acetate = 4 : 1) to give 66% yield of (*R*)-**10a** (pale yellow oil). ^{1}H NMR (400 MHz, C_6D_6): δ 7.70–7.67 (m, 2H), 7.26–7.22 (m, 2H), 7.14–7.10 (m, 1H), 3.98 (s, 1H), 3.04 (s, 3H), 2.95 (s, 3H), 2.50 (s, 1H), 1.59 (s, 3H); ^{13}C NMR (100 MHz, C_6D_6): δ 145.1, 128.0, 127.1, 126.6, 111.1, 76.1, 57.4, 57.3, 23.8; $[\alpha]_D^{25}$ –7.6 (c = 1.00, $CHCl_3$); HRMS (DART): Anal. For $C_{11}H_{20}N_1O_3^{+1}$ [M+NH_4^+] Calcd.: 214.1443, Found: 214.1441; The enantiopurity was determined after conversion into methyl ether (*R*)-**19a**.

Methylation of 10.

General procedure: To a suspension of NaH (0.408 mmol, 2 equiv.) in DMF (1.0 mL), α-hydroxyacetal **10** (0.204 mmol) was added, and the mixture was stirred at 0 °C for 30 min. MeI (0.408 mmol, 2 equiv.) was added to the mixture, and stirred for 60 min at 0 °C. The reaction was quenched by adding sat. NH_4Cl aq. and extracted with Et_2O. The organic layer was dried over Na_2SO_4 and concentrated under reduced pressure. The crude product was purified by silica gel chromatography to afford **19**.

(*R*)-(1,1,2-trimethoxypropan-2-yl)benzene [(*R*)-19a, 94% *ee*]

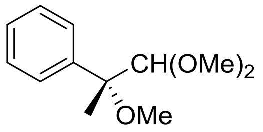

According to the general procedure, reaction was carried out with 0.204 mmol of (*R*)-**10a**. The crude mixture was purified by silica gel column chromatography (hexane : ethyl acetate = 15 : 1) to afford 83% yield of (*R*)-**19a** (colorless oil). ^{1}H NMR (400 MHz, $CDCl_3$): δ 7.44–7.42 (m, 2H), 7.38–7.34 (m, 2H), 7.30–7.26 (m, 1H), 4.13 (s, 1H), 3.48 (s, 3H), 3.10 (s, 3H), 3.08 (s, 3H), 1.56 (s, 3H); ^{13}C NMR (100 MHz, $CDCl_3$): δ 141.2, 127.9, 127.5, 127.2, 111.0, 81.7, 58.2, 57.3, 50.2, 15.5; $[\alpha]_D^{23}$ –53.2 (c = 1.00, $CHCl_3$); HRMS (DART): Anal. For $C_{12}H_{22}N_1O_3^{+1}$ [M+NH_4^+] Calcd.: 228.1600, Found: 228.1600; The enantiopurity was determined by HPLC (hexane : 2-propanol = 300 : 1, 1.0 mL/min, 220 nm) using a CHIRALPAK IC-3 column (0.46 cmφ x 25 cm): 10.6 min (major) and 12.9 min (minor).

2-(4-bromophenyl)-1,1-dimethoxypropan-2-ol (10b, 92% *ee*)

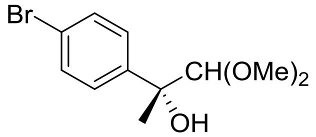

The reaction was stirred for 12 h. The crude mixture was purified by silica gel column chromatography (hexane : ethyl acetate = 4 : 1) to give 70% yield of **10b** (pale yellow oil). ^{1}H NMR (500 MHz, C_6D_6): δ 7.34 (s, 4H), 3.81 (s, 1H), 2.99 (s, 3H), 2.89 (s, 3H), 2.38 (s, 1H), 1.47 (s, 3H); ^{13}C NMR (100 MHz, C_6D_6): δ 144.0, 131.1, 128.5, 121.3, 110.7, 75.7, 57.4, 57.3, 23.7; $[\alpha]_D^{21}$ –3.2 (c = 1.02, $CHCl_3$); HRMS (DART): Anal. For $C_{11}H_{19}Br_1N_1O_3^{+1}$ [M+NH_4^+] Calcd.: 292.0548, Found: 292.0548; The enantiopurity was determined after conversion into **19b**.

1-bromo-4-(1,1,2-trimethoxypropan-2-yl)benzene (19b, 92% *ee*)

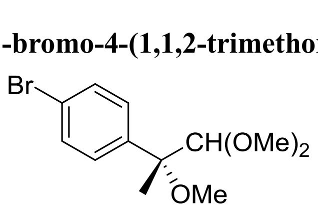

According to the general procedure, reaction was carried out with 0.145 mmol of **10b**. The crude mixture was purified by silica gel column chromatography (hexane : ethyl acetate = 15 : 1) to give 76% yield of **19b** (colorless oil). ^{1}H NMR (400 MHz, $CDCl_3$): δ 7.49–7.47 (m, 2H), 7.31–7.28 (m, 2H), 4.08 (s, 1H), 3.48 (s, 3H), 3.16 (s, 3H), 3.09 (s, 3H), 1.56 (s, 3H); ^{13}C NMR (100 MHz, $CDCl_3$): δ 140.4, 131.0, 129.4, 121.4, 110.6, 81.6, 58.2, 57.6, 50.2, 15.9; $[\alpha]_D^{23}$ –41.0 (c = 1.01, $CHCl_3$); HRMS (DART): Anal. For $C_{12}H_{21}N_1O_3^{+1}$ [M+NH_4^+] Calcd.: 306.0705,

Found: 306.0705; The enantiopurity was determined by HPLC (hexane : 2-propanol = 300 : 1, 1.0 mL/min, 220 nm) using a CHIRALPAK IC-3 column (0.46 cmφ x 25 cm): 7.7 min (major) and 9.0 min (minor).

2-(4-fluorophenyl)-1,1-dimethoxypropan-2-ol [10c, 93% *ee*]

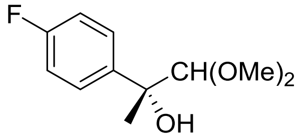

The reaction was stirred for 10 h. The crude mixture was purified by silica gel column chromatography (hexane : ethyl acetate = 4 : 1–2 : 1) to give 64% yield of **10c** (colorless oil). ^{1}H NMR (500 MHz, C_6D_6): δ 7.50–7.46 (m, 2H), 6.90–6.86 (m, 2H), 3.85 (s, 1H), 3.03 (s, 3H), 2.91 (s, 3H), 2.42 (s, 1H), 1.52 (s, 3H); ^{13}C NMR (126 MHz, $CDCl_3$): δ 162.4 (d, J = 244.7 Hz), 140.6, 128.4 (d, J = 7.2 Hz), 114.6 (d, J = 21.6 Hz), 111.0, 75.7, 57.5, 57.3, 23.7; ^{19}F NMR (470 MHz, $CDCl_3$): δ –115.8; $[\alpha]_D^{25}$ –7.6 (c = 0.33, $CHCl_3$); HRMS (DART): Anal. For $C_{11}H_{15}FO_3^{+1}$ $[M+NH_4]^+$ Calcd.: 232.1347, Found: 232.1349. The enantiopurity was determined by HPLC (hexane : 2-propanol = 100 : 1; 1.0 mL/min; using a CHIRALPAK IA-3 column (0.46 cmφ × 25 cm)): 12.2 min (minor) and 14.2 min (major).

1,1-dimethoxy-2-(p-tolyl)propan-2-ol [10d, 93% *ee*]

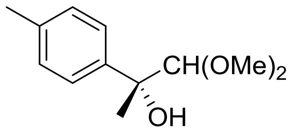

The reaction was stirred for 10 h. The crude mixture was purified by silica gel column chromatography (hexane : ethyl acetate = 4 : 1–2 : 1) to give 50% yield of **10d** (colorless oil). ^{1}H NMR (500 MHz, $CDCl_3$): δ 7.62 (d, J = 8.4 Hz, 2H), 7.08 (J = 8.0 Hz, 2H), 4.01 (s, 1H), 3.06 (s, 3H), 2.98 (s, 3H), 2.50 (s, 1H), 2.14 (s, 3H), 1.65 (s, 3H); ^{13}C NMR (126 MHz, $CDCl_3$): δ 142.2, 136.3, 128.7, 126.6, 111.2, 75.9, 57.4, 57.2, 23.8, 21.0; $[\alpha]_D^{25}$ –8.5 (c = 0.90, $CHCl_3$); HRMS (DART): Anal. For $C_{12}H_{18}O_3^{+1}$ $[M+NH_4]^+$ Calcd.: 228.1597, Found: 228.1600. The enantiopurity was determined by HPLC (hexane : 2-propanol = 100 : 1; 1.0 mL/min; using a CHIRALPAK IA-3 column (0.46 cmφ × 25 cm)): 17.0 min (minor) and 19.8 min (major).

1,1-dimethoxy-2-(naphthalen-2-yl)propan-2-ol (10h, 92% *ee*)

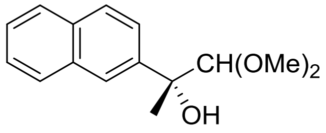

The reaction was stirred for 12 h. The crude mixture was purified by silica gel column

chromatography (hexane : ethyl acetate = 4 : 1) to give 82% yield of **10h** (pale yellow oil). ^{1}H NMR (400 MHz, C_6D_6): δ 8.25 (s, 1H), 7.81–7.65 (m, 4H), 7.28–7.25 (m, 2H), 4.08 (s, 1H), 3.06 (s, 3H), 2.94 (s, 3H), 2.64 (s, 1H), 1.72 (s, 3H); ^{13}C NMR (100 MHz, C_6D_6): δ 142.6, 133.7, 133.1, 128.6, 127.8, 127.6, 126.1, 125.9, 125.4, 125.2, 111.0, 76.2, 57.4, 57.2, 23.9; $[\alpha]_D^{23}$ –1.0 (c = 1.00, $CHCl_3$); HRMS (DART): Anal. For $C_{15}H_{22}N_1O_3^{+1}$ [M+NH$_4^+$] Calcd.: 264.1600, Found: 264.1603; The enantiopurity was determined after conversion into **19h**.

2-(1,1,2-trimethoxypropan-2-yl)naphthalene (19h, 92% *ee*)

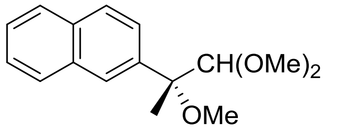

According to the general procedure, reaction was carried out with 0.162 mmol of **10h**. The crude mixture was purified by silica gel column chromatography (hexane : ethyl acetate = 10 : 1) to give 84% yield of **19h** (white solid). ^{1}H NMR (400 MHz, $CDCl_3$): δ 7.88–7.86 (m, 4H), 7.62–7.59 (m, 1H), 7.50–7.46 (m, 2H), 4.25 (s, 1H), 3.52 (s, 3H), 3.13 (s, 3H), 3.07 (s, 3H), 1.71 (s, 3H); ^{13}C NMR (100 MHz, $CDCl_3$): δ 139.0, 133.0, 132.6, 128.2, 127.44, 127.40, 126.8, 125.9, 125.8, 125.5, 110.9, 81.9, 58.2, 57.5, 50.3, 15.7; $[\alpha]_D^{23}$ –55.2 (c = 1.00, $CHCl_3$); HRMS (DART): Anal. For $C_{16}H_{24}N_1O_3^{+1}$ [M+NH$_4^+$] Calcd.: 278.1756, Found: 278.1757; The enantiopurity was determined by HPLC (hexane : 2-propanol = 300 : 1, 1.0 mL/min, 254 nm) using a CHIRALPAK IC-3 column (0.46 cmφ x 25 cm): 14.1 min (major) and 17.6 min (minor).

1,1-dimethoxy-2-(5,6,7,8-tetrahydronaphthalen-2-yl)propan-2-ol [10i, 91% *ee*]

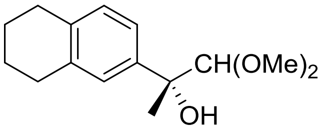

The reaction was stirred for 12 h. The crude mixture was purified by silica gel column chromatography (hexane : ethyl acetate = 4 : 1–2 : 1) to give 57% yield of **10i** (colorless oil). ^{1}H NMR (500 MHz, $CDCl_3$): δ 7.51 (s, 1H), 7.46 (dd, J = 7.8, 1.7 Hz, 1H), 7.02 (d, J = 8.0 Hz, 1H), 4.09 (s, 1H), 3.10 (s, 3H), 3.03 (s, 3H), 2.68–2.66 (m, 2H), 2.61–2.59 (m, 2H), 2.58 (s, 1H), 1.68 (s, 3H), 1.60–1.54 (m, 4H); ^{13}C NMR (126 MHz, $CDCl_3$): δ 142.3, 136.3, 135.5, 128.8, 127.1, 123.9, 111.1, 76.0, 57.3, 57.2, 26.9, 26.3, 24.0, 23.7, 23.6; $[\alpha]_D^{25}$ –4.2 (c = 0.98, $CHCl_3$); HRMS (DART): Anal. For $C_{15}H_{22}O_3^{+1}$ $[M+H]^+$ Calcd.: 251.1650, Found: 251.1647. The enantiopurity was determined by HPLC (hexane : 2-propanol = 50 : 1; 1.0 mL/min; using a CHIRALPAK IC-3 column (0.46 cmφ × 25 cm)): 25.3 min (minor) and 32.7 min (major).

2-(2-fluoro-[1,1'-biphenyl]-4-yl)-1,1-dimethoxypropan-2-ol [10j, 90% *ee*]

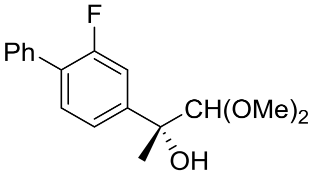

The reaction was stirred for 10 h. The crude mixture was purified by silica gel column chromatography (hexane : ethyl acetate = 4 : 1–2 : 1) to give 71% yield of **10j** (colorless oil). ^{1}H NMR (500 MHz, $CDCl_3$): δ 7.61 (dd, J = 12.6, 1.9 Hz, 1H), 7.55–7.52 (m, 2H), 7.44 (dd, J = 8.0, 1.9 Hz, 1H), 7.30 (t, J = 8.4 Hz, 1H), 7.21–7.18 (m, 2H), 7.12–7.09 (m, 1H), 3.95 (s, 1H), 3.06 (s, 3H), 2.98 (s, 3H), 2.54 (s, 1H), 1.56 (s, 3H); ^{13}C NMR (126 MHz, $CDCl_3$): δ 160.0 (d, J = 245.9 Hz), 147.0 (d, J = 7.2 Hz), 136.2, 130.3 (d, J = 3.6 Hz), 129.4 (d, J = 2.4 Hz), 128.7, 128.3, 127.9, 122.6 (d, J = 3.6 Hz), 114.7 (d, J = 25.2 Hz), 110.7, 75.8, 57.5, 57.4, 23.9; ^{19}F NMR (470 MHz, $CDCl_3$): δ –117.8 (J = 14.7 Hz); $[\alpha]_D^{24}$ –3.5 (c = 1.65, $CHCl_3$); HRMS (DART): Anal. For $C_{17}H_{19}FO_3^{+1}$ $[M+NH_4]^+$ Calcd.: 308.1664, Found: 308.1662. The enantiopurity was determined by HPLC (hexane : 2-propanol = 100 : 1; 1.0 mL/min; using a CHIRALPAK IA-3 column (0.46 cmφ × 25 cm)): 16.7 min (major) and 18.0 min (minor).

1-(dimethoxymethyl)-1,2,3,4-tetrahydronaphthalen-1-ol (10k, 94% *ee*)

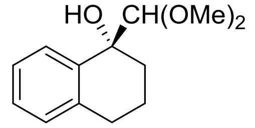

The reaction was carried out with 10 equiv. of NaOMe and stirred for 8 h at room temperature. The crude mixture was purified by silica gel column chromatography (hexane : ethyl acetate = 4 : 1) to give 55% yield of **10k** (pale yellow oil). ^{1}H NMR (400 MHz, C_6D_6): δ 7.70–7.68 (m, 1H), 7.16–7.13 (m, 1H), 7.10–7.06 (m, 1H), 6.97–6.95 (m, 1H), 4.30 (s, 1H), 3.19 (s, 3H), 2.91 (s, 3H), 2.60–2.56 (m, 2H), 2.46–2.37 (m, 2H), 1.94–1.85 (m, 2H), 1.78–1.69 (m, 1H); ^{13}C NMR (100 MHz, C_6D_6): δ 139.1, 138.9, 129.0, 127.9, 127.3, 126.0, 73.8, 57.6, 57.4, 32.5, 30.4, 19.8; $[\alpha]_D^{21}$ –3.7 (c = 1.00, $CHCl_3$); HRMS (DART): Anal. For $C_{13}H_{22}N_1O_3^{+1}$ $[M+NH_4^+]$ Calcd.: 240.1600, Found: 240.1597; The enantiopurity was determined by HPLC (hexane : 2-propanol = 100 : 1, 1.0 mL/min, 220 nm) using a CHIRALPAK IE-3 column (0.46 cmφ x 25 cm): 41.1 min (major) and 36.6 min (minor).

1,1-dimethoxy-2-phenylbutan-2-ol (10l, 82% *ee*)

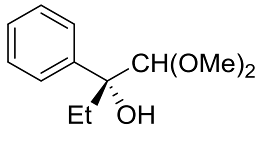

The reaction was stirred for 24 h under reflux condition. The crude mixture was purified by silica gel column chromatography (hexane : ethyl acetate = 6 : 1) to give 71% yield of **10l** (colorless oil). ^{1}H NMR (400 MHz, C_6D_6): δ 7.67–7.65 (m, 2H), 7.27–7.23 (m, 2H), 7.14–7.10 (m, 1H), 4.02 (s, 1H), 3.00 (s, 6H), 2.41 (s, 1H), 2.09–1.97 (m, 2H), 0.869 (t, 3H, J = 7.6 Hz); ^{13}C NMR (100 MHz, C_6D_6): δ 142.9, 128.0, 127.0, 126.8, 110.9, 78.7, 57.20, 57.15, 28.9, 7.4; $[\alpha]_D^{21}$ +12.2 (c = 0.99, $CHCl_3$); HRMS (DART): Anal. For $C_{12}H_{22}N_1O_3^{+1}$ [M+NH_4^+] Calcd.: 228.1600, Found: 228.1590. The enantiopurity was determined by HPLC (hexane : 2-propanol = 100 : 1, 1.0 mL/min, 220 nm) using a CHIRALPAK IC-3 column (0.46 cmφ x 25 cm): major isomer 12.3 min and minor isomer 11.4 min.

1,1-diethoxy-2-phenylpropan-2-ol [11, 79% *ee*]

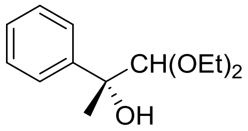

Fluorination was carried out at room temperature and stirred for 1.5 h. The reaction was stirred for 17 h in ethanol under reflux condition. The crude mixture was purified by silica gel column chromatography (hexane : ethyl acetate = 15 : 1–10 : 1) to give 43% yield of **11** (yellow oil, including ca. 10% of an inseparable by-product). ^{1}H NMR (500 MHz, $CDCl_3$): δ 7.75–7.72 (m, 2H), 7.26–7.23 (m, 2H), 7.15–7.11 (m, 1H), 4.19 (s, 1H), 3.47 (qd, J = 9.4, 7.1 Hz, 1H), 3.36 (qd, J = 9.2, 6.9 Hz, 1H), 3.13 (qd, J = 9.4, 7.1 Hz, 1H), 2.95 (qd, J = 9.2, 6.9 Hz, 1H), 2.68 (s, 1H), 1.68 (s, 3H), 0.94 (t, J = 6.9 Hz, 3H), 0.92 (t, J = 5.4 Hz, 3H); ^{13}C NMR (126 MHz, $CDCl_3$): δ 145.2, 127.9, 127.0, 126.7, 108.7, 75.9, 65.7, 65.4, 23.6, 15.4, 15.3; $[\alpha]_D^{25}$ –9.9 (c = 1.20, $CHCl_3$); HRMS (DART): Anal. For $C_{13}H_{20}O_3^{+1}$ $[M+NH_4]^+$ Calcd.: 242.1759, Found: 242.1756. The enantiopurity was determined by HPLC (hexane : 2-propanol = 100 : 1; 1.0 mL/min; using a CHIRALPAK IA-3 column (0.46 cmφ × 25 cm)): 6.5 min (minor) and 7.1 min (major).

1-(1,3-dioxolan-2-yl)-1-phenylethan-1-ol (12a, 94% *ee*)

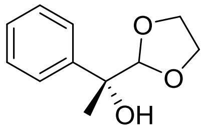

According to the typical procedure, reaction was carried out using ethyleneglycole and NaH instead of MeOH and NaOMe and stirred for 5 h. The crude mixture was purified by silica gel column chromatography (hexane : ethyl acetate = 4 : 1) to give 63% yield of **12a** (colorless oil). ^{1}H NMR (400 MHz, C_6D_6): δ 7.64–7.62 (m, 2H), 7.24–7.20 (m, 2H), 7.13–7.08 (m, 1H), 4.90 (s, 1H), 3.30–3.18 (m, 4H), 2.49 (bs, 1H), 1.55 (s, 3H); ^{13}C NMR (100 MHz, C_6D_6): δ 144.5, 128.1,

127.1, 126.3, 107.9, 75.0, 65.6, 65.3, 24.7; $[\alpha]_D^{19}$ –3.4 (c = 1.00, $CHCl_3$); HRMS (DART): Anal. For $C_{11}H_{18}N_1O_3^{+1}$ [M+NH_4^+] Calcd.: 212.1287, Found: 212.1284; The enantiopurity was determined by HPLC (hexane : 2-propanol = 100 : 1, 1.0 mL/min, 220 nm) using a CHIRALPAK IB-3 column (0.46 cmφ x 25 cm): 20.3 min (major) and 22.3 min (minor).

1-(1,3-dioxolan-2-yl)-1-(2-fluoro-[1,1'-biphenyl]-4-yl)ethan-1-ol [12j, 90% *ee*]

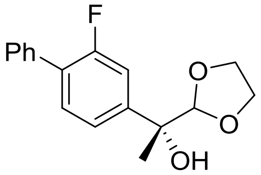

The reaction was stirred for 10 h. The crude mixture was purified by silica gel column chromatography (hexane : ethyl acetate = 2 : 1–1 : 1) to give 76% yield of **12j** (colorless oil). ^{1}H NMR (500 MHz, $CDCl_3$): δ 7.55–7.51 (m, 3H), 7.37 (dd, *J* = 8.0, 1.5 Hz, 1H), 7.28 (t, *J* = 8.0 Hz, 1H), 7.21–7.17 (m, 2H), 7.12–7.09 (m, 1H), 4.82 (s, 1H), 3.32–3.18 (m, 4H), 2.32 (s, 1H), 1.49 (s, 3H); ^{13}C NMR (126 MHz, $CDCl_3$): δ 160.1 (d, *J* = 245.9 Hz), 146.4 (d, *J* = 8.4 Hz), 136.2, 130.4 (d, *J* = 3.6 Hz), 129.4 (d, *J* = 2.4 Hz), 128.7, 128.3, 127.8, 122.3 (d, *J* = 3.6 Hz), 114.5 (d, *J* = 25.2 Hz), 107.5, 74.7, 65.6, 65.4, 24.6; ^{19}F NMR (470 MHz, $CDCl_3$): δ –117.7 (*J* = 22.0 Hz); $[\alpha]_D^{24}$ –4.8 (c = 1.97, $CHCl_3$); HRMS (DART): Anal. For $C_{17}H_{17}FO_3^{+1}$ $[M+H]^+$ Calcd.: 289.1242, Found: 289.1249. The enantiopurity was determined by HPLC (hexane : 2-propanol = 50 : 1; 1.0 mL/min; using a CHIRALPAK IA-3 column (0.46 cmφ × 25 cm)): 28.1 min (minor) and 31.6 min (major).

^{1}H NMR measurement of hemiacetal derived from 4.

After fluorination of **3a**, $NaHCO_3$ aq. was added to the mixture, and extracted with Et_2O. The organic layer was dried over Na_2CO_3 and concentrated to give the crude mixture of **4a**. ^{1}H NMR measurement of **4a** in CD_3OD clearly showed the generation of hemiacetal as a diastereomeric mixture (*dr* = 6 : 4).

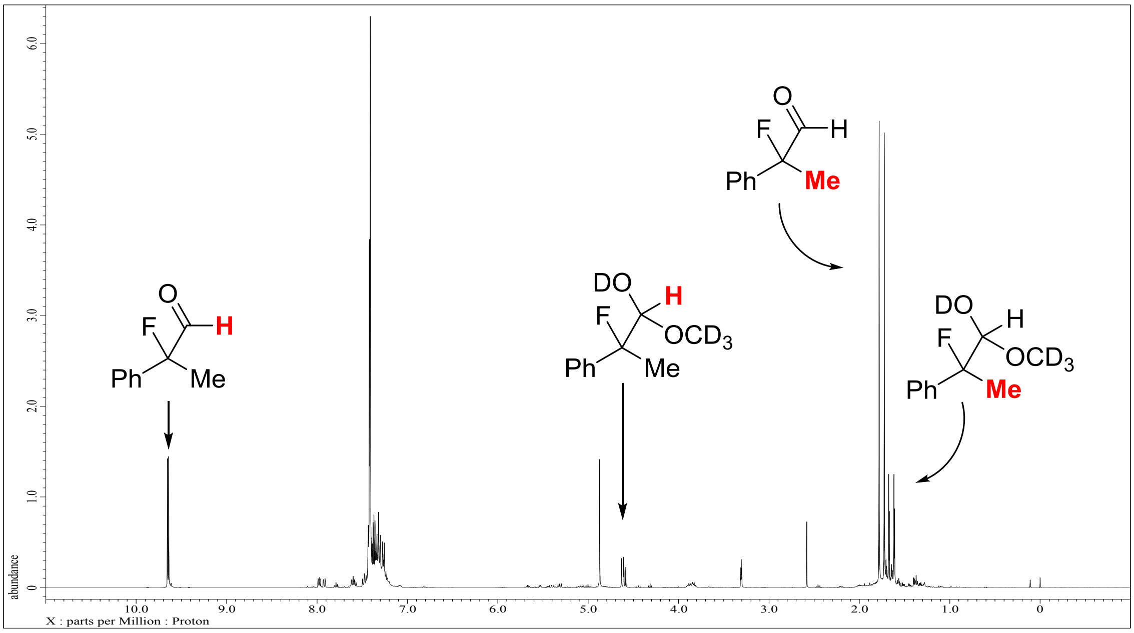

Synthesis of α-hydroxyester (Scheme 4)

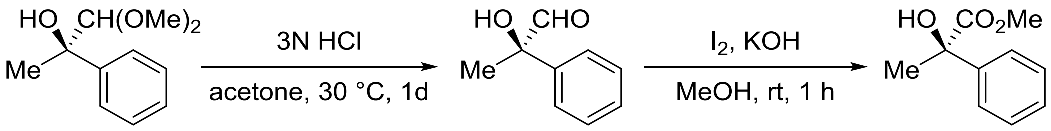

10a **13**

methyl (*R*)-2-hydroxy-2-phenylpropanoate [(*R*)-13, 91% *ee*]

The reaction was carried out according to the reported procedure.[5] To a solution of α-hydroxyacetal (*R*)-**10a** (0.335 mmol, 91% *ee*) in acetone (4.8 mL) was added 3N HCl (2.1 mL) at 0 °C. The mixture was stirred for 1 d at 30 °C. After being quenched with K_2CO_3 aq., acetone was removed under reduced pressure. The mixture was extracted with ethyl acetate, and the organic layer was dried over Na_2SO_4, and concentrated. The residue was dissolved in MeOH (11.2 mL) and cooled to 0 °C. To this solution were added KOH (0.872 mmol, 2.6 equiv.) and I_2 (0.436 mmol, 1.3 equiv.) successively. The mixture was stirred for 1h and quenched by adding 1.2N HCl. Sat. $Na_2S_2O_3$ aq. was added until the mixture turned colorless. MeOH was removed under reduced pressure and the mixture was extracted with ethyl acetate. The organic layer was

dried over Na_2SO_4 and concentrated. The residue was purified silica gel column chromatography (hexane : MTBE = 9 : 1–2 : 1) to give 60% yield of (*R*)-**13** (colorless oil). ^{1}H NMR (400 MHz, $CDCl_3$): δ 7.56–7.54 (m, 2H), 7.38–7.34 (m, 2H), 7.31–7.28 (m, 1H), 3.78 (s, 3H), 3.75 (s, 1H), 1.79 (s, 3H); ^{13}C NMR (100 MHz, $CDCl_3$): δ 176.1, 142.6, 128.3, 127.8, 125.1, 75.7, 53.2, 26.6; $[\alpha]_D^{21}$ –51.9 (c = 0.98, $CHCl_3$); HRMS (DART): Anal. For $C_{10}H_{16}N_1O_3^{+1}$ [$M+NH_4^+$] Calcd.: 198.1130, Found: 198.1130; The enantiopurity was determined by HPLC (hexane : 2-propanol = 50 : 1, 1.0 mL/min) using a CHIRALPAK AD-3 column (0.46 cmφ x 25 cm): major isomer 10.2 min and minor isomer 8.9 min. The absolute configuration of the major enantiomer was determined to be *R* by comparing the specific rotation with that in the literature.[6]

References

1. (a) V. A. Soloshonok, C. Roussel, O. Kitagawa, A. E. Sorochinsky, *Chem. Soc. Rev.* 2012, **41**, 4180. (b) J. Han, D. J. Nelson, A. E. Sorochinsky, V. A. Soloshonok, *Curr. Org. Synth.* 2011, **8**, 310. (c) H. Ueki, M. Yasumoto, V. A. Soloshonok, *Tetrahedron: Asymmetry* 2010, **21**, 1396. (d) V. A. Soloshonok, H. Ueki, M. Yasumoto, S. Mekala, J. S. Hirschi, D. A. Singleton, *J. Am. Chem. Soc.* 2007, **129**, 12112. (e) V. A. Soloshonok, *Angew. Chem. Int. Ed.* 2006, **45**, 766.
2. T. Ooi, M. Kameda, K. Maruoka, *J. Am. Chem. Soc.* 2003, **125**, 5139.
3. K. Shibatomi, T. Muto, Y. Sumikawa, A. Narayama, S. Iwasa, *Synlett* 2009, 241.
4. S. Brandes, B. Niess, M. Bella, A. Prieto, J. Overgaard, K. A. Jørgensen, *Chem. Eur. J.* 2006, **12**, 6039.
5. S.-K. Tian, R. Hong, L. Deng, *J. Am. Chem. Soc.* 2003, **125**, 9900.
6. L. C. Wieland, H. Deng, M. L. Snapper, A. H. Hoveyda, *J. Am. Chem. Soc.* 2005, **127**, 15453.

NMR spectra and HPLC traces

^{1}H NMR spectrum [(*R*)-**15**]

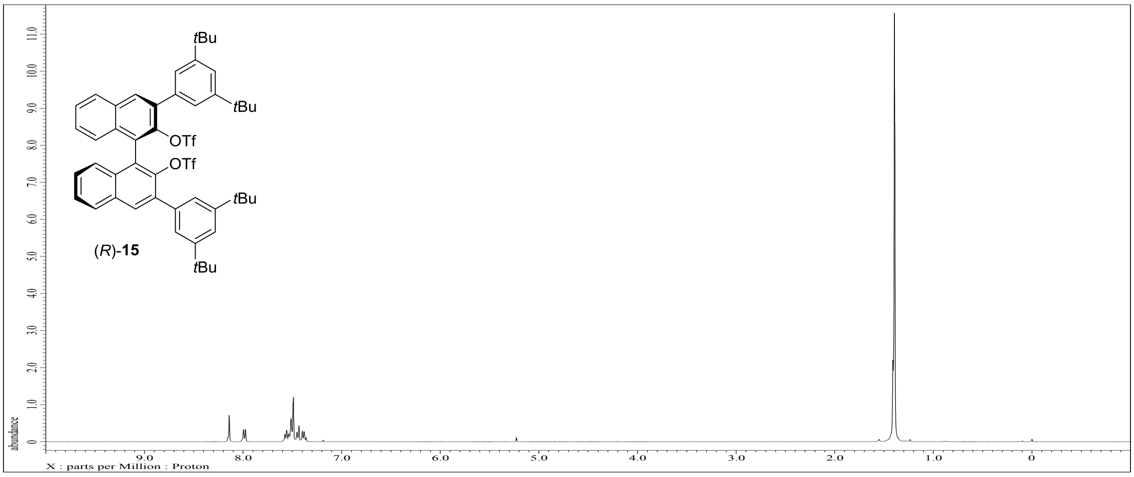

^{13}C NMR spectrum [(*R*)-**15**]

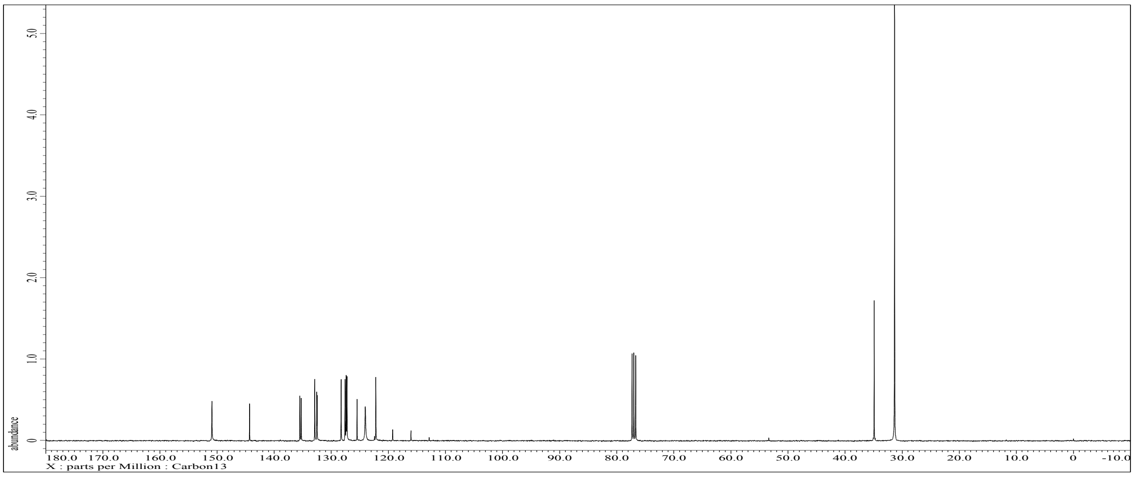

^{19}F NMR spectrum [(*R*)-**15**]

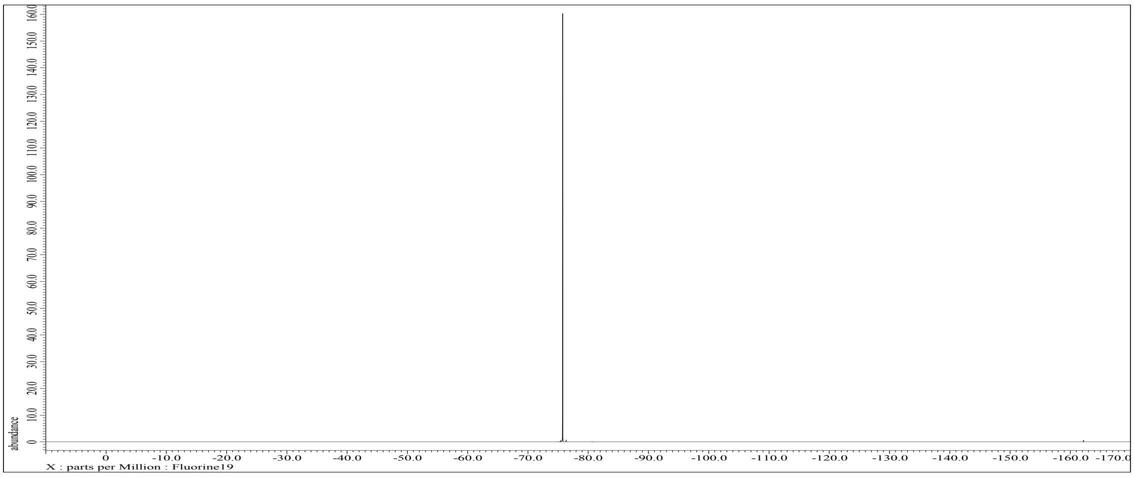

^{1}H NMR spectrum [(*R*)-**16**]

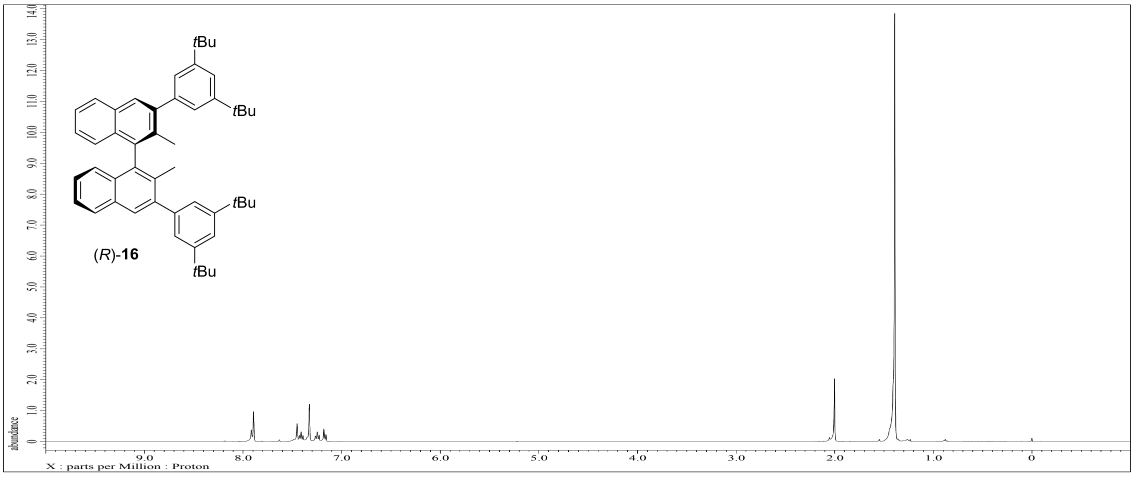

^{13}C NMR spectrum [(*R*)-**16**]

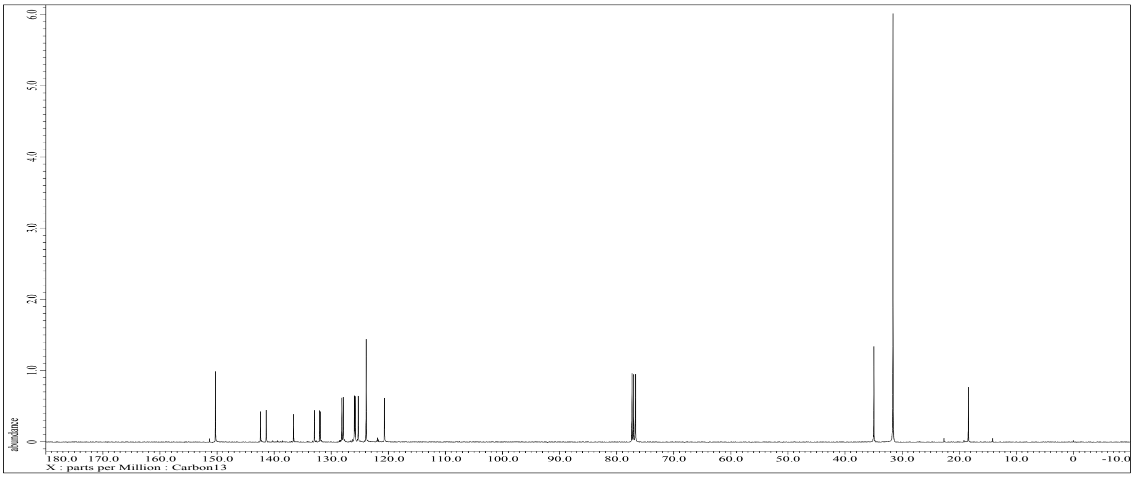

^{1}H NMR spectrum [(*R*)-**2b**]

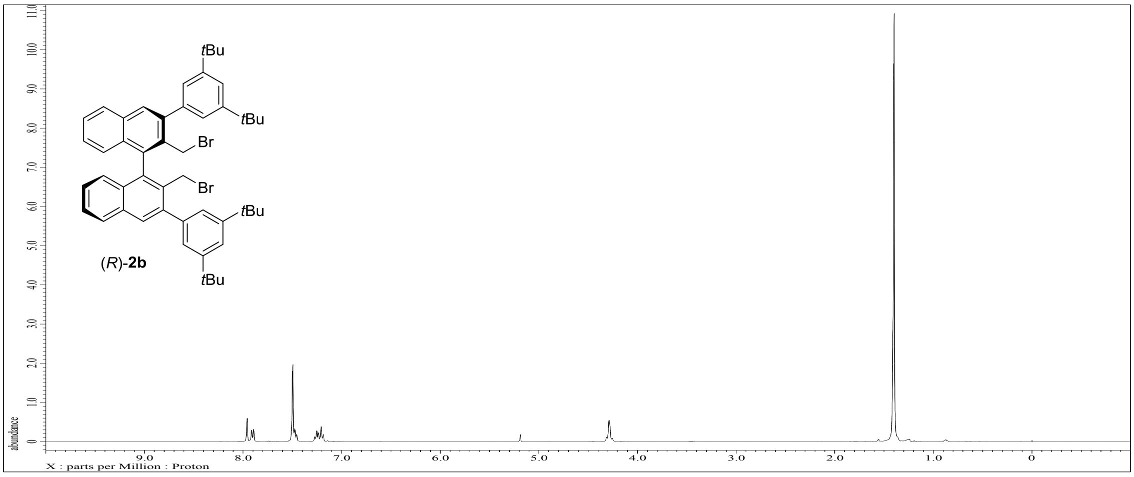

^{13}C NMR spectrum [(*R*)-**2b**]

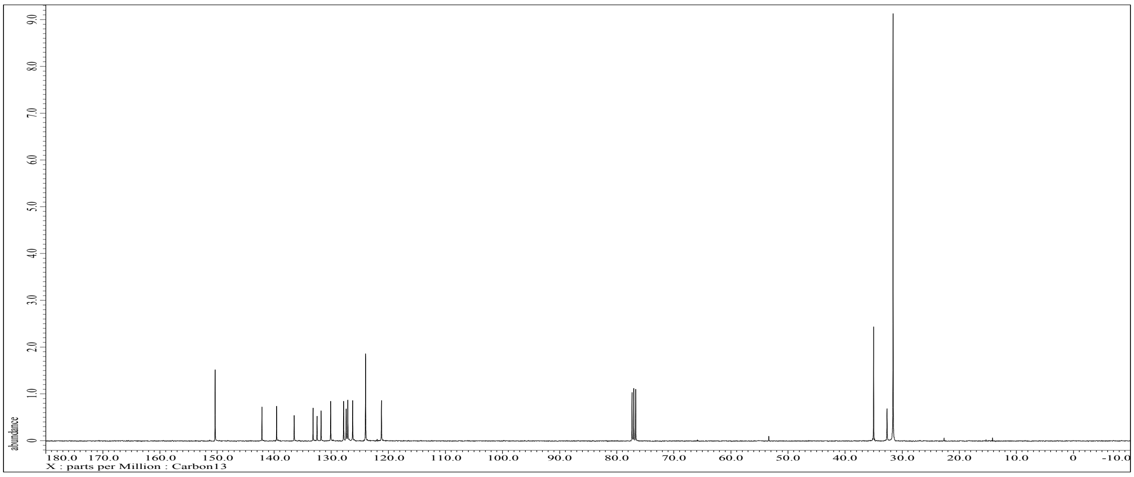

^{1}H NMR spectrum [(*R*)-**17a**]

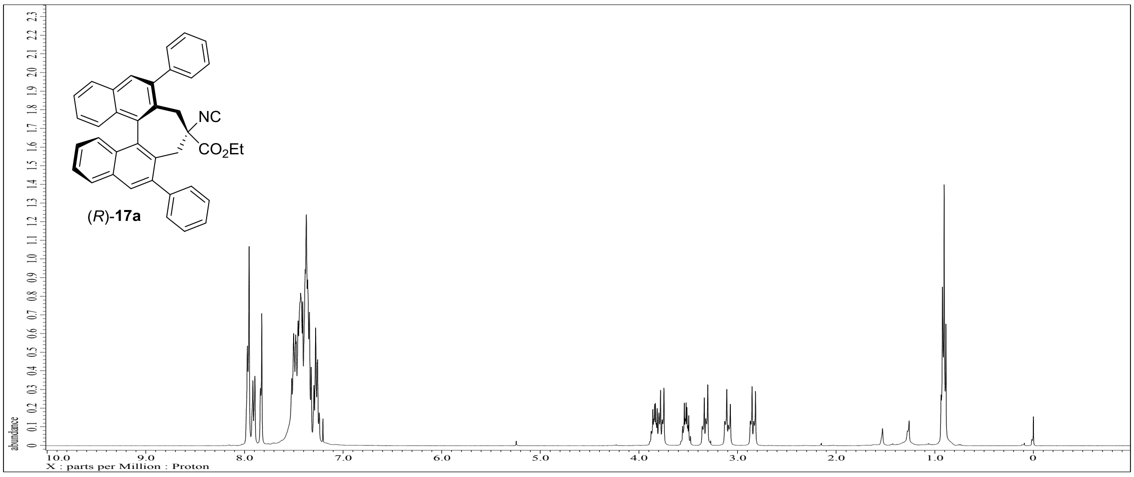

^{13}C NMR spectrum [(*R*)-**17a**]

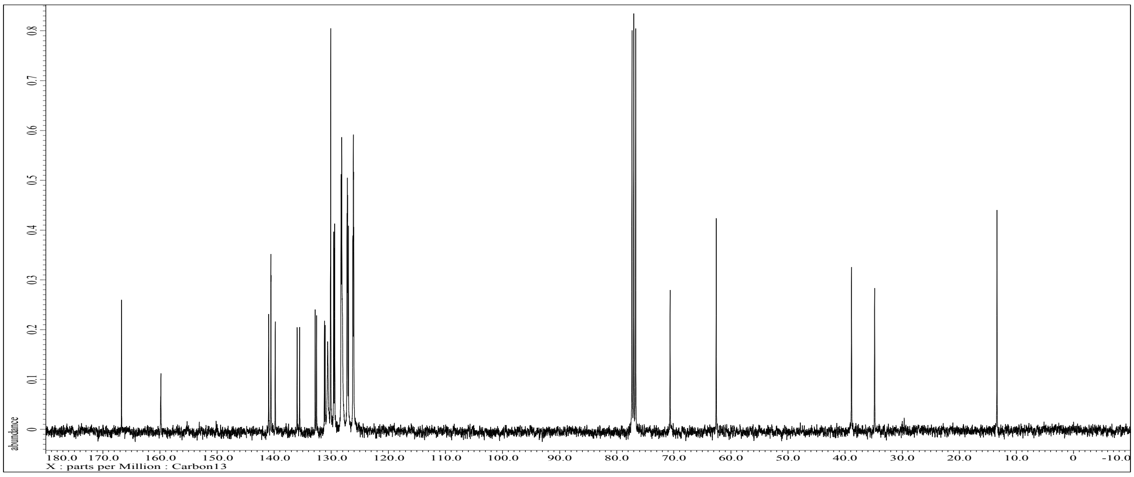

^{1}H NMR spectrum [(*R*)-**17b**]

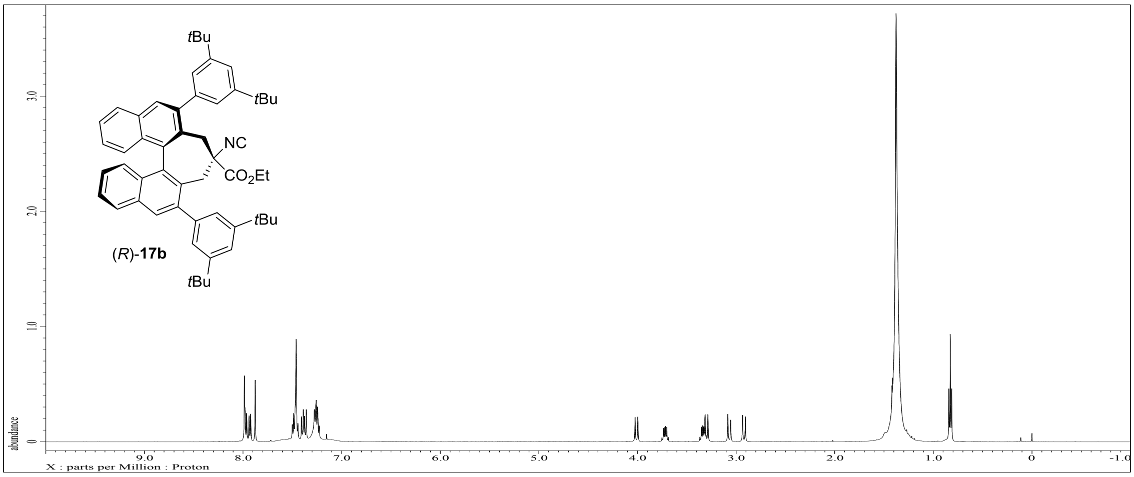

^{13}C NMR spectrum [(*R*)-**17b**]

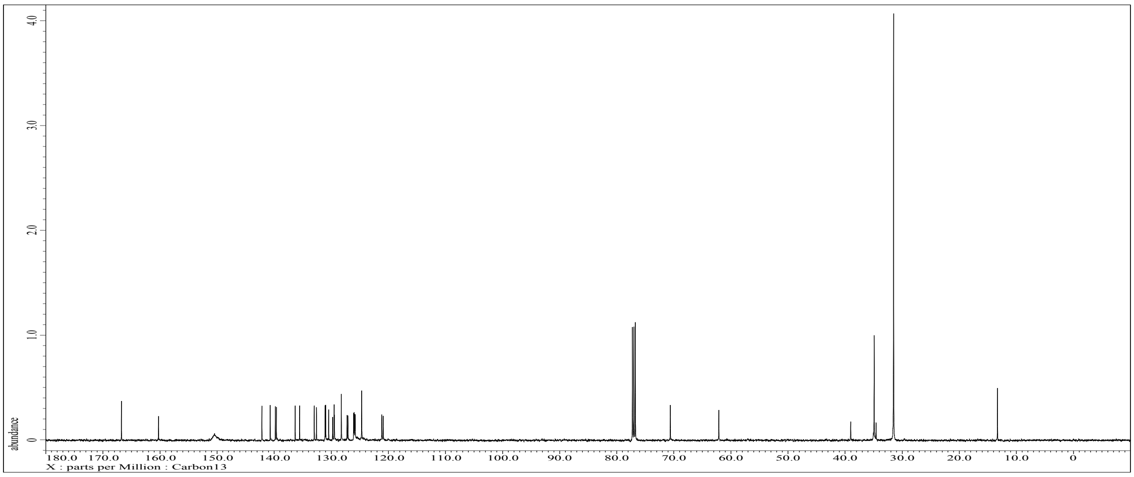

^{1}H NMR spectrum [(*R*)-**1a**]

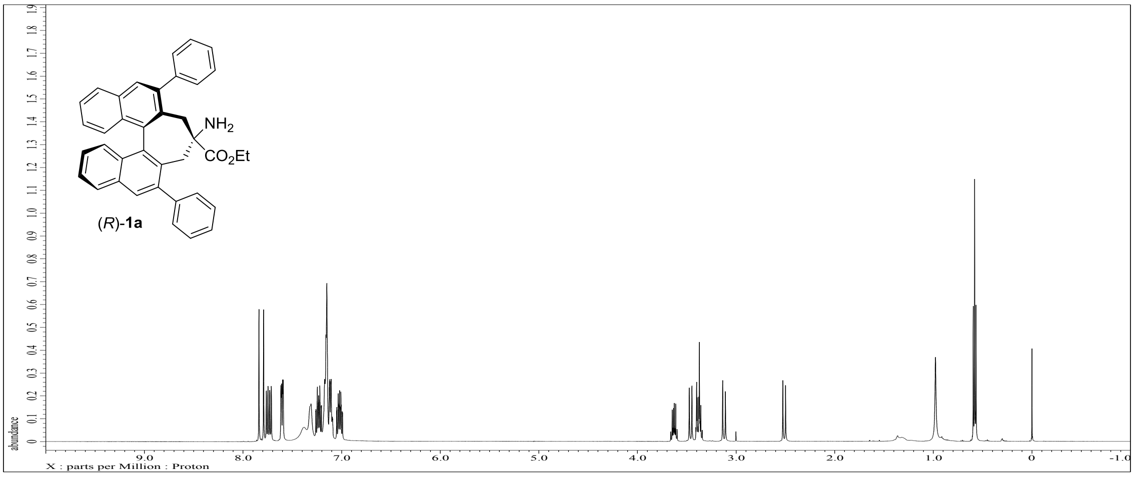

^{13}C NMR spectrum [(*R*)-**1a**]

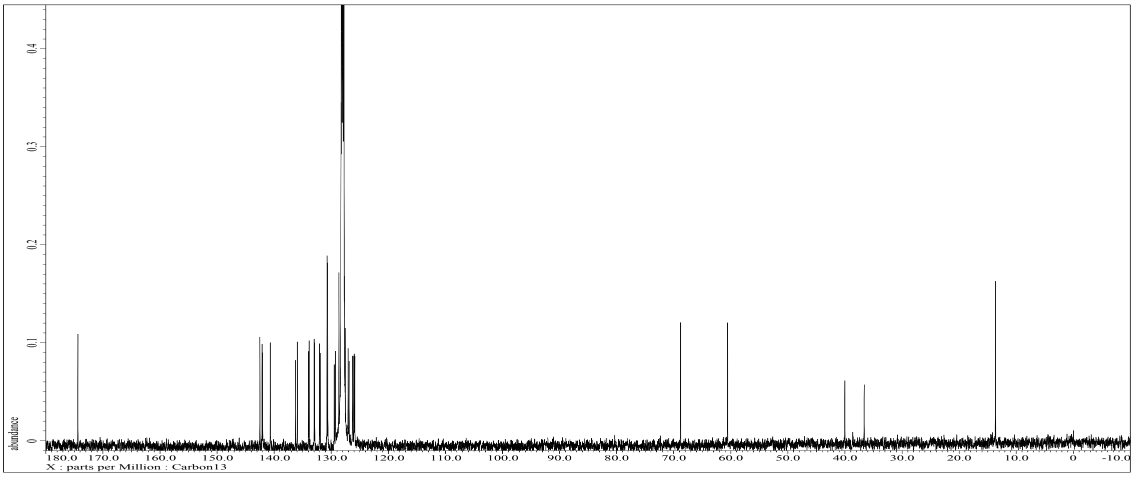

^{1}H NMR spectrum [(*R*)-**1b**]

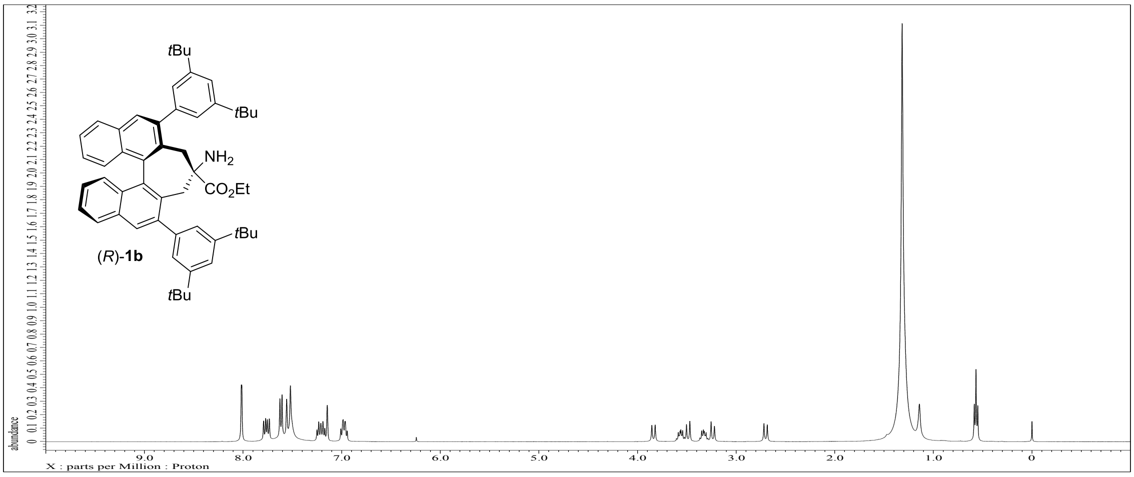

^{13}C NMR spectrum [(*R*)-**1b**]

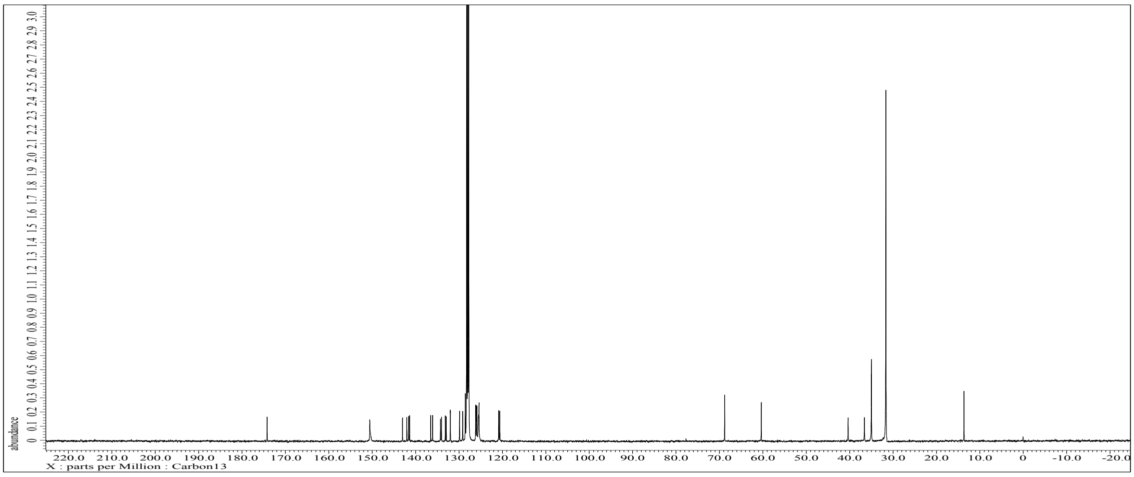

HPLC *optically active* [(*R*)-**1b**]

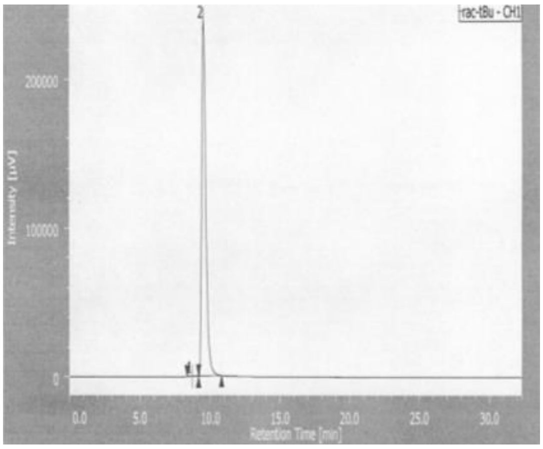

#	ピーク名	CH	tR [min]	面積 [µV·sec]	高さ [µV]	面積%	高さ%	定量値	NTP	分離度	シンメトリー係数	警告
1	Unknown	1	8.692	956	96	0.019	0.040	N/A	14571	1.962	0.722	
2	Unknown	1	9.483	4909183	239404	99.981	99.960	N/A	5297	N/A	1.556	

HPLC *racemic* (**1b)**

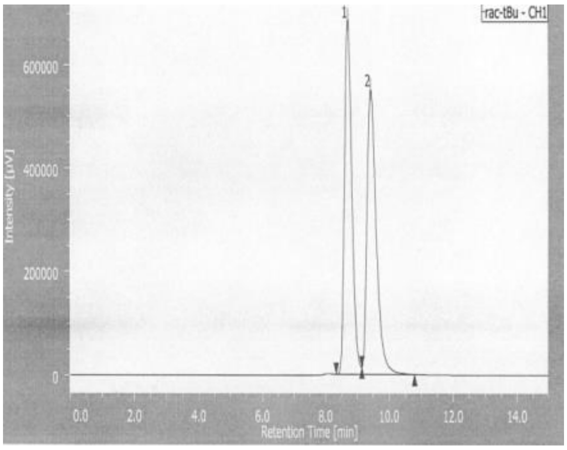

#	ピーク名	CH	tR [min]	面積 [µV·sec]	高さ [µV]	面積%	高さ%	定量値	NTP	分離度	シンメトリー係数	警告
1	Unknown	1	8.692	11063983	686069	49.752	55.556	N/A	6779	1.546	1.334	
2	Unknown	1	9.417	11174501	548839	50.248	44.444	N/A	5291	N/A	1.542	

^{1}H NMR spectrum [(*S*)-**5a**]

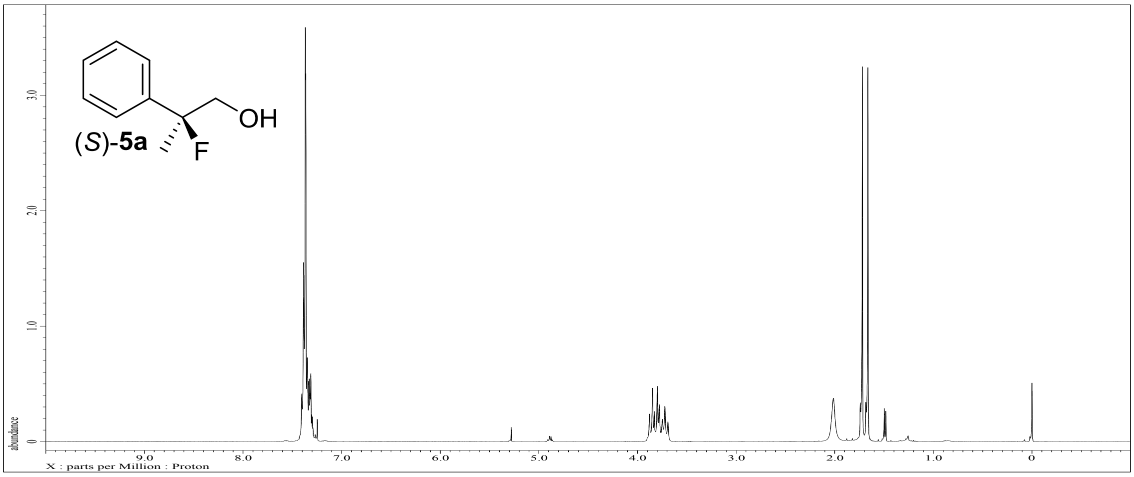

^{13}C NMR spectrum [(*S*)-**5a**]

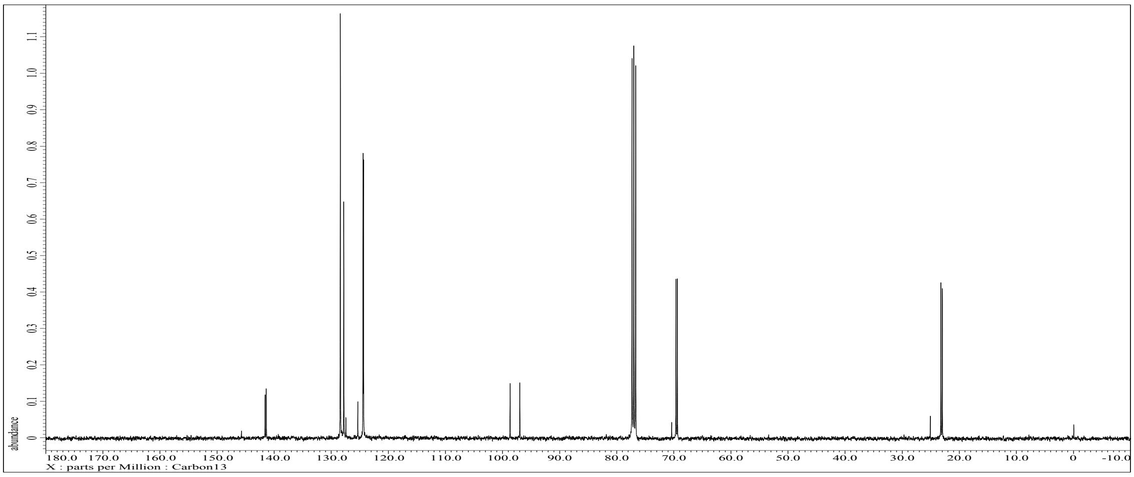

^{19}F NMR spectrum [(*S*)-**5a**]

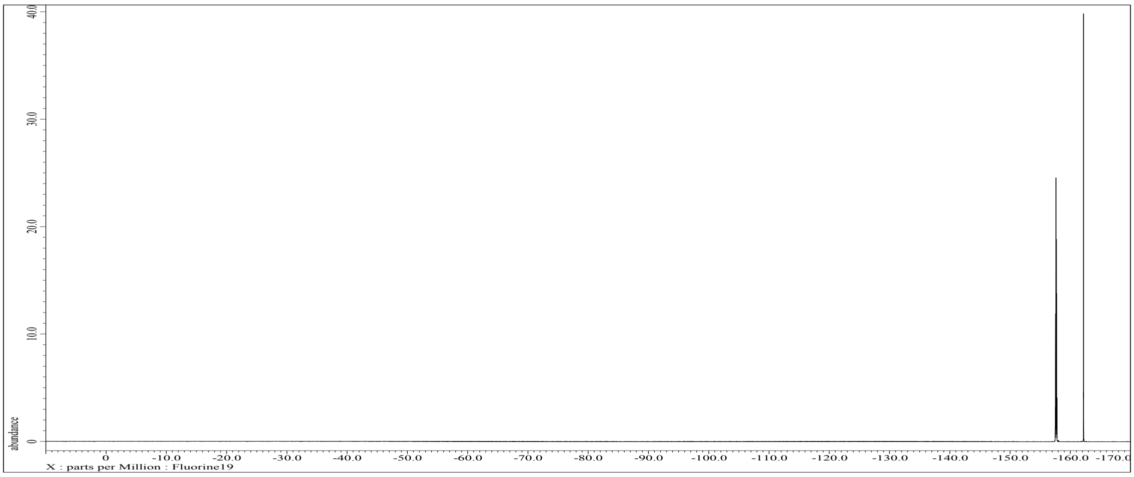

^{1}H NMR spectrum [(*S*)-**18a**]

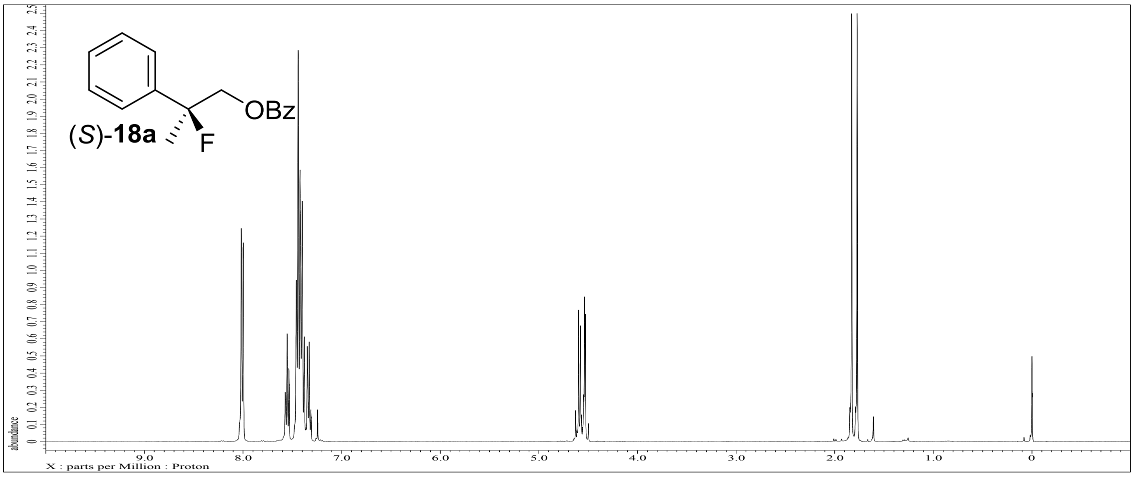

^{13}C NMR spectrum [(*S*)-**18a**]

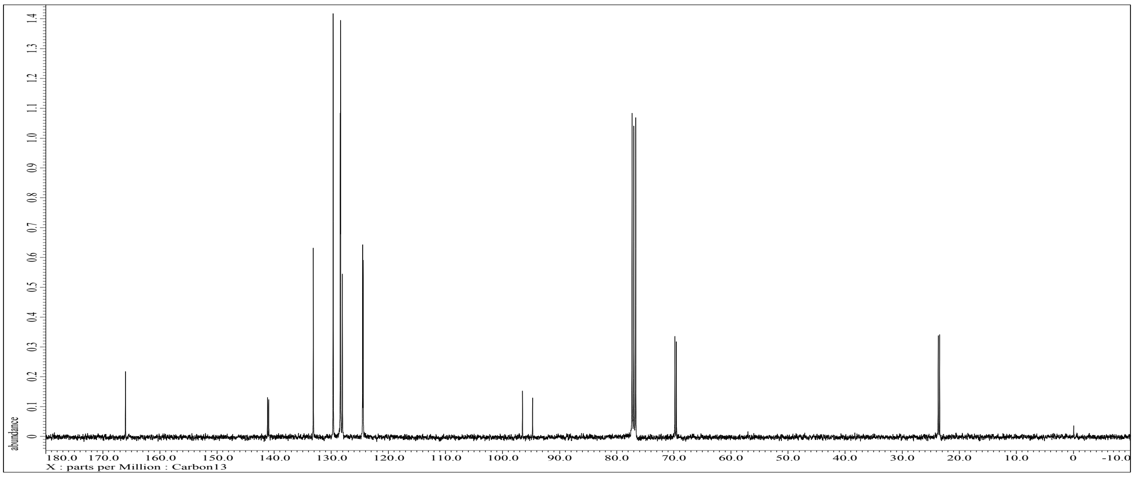

^{19}F NMR spectrum [(*S*)-**18a**]

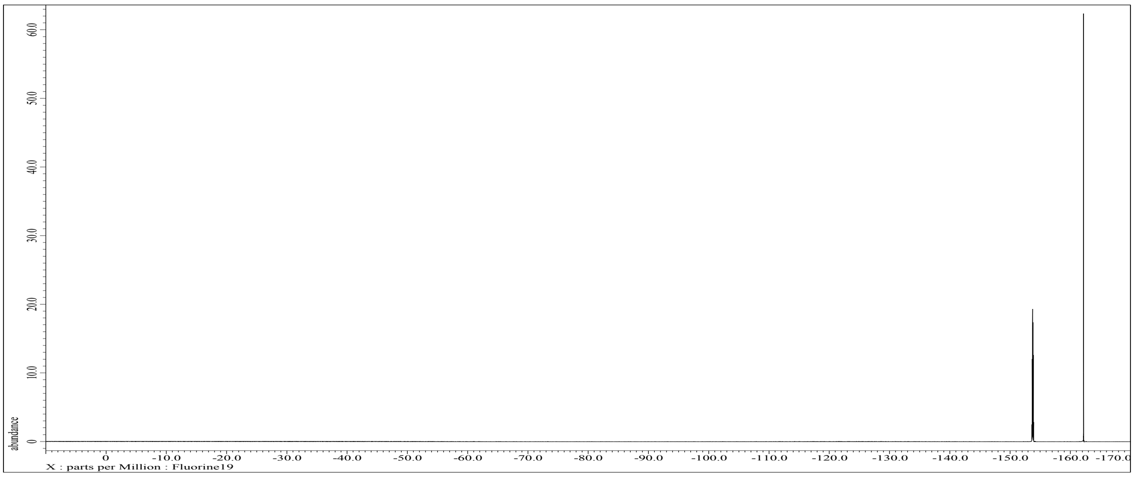

HPLC *optically active* [(*S*)-**18a**]

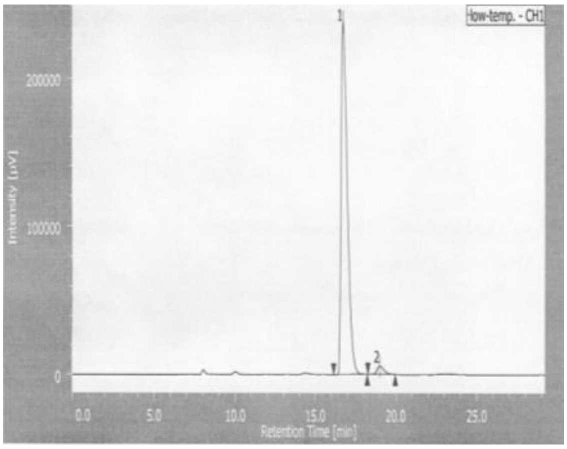

#	ピーク名	CH	tR [min]	面積 [μV·sec]	高さ [μV]	面積%	高さ%	定量値	NTP	分離度	シンメトリー係数	警告
1	Unknown	1	16.725	6385238	237257	97.416	97.657	N/A	9007	3.113	1.969	
2	Unknown	1	19.017	169374	5692	2.584	2.343	N/A	9729	N/A	1.454	

HPLC *racemic* (**18a**)

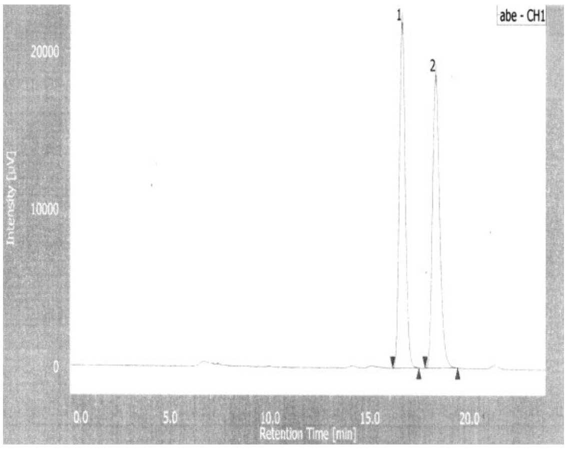

#	ピーク名	CH	tR [min]	面積 [μV·sec]	高さ [μV]	面積%	高さ%	定量値	NTP	分離度	シンメトリー係数	警告
1	Unknown	1	16.592	462277	21958	49.826	54.137	N/A	14441	2.807	1.191	
2	Unknown	1	18.275	465498	18602	50.174	45.863	N/A	12639	N/A	1.311	

^{1}H NMR spectrum (**5b**)

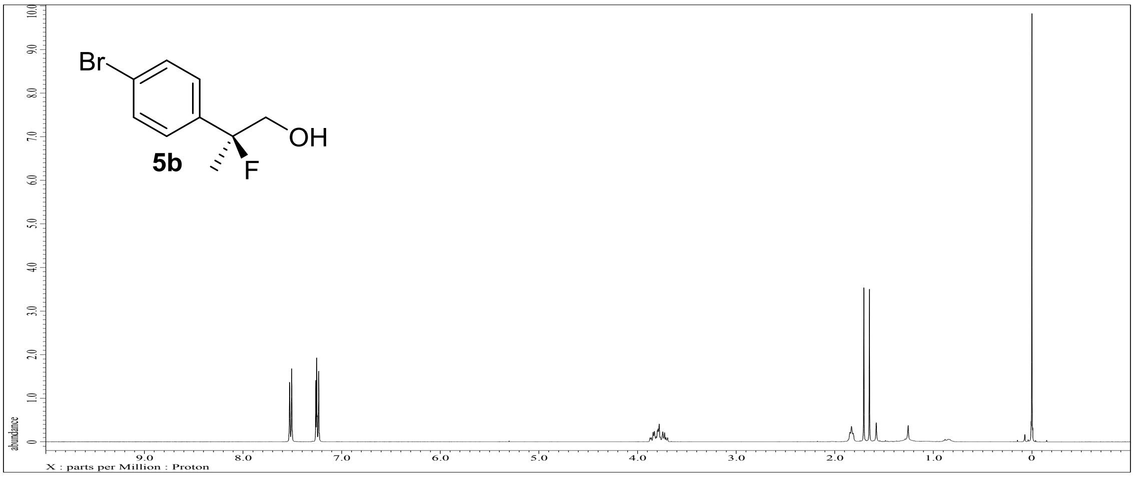

^{13}C NMR spectrum (**5b**)

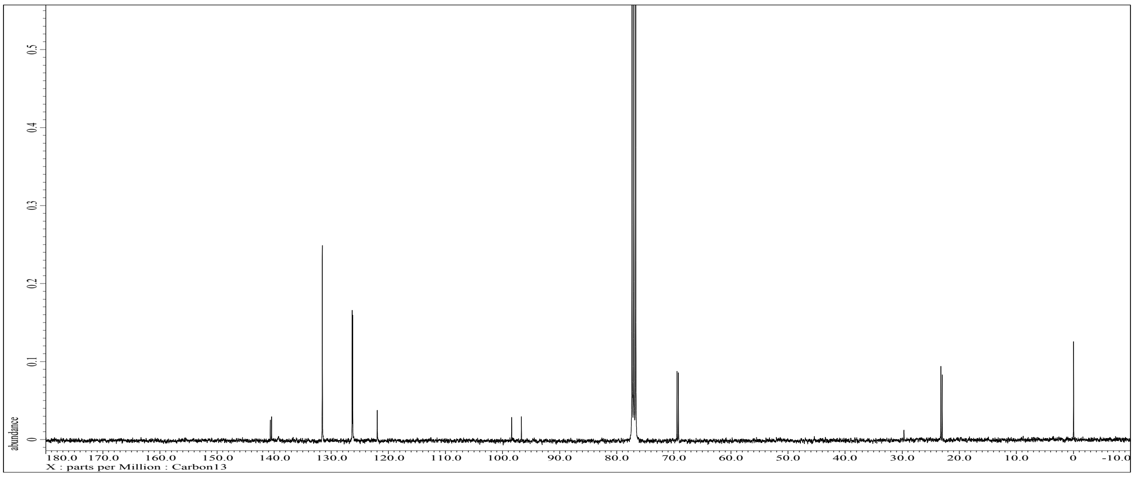

^{19}F NMR spectrum (**5b**)

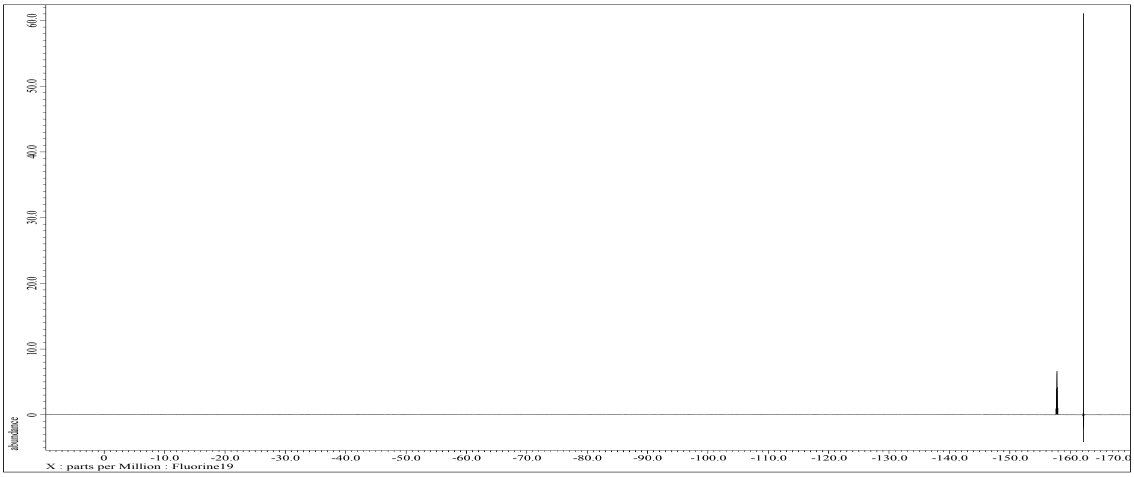

^{1}H NMR spectrum (**18b**)

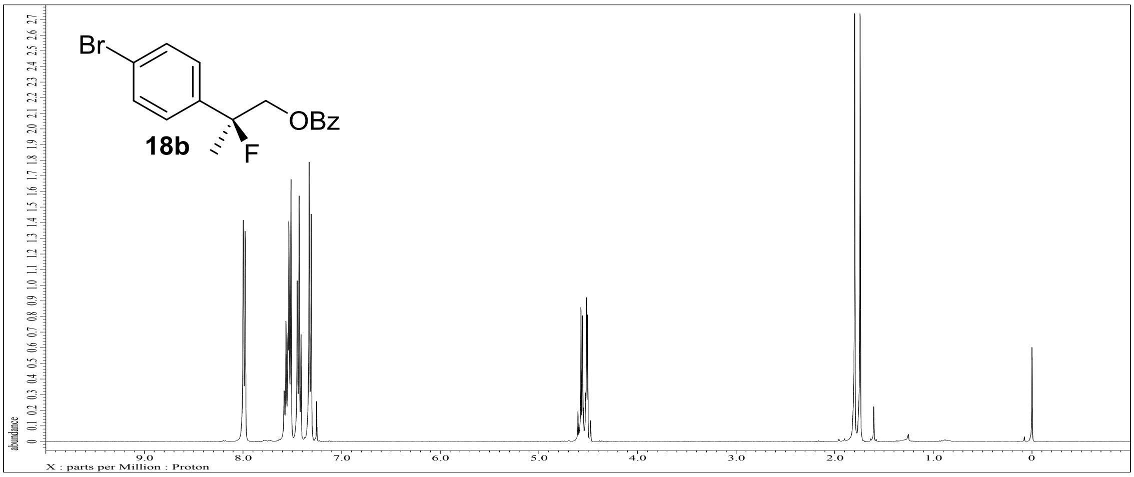

^{13}C NMR spectrum (**18b**)

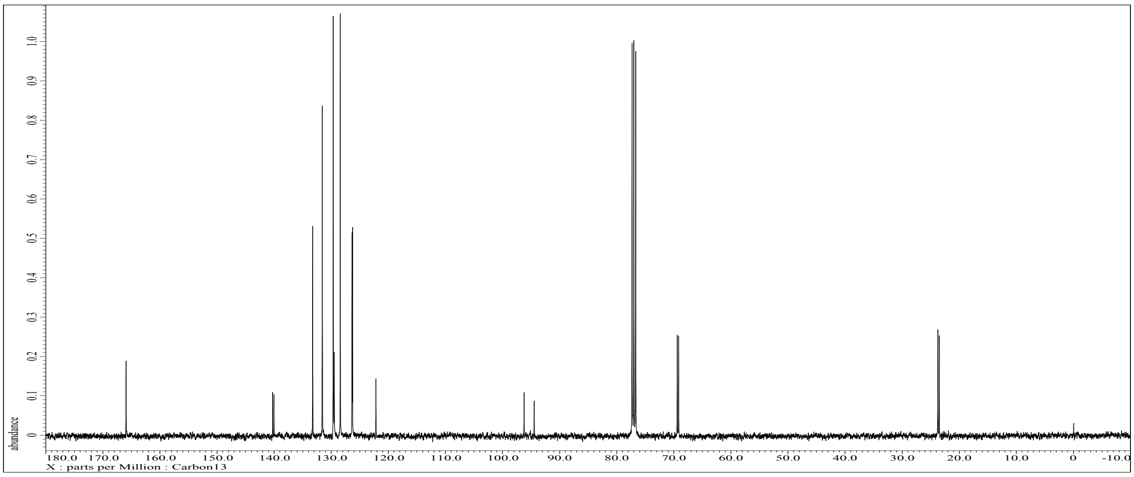

^{19}F NMR spectrum (**18b**)

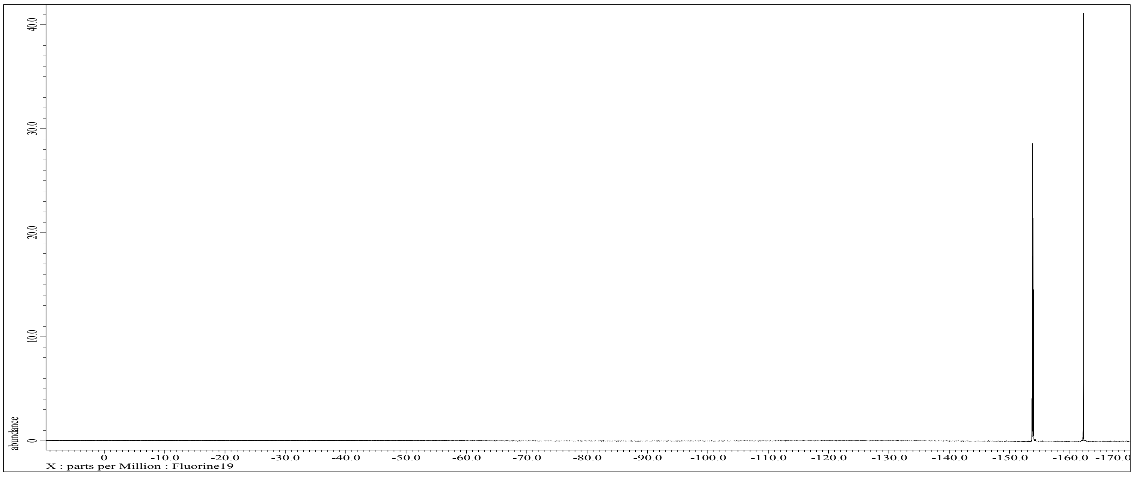

HPLC *optically active* (**18b**)

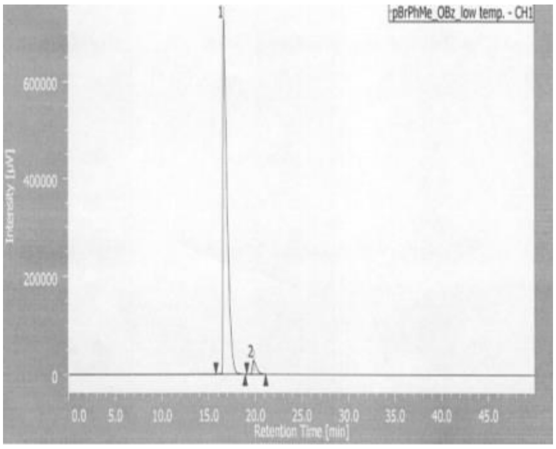

#	ピーク名	CH	tR [min]	面積 [µV·sec]	高さ [µV]	面積%	高さ%	定量値	NTP	分離度	シンメトリー係数	警告
1	Unknown	1	16.642	22204070	726475	96.166	96.319	N/A	7557	4.006	3.359	
2	Unknown	1	19.817	885275	27760	3.834	3.681	N/A	9267	N/A	1.888	

HPLC *racemic* (**18b**)

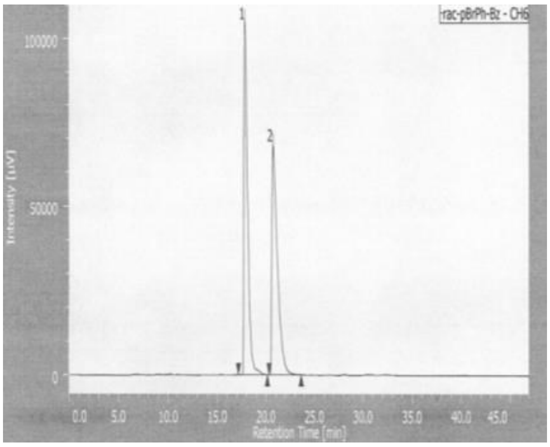

#	ピーク名	CH	tR [min]	面積 [µV·sec]	高さ [µV]	面積%	高さ%	定量値	NTP	分離度	シンメトリー係数	警告
1	Unknown	6	17.840	2780841	104997	50.783	60.688	N/A	11834	3.520	2.469	
2	Unknown	6	20.725	2695071	68015	49.217	39.312	N/A	7044	N/A	3.316	

^{1}H NMR spectrum (**5c**)

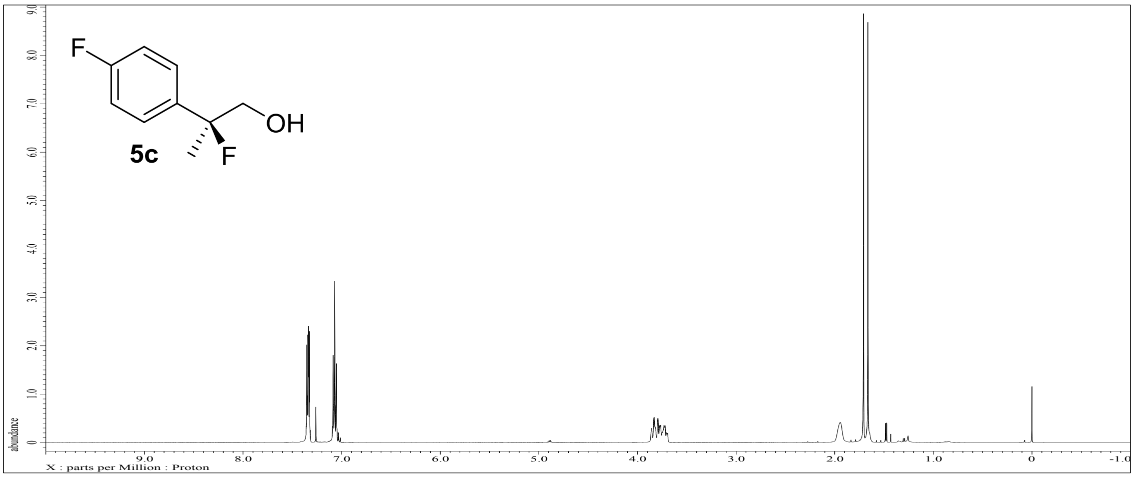

^{13}C NMR spectrum (**5c**)

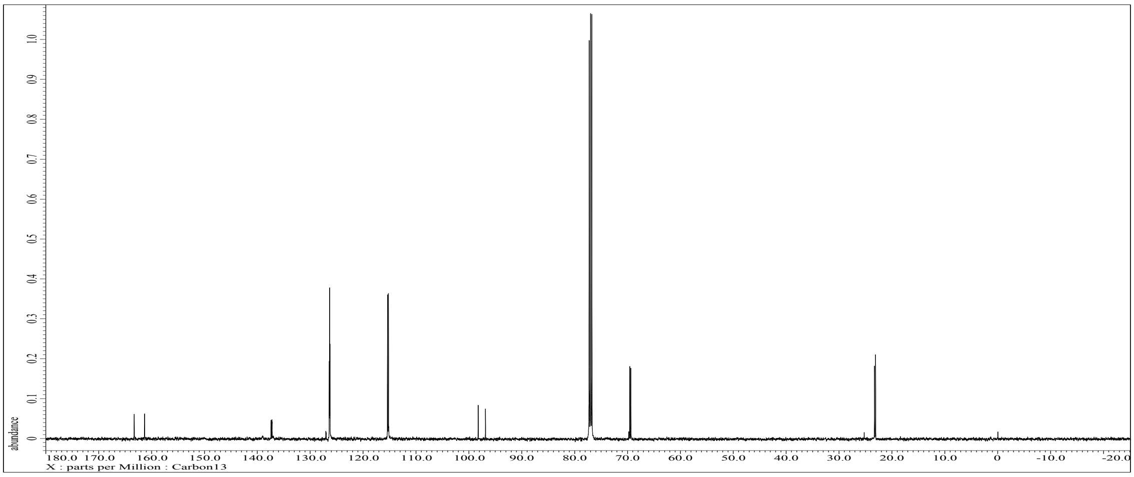

^{19}F NMR spectrum (**5c**)

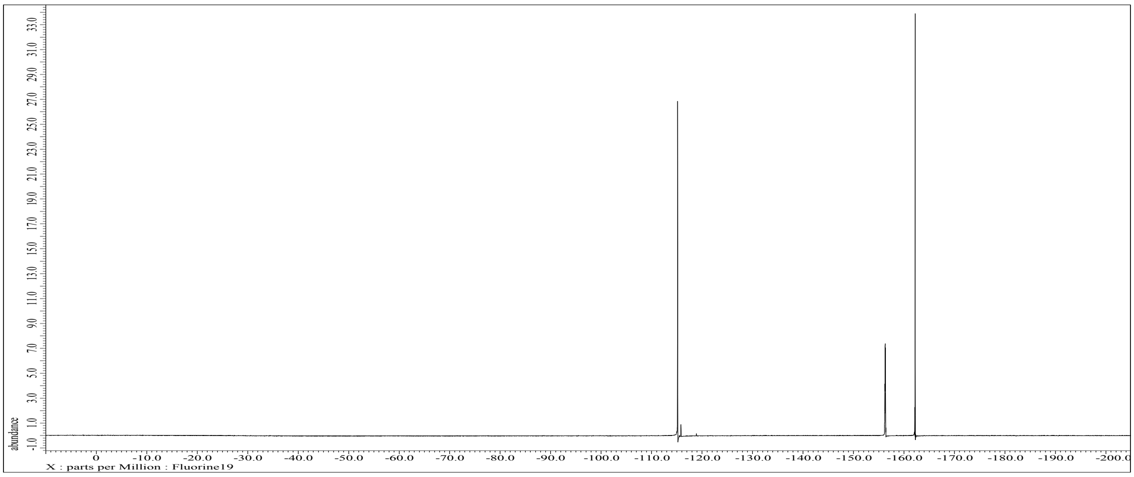

HPLC *optically active* (**5c**)

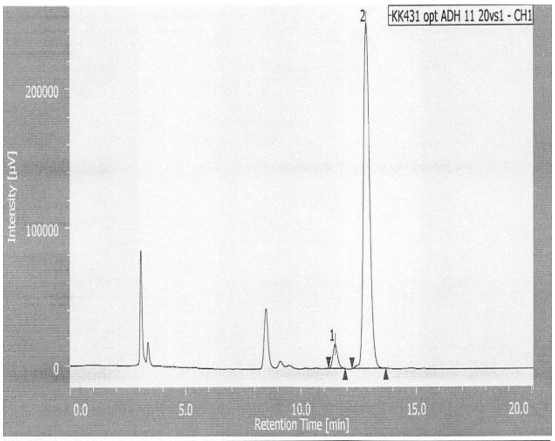

#	ピーク名	CH	tR [min]	面積 [µV·sec]	高さ [µV]	面積%	高さ%	定量値	NTP	分離度	シンメトリー係数	警告
1	Unknown	1	11.483	247386	17520	4.963	6.566	N/A	16088	3.128	1.204	
2	Unknown	1	12.825	4737414	249323	95.037	93.434	N/A	10610	N/A	1.323	

HPLC *racemic* (**5c**)

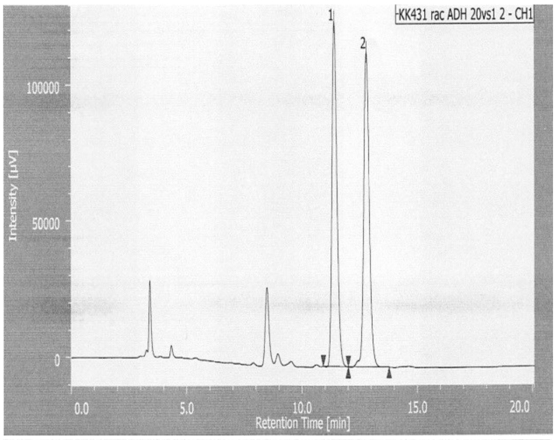

#	ピーク名	CH	tR [min]	面積 [µV·sec]	高さ [µV]	面積%	高さ%	定量値	NTP	分離度	シンメトリー係数	警告
1	Unknown	1	11.400	1899933	126352	49.276	52.263	N/A	13536	3.380	1.253	
2	Unknown	1	12.792	1955731	115410	50.724	47.737	N/A	13914	N/A	1.199	

^{1}H NMR spectrum (**5d**)

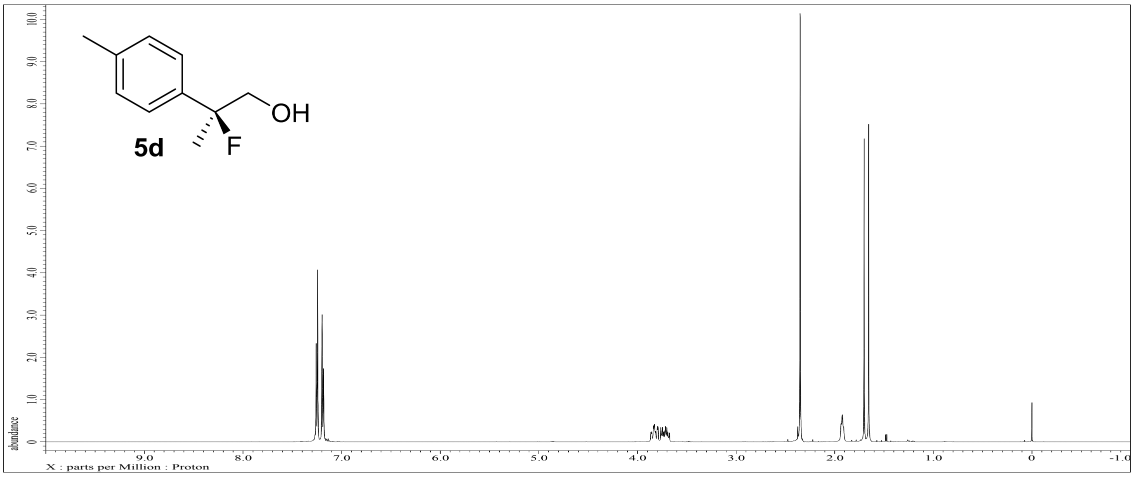

^{13}C NMR spectrum (**5d**)

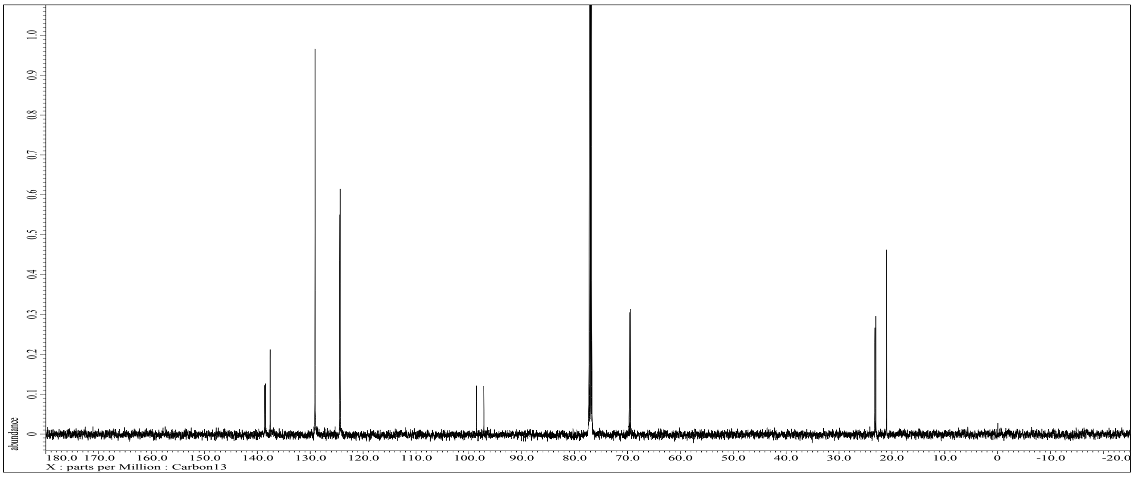

^{19}F NMR spectrum (**5d**)

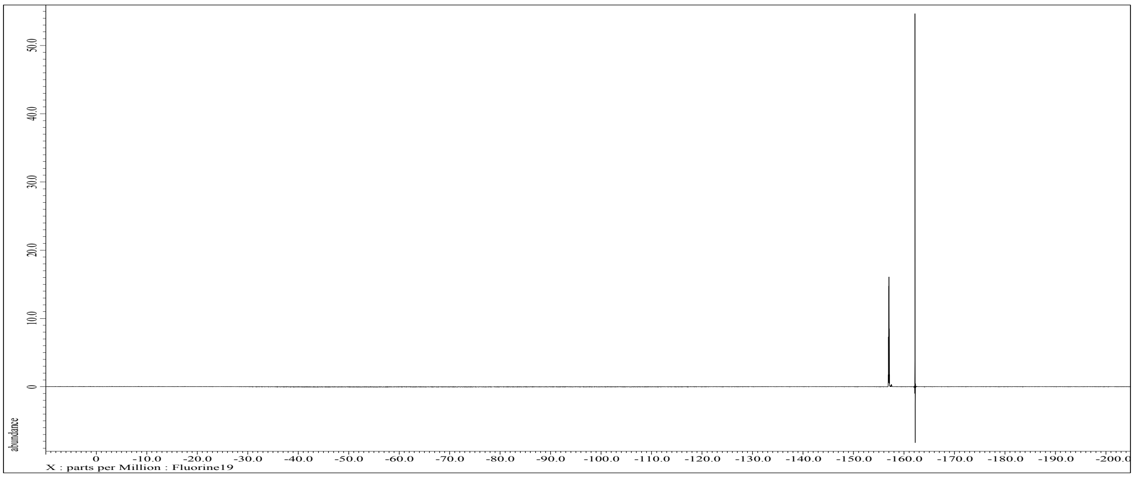

HPLC *optically active* (**5d**)

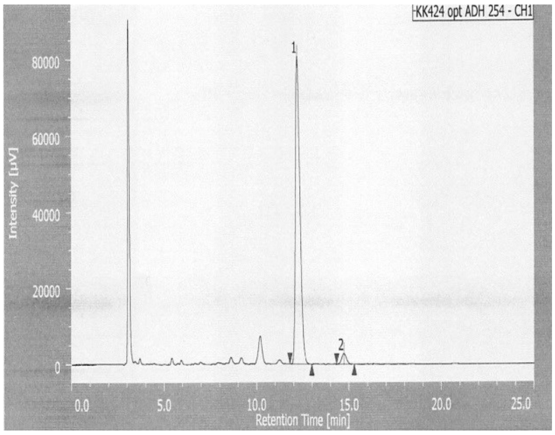

#	ピーク名	CH	tR [min]	面積 [μV·sec]	高さ [μV]	面積%	高さ%	定量値	NTP	分離度	シンメトリー係数	警告
1	Unknown	1	12.200	1374930	80619	96.523	96.646	N/A	12031	5.559	1.508	
2	Unknown	1	14.725	49526	2798	3.477	3.354	N/A	15983	N/A	1.056	

HPLC *racemic* (**5d**)

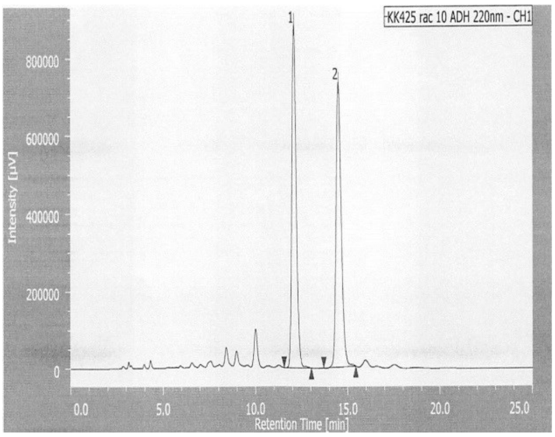

#	ピーク名	CH	tR [min]	面積 [μV·sec]	高さ [μV]	面積%	高さ%	定量値	NTP	分離度	シンメトリー係数	警告
1	Unknown	1	12.075	14588707	884459	50.288	54.687	N/A	12825	5.207	1.274	
2	Unknown	1	14.492	14421532	732855	49.712	45.313	N/A	13214	N/A	1.288	

^{1}H NMR spectrum (**5e**)

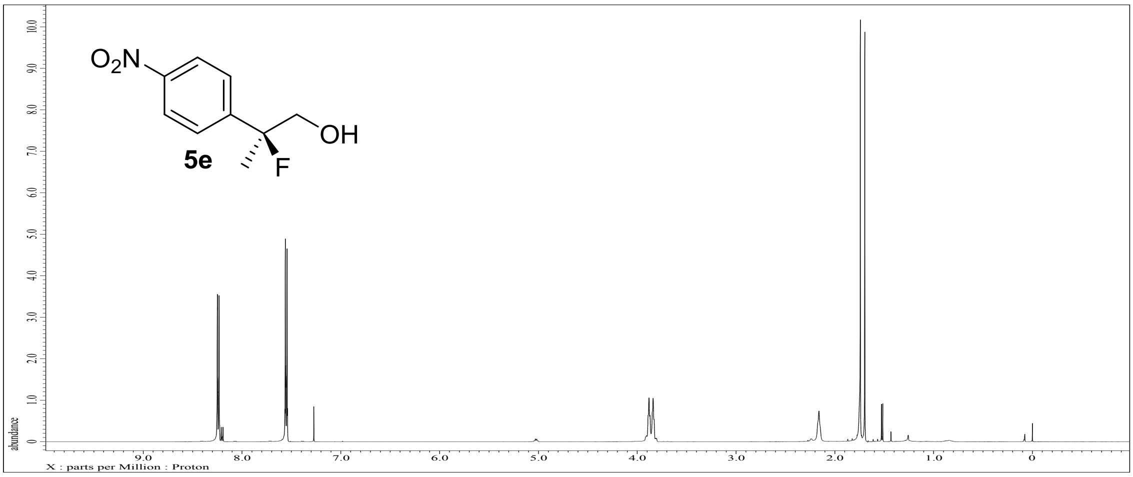

^{13}C NMR spectrum (**5e**)

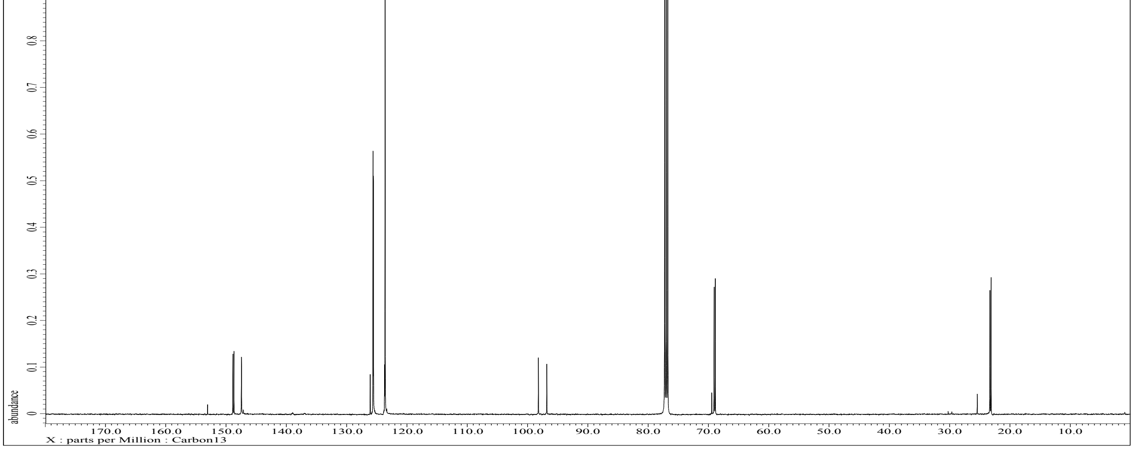

^{19}F NMR spectrum (**5e**)

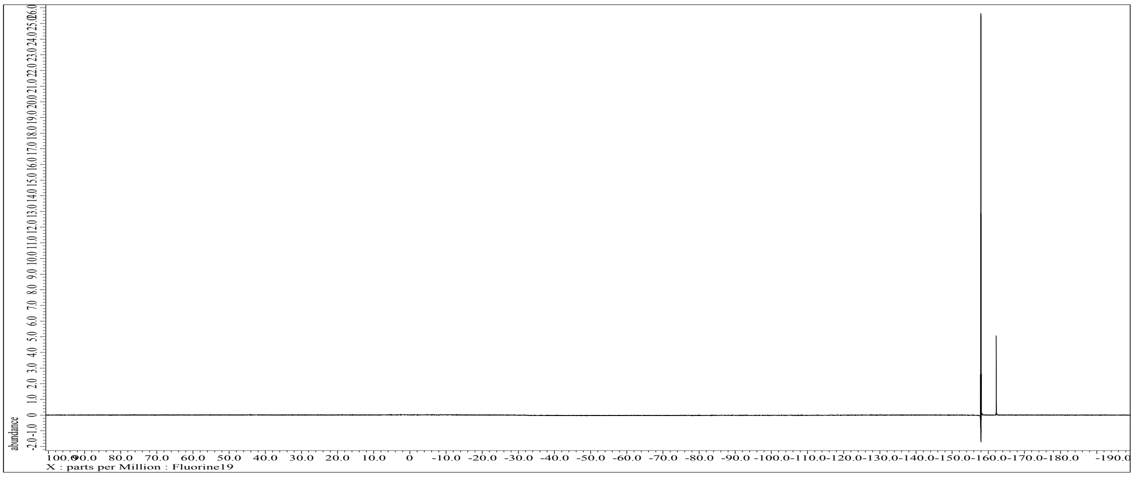

HPLC *optically active* (**5e**)

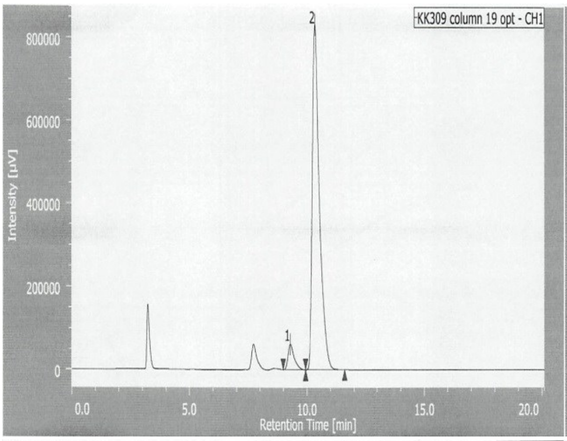

#	ピーク名	CH	tR [min]	面積 [μV·sec]	高さ [μV]	面積%	高さ%	定量値	NTP	分離度	シンメトリー係数	警告
1	Unknown	1	9.292	1097510	61941	5.888	6.924	N/A	7241	2.222	1.573	
2	Unknown	1	10.350	17543077	832612	94.112	93.076	N/A	6382	N/A	1.657	

HPLC *racemic* (**5e**)

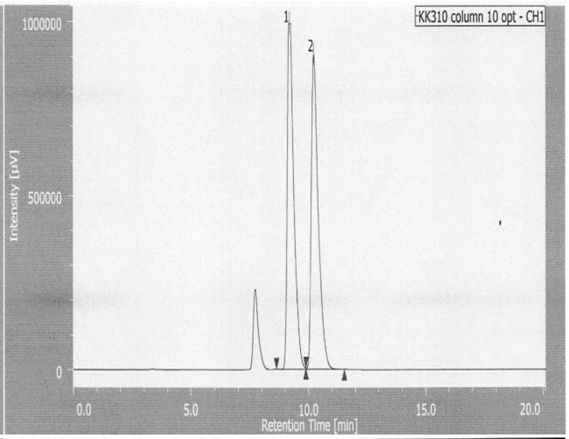

#	ピーク名	CH	tR [min]	面積 [μV·sec]	高さ [μV]	面積%	高さ%	定量値	NTP	分離度	シンメトリー係数	警告
1	Unknown	1	9.200	19448024	994777	50.734	52.332	N/A	5712	2.075	1.709	
2	Unknown	1	10.242	18885666	906120	49.266	47.668	N/A	6210	N/A	1.660	

^{1}H NMR spectrum (**5g**)

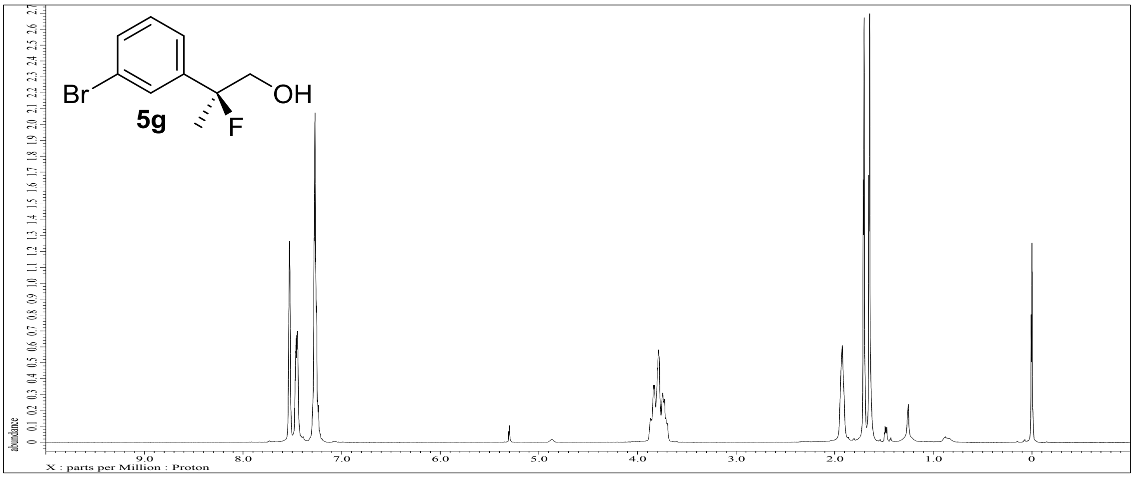

^{13}C NMR spectrum (**5g**)

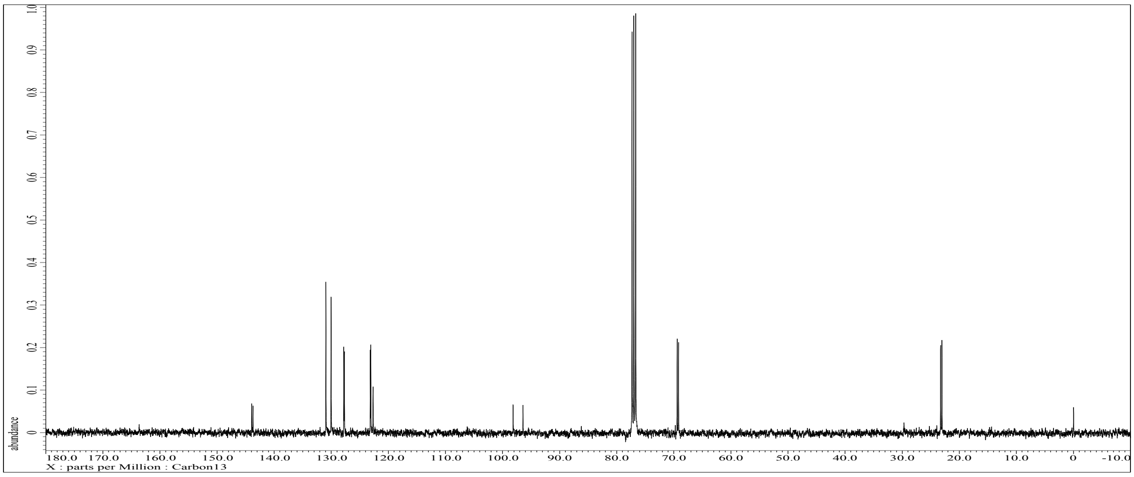

^{19}F NMR spectrum (**5g**)

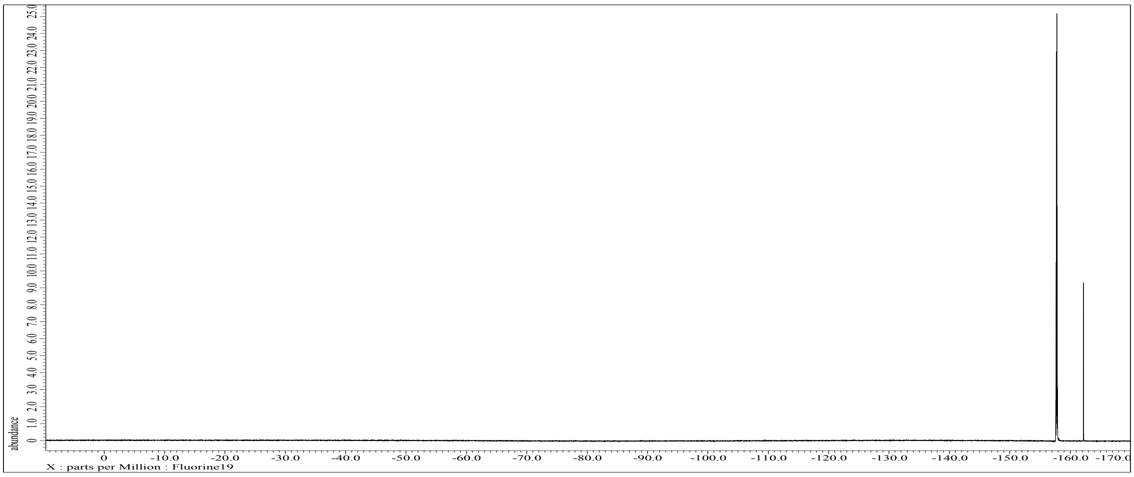

HPLC *optically active* (**5g**)

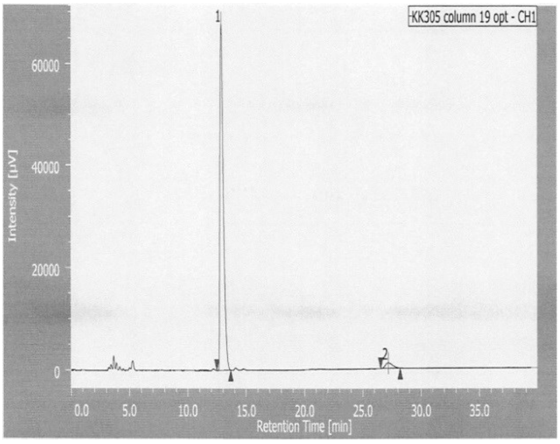

HPLC *racemic* (**5g**)

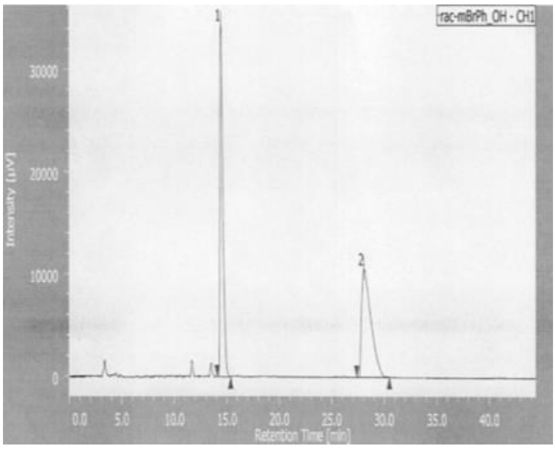

#	ピーク名	CH	tR [min]	面積 [μV·sec]	高さ [μV]	面積%	高さ%	定量値	NTP	分離度	シンメトリー係数	警告
1	Unknown	1	12.842	1384953	67909	96.705	98.407	N/A	9345	17.224	2.019	
2	Unknown	1	27.183	47195	1099	3.295	1.593	N/A	9123	N/A	1.369	

#	ピーク名	CH	tR [min]	面積 [μV·sec]	高さ [μV]	面積%	高さ%	定量値	NTP	分離度	シンメトリー係数	警告
1	Unknown	1	14.408	677931	34626	50.762	76.566	N/A	13134	12.710	2.124	
2	Unknown	1	28.058	657569	10598	49.238	23.434	N/A	4623	N/A	2.369	

^{1}H NMR spectrum (**5h**)

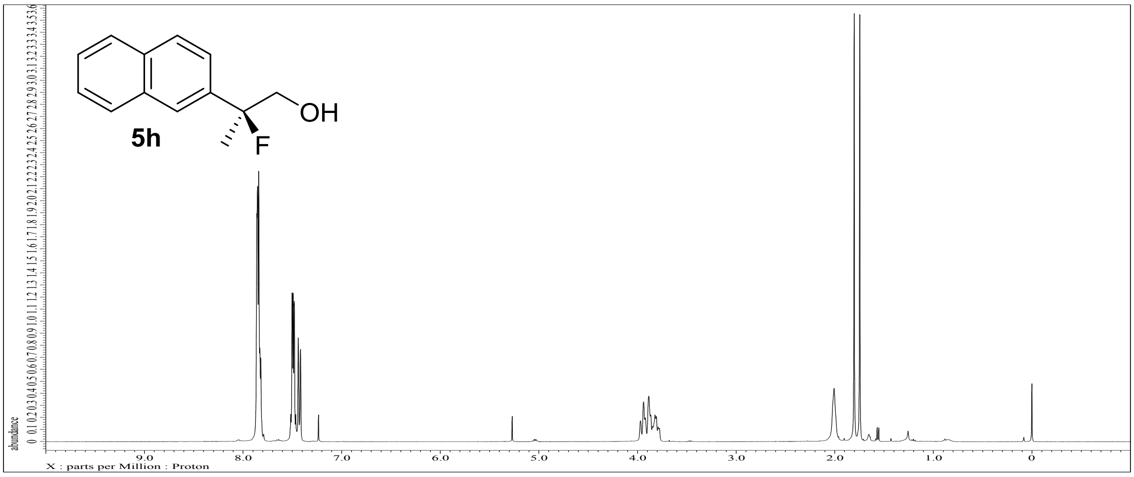

^{13}C NMR spectrum (**5h**)

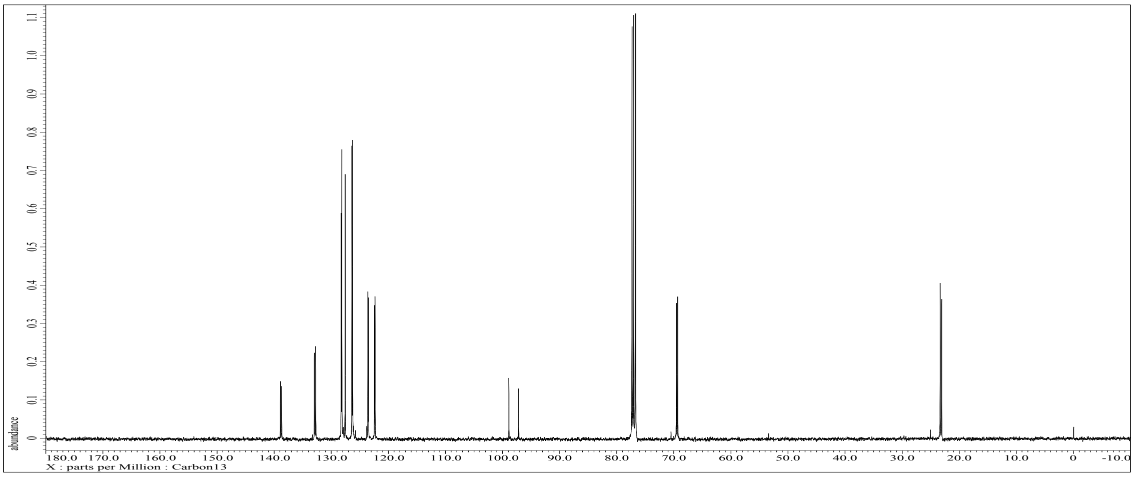

^{19}F NMR spectrum (**5h**)

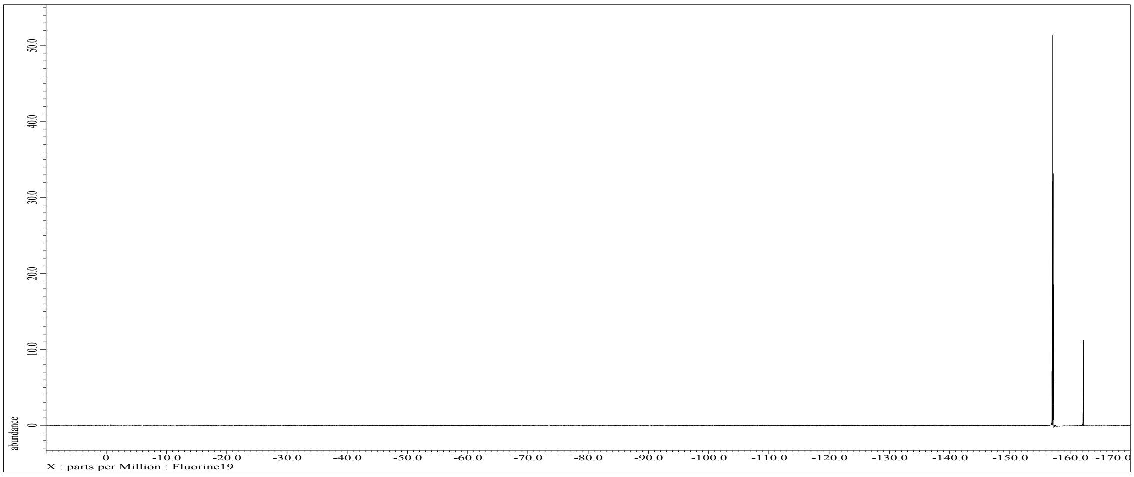

HPLC *optically active* (**5h**)

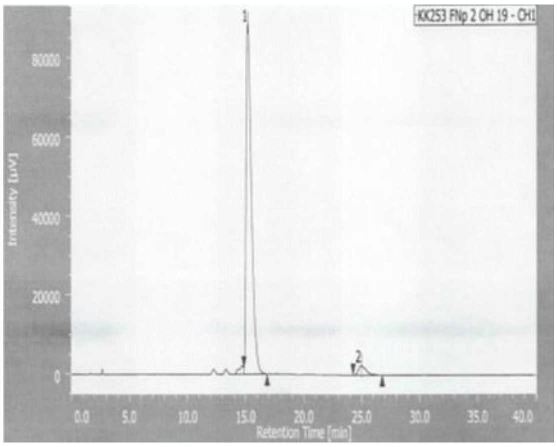

#	ピーク名	CH	tR [min]	面積 [μV·sec]	高さ [μV]	面積%	高さ%	定量値	NTP	分離度	シンメトリー係数	警告
1	Unknown	1	15.200	2802883	88711	96.194	97.434	N/A	5520	9.561	1.990	
2	Unknown	1	24.958	110884	2337	3.806	2.566	N/A	6605	N/A	1.630	

HPLC *racemic* (**5h**)

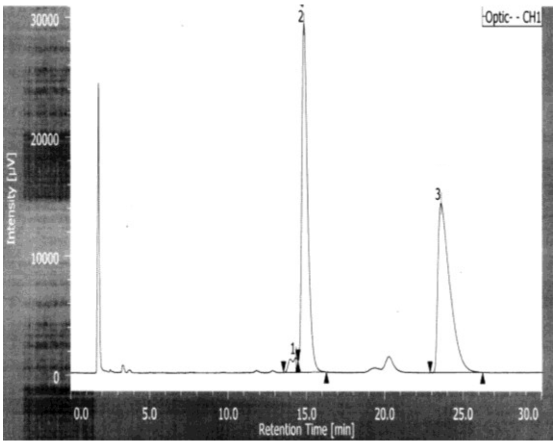

#	ピーク名	CH	tR [min]	面積 [μV·sec]	高さ [μV]	面積%	高さ%	定量値	NTP	分離度	シンメトリー係数	警告
1	Unknown	1	14.325	42104	1225	2.695	2.742	N/A	N/A	N/A	N/A	
2	Unknown	1	14.858	768959	29227	49.221	65.410	N/A	7952	8.666	1.519	
3	Unknown	1	23.608	751197	14230	48.084	31.848	N/A	4834	N/A	2.434	

^{1}H NMR spectrum (**5i**)

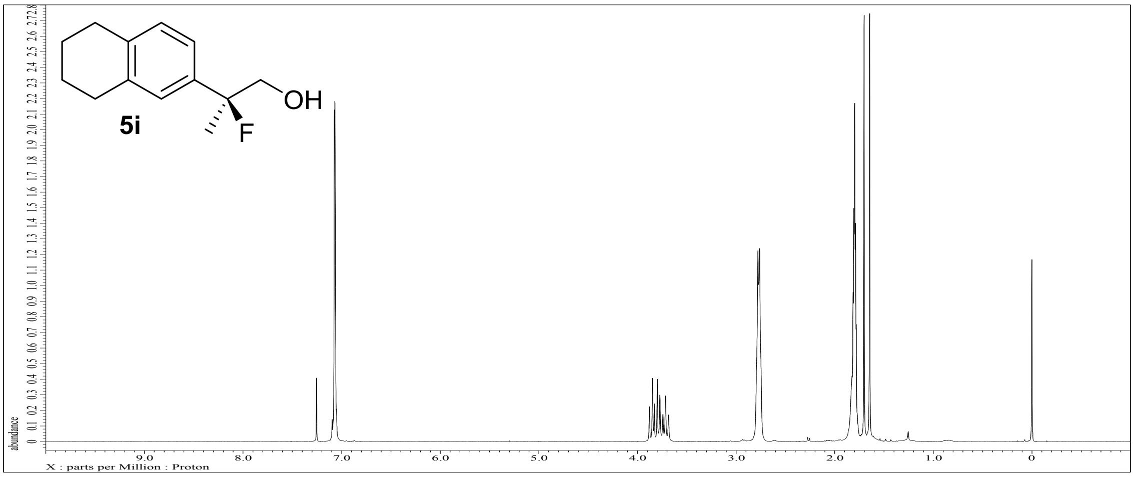

^{13}C NMR spectrum (**5i**)

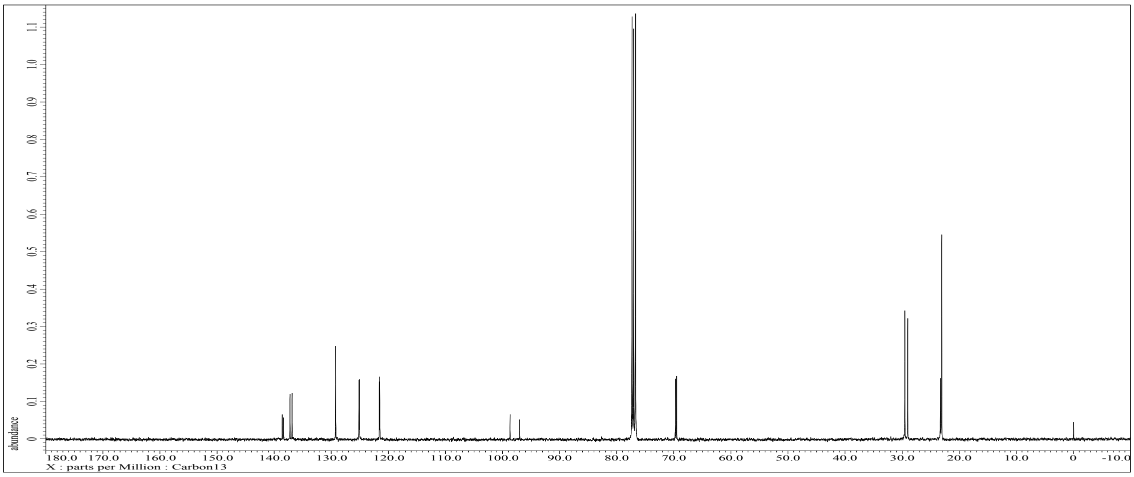

^{19}F NMR spectrum (**5i**)

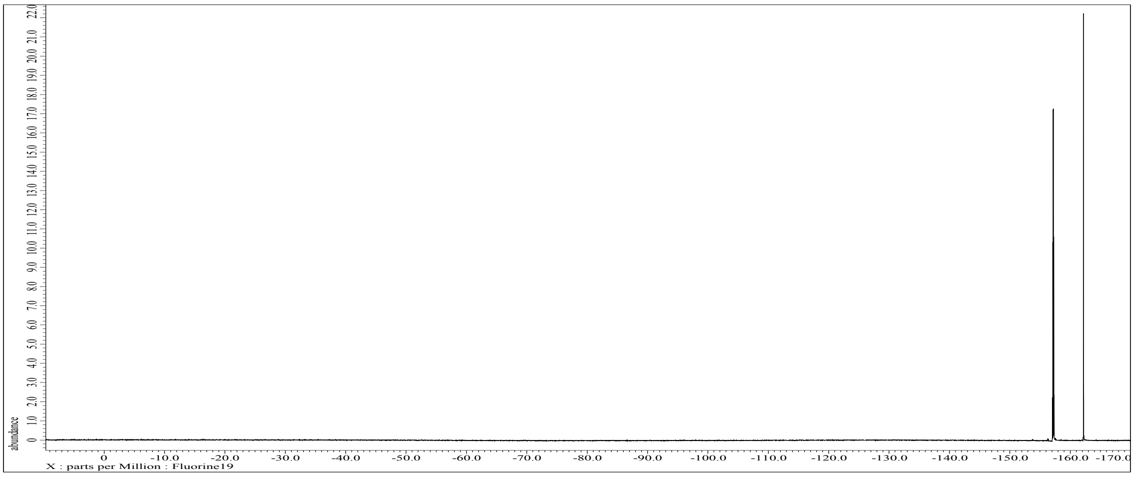

HPLC *optically active* (**5i**)

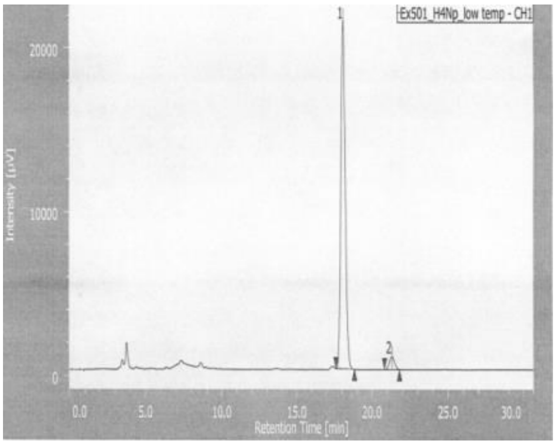

#	ピーク名	CH	tR [min]	面積 [μV·sec]	高さ [μV]	面積%	高さ%	定量値	NTP	分離度	シンメトリー係数	警告
1	Unknown	1	18.058	427388	21238	95.893	96.481	N/A	18855	5.643	1.223	
2	Unknown	1	21.317	18307	775	4.107	3.519	N/A	18208	N/A	1.043	

HPLC *racemic* (**5i**)

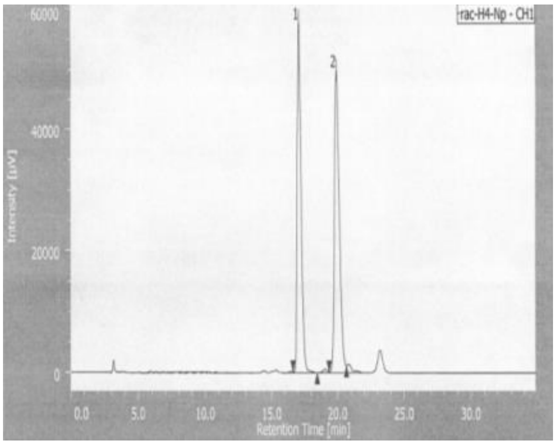

#	ピーク名	CH	tR [min]	面積 [μV·sec]	高さ [μV]	面積%	高さ%	定量値	NTP	分離度	シンメトリー係数	警告
1	Unknown	1	17.050	1195402	57928	48.324	53.755	N/A	16506	4.776	1.532	
2	Unknown	1	19.892	1278300	49836	51.676	46.245	N/A	14432	N/A	1.497	

^{1}H NMR spectrum (**5j**)

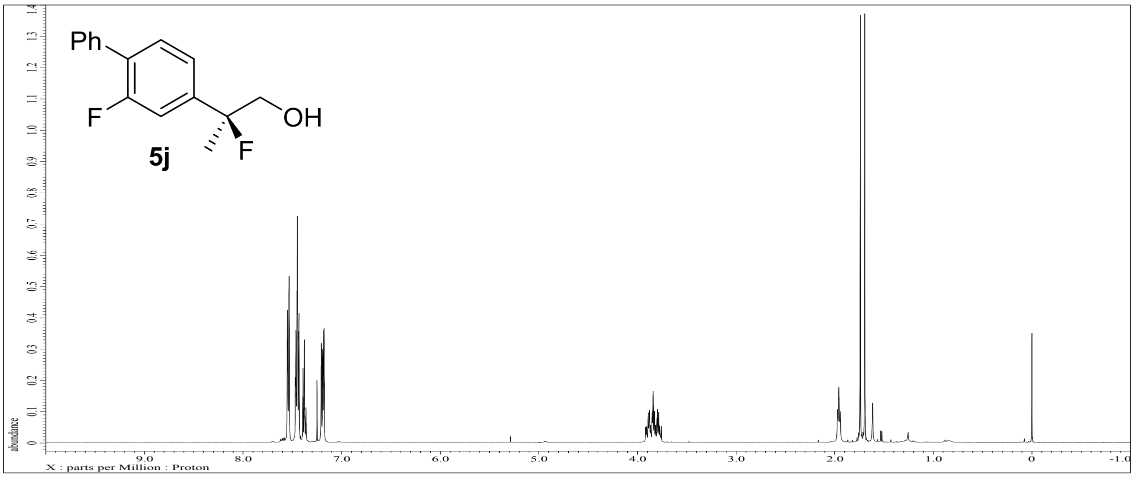

^{13}C NMR spectrum (**5j**)

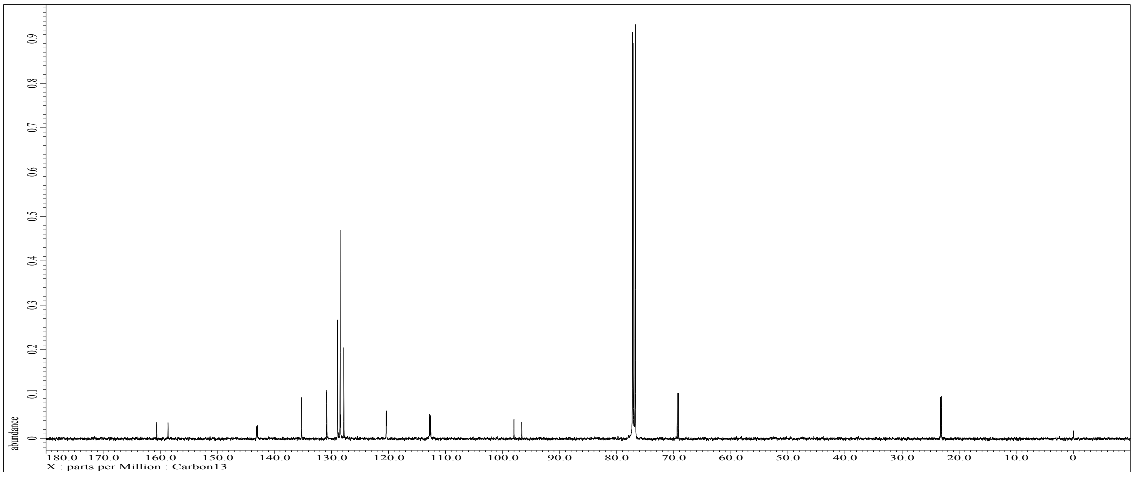

^{19}F NMR spectrum (**5j**)

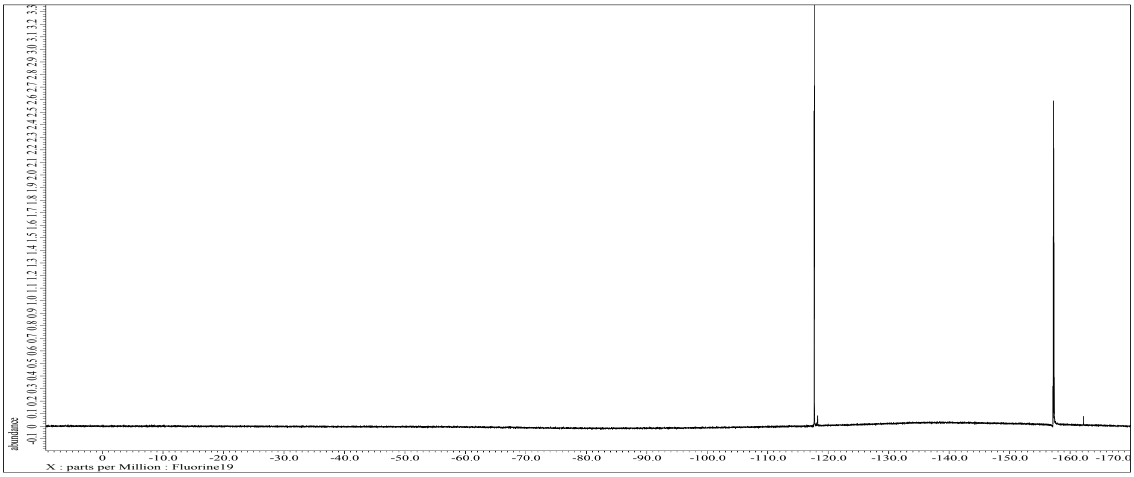

^{1}H NMR spectrum (**18j**)

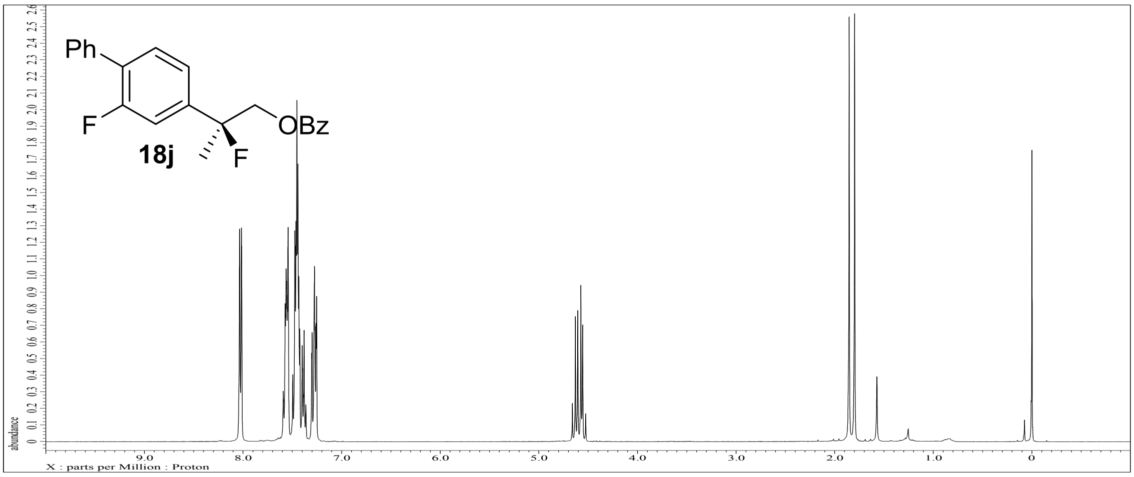

^{13}C NMR spectrum (**18j**)

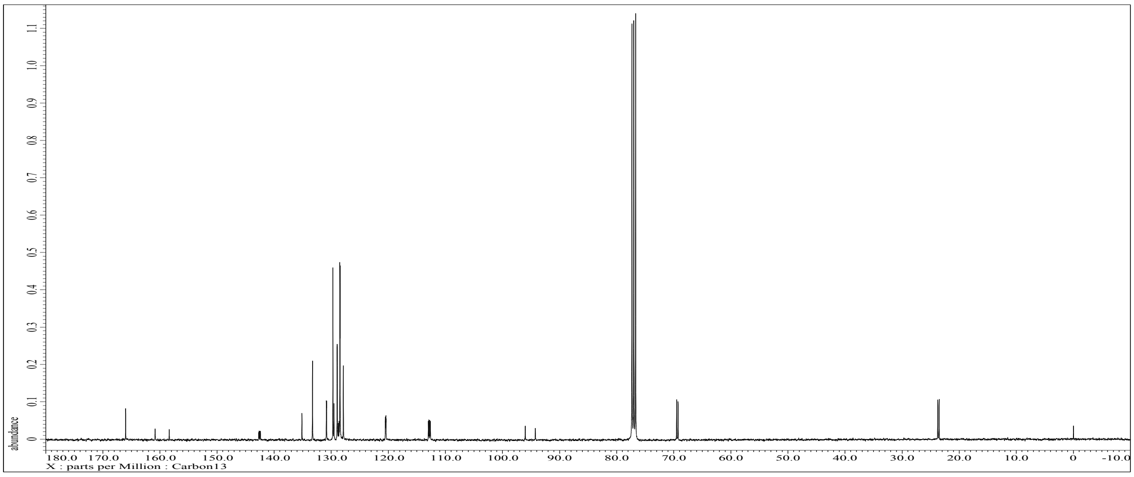

^{19}F NMR spectrum (**18j**)

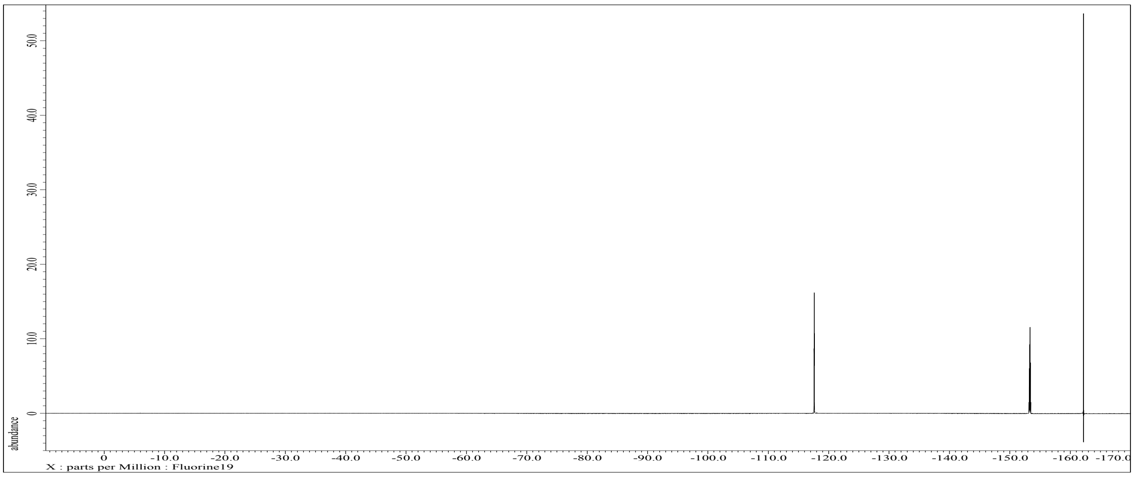

HPLC *optically active* (**18j**)

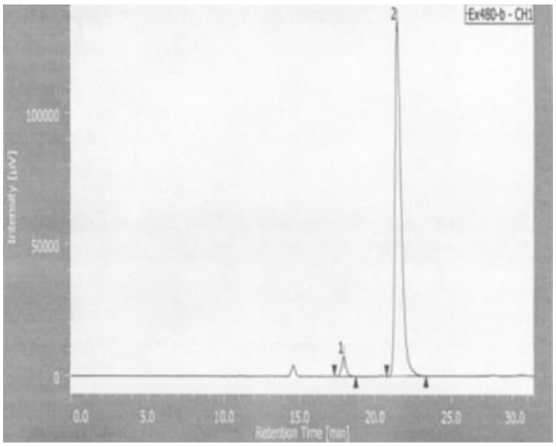

#	ピーク名	CH	tR [min]	面積 [µV·sec]	高さ [µV]	面積%	高さ%	定量値	NTP	分離度	シンメトリー係数	警告
1	Unknown	1	17.933	152379	7482	3.868	5.246	N/A	19574	5.791	1.107	
2	Unknown	1	21.417	3786899	135144	96.132	94.754	N/A	15259	N/A	1.803	

HPLC *racemic* (**18j**)

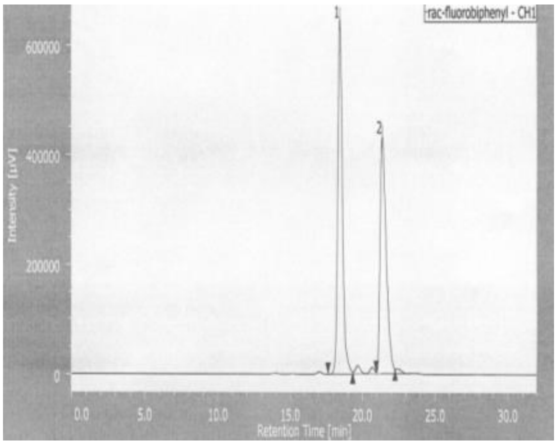

#	ピーク名	CH	tR [min]	面積 [µV·sec]	高さ [µV]	面積%	高さ%	定量値	NTP	分離度	シンメトリー係数	警告
1	Unknown	1	18.433	14558269	645557	54.094	59.990	N/A	16710	4.464	1.310	
2	Unknown	1	21.358	12354566	430553	45.906	40.010	N/A	13197	N/A	1.722	

^{1}H NMR spectrum (**4k**)

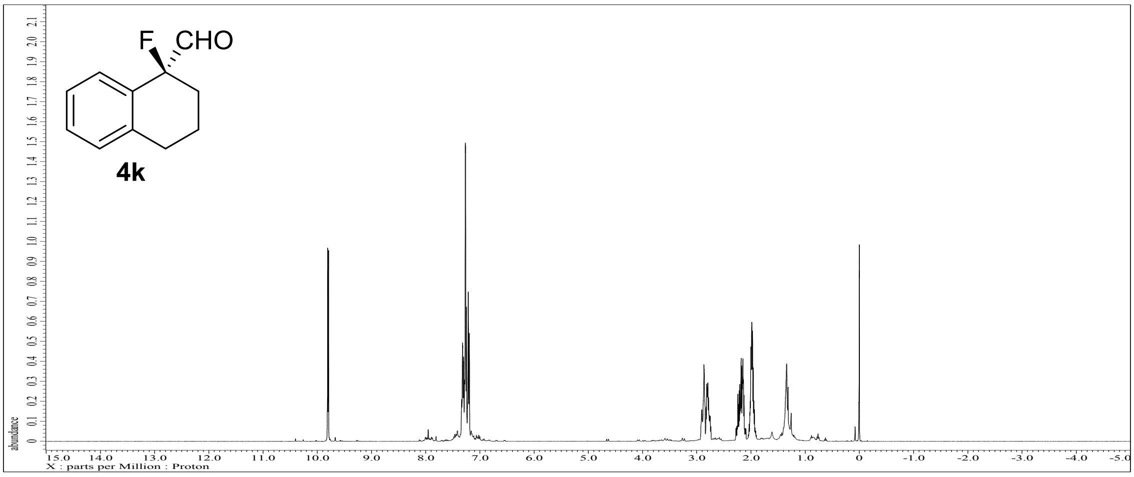

^{13}C NMR spectrum (**4k**)

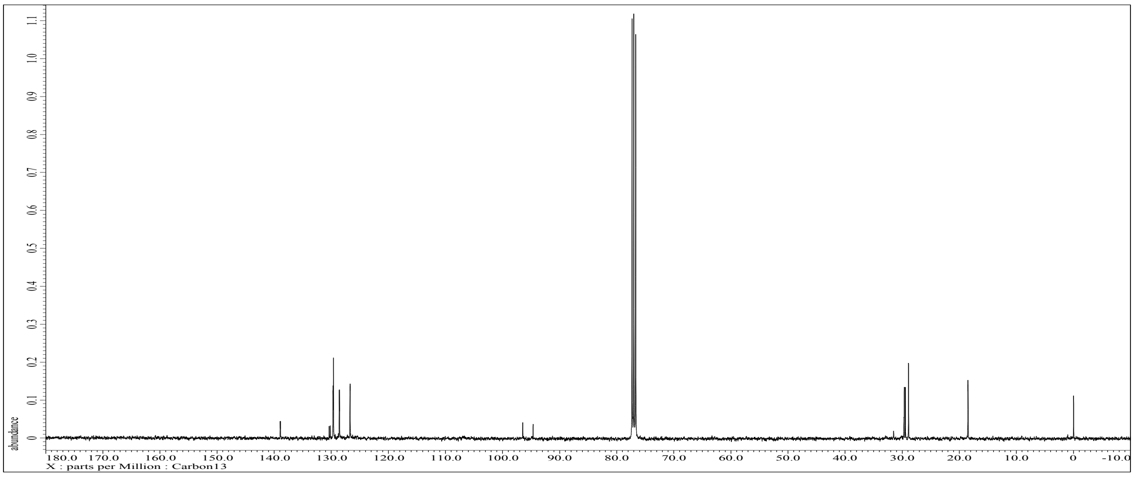

^{19}F NMR spectrum (**4k**)

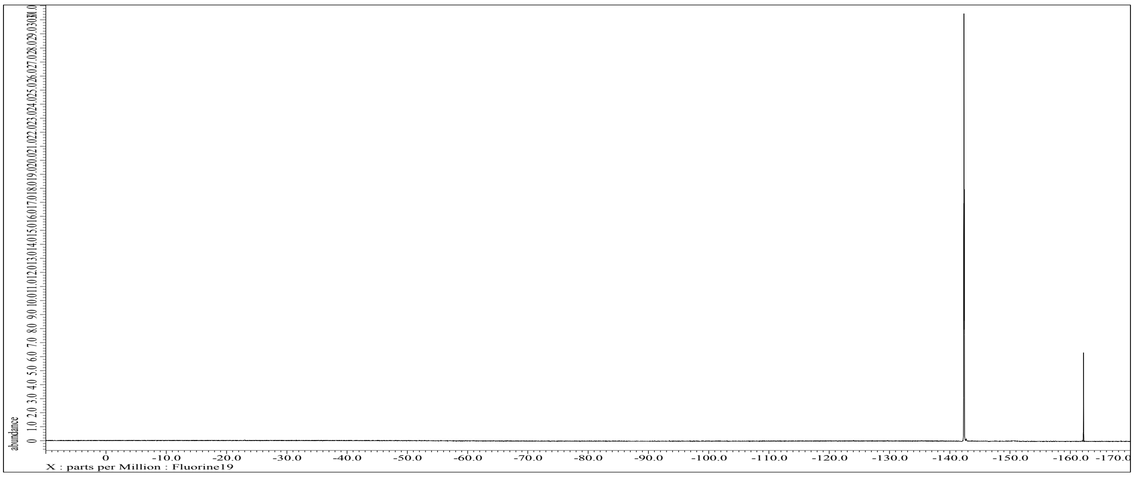

GC *optically active* (**4k**)

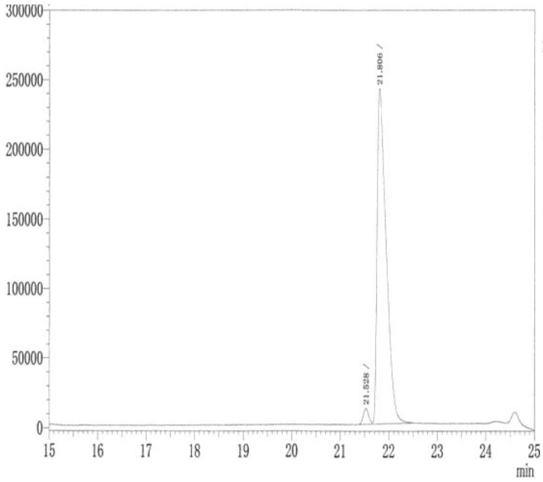

ピーク番号	保持時間	面積	高さ	濃度
1	21.528	84671	11221	2.604
2	21.806	3166809	240423	97.396

GC *racemic* (**4k**)

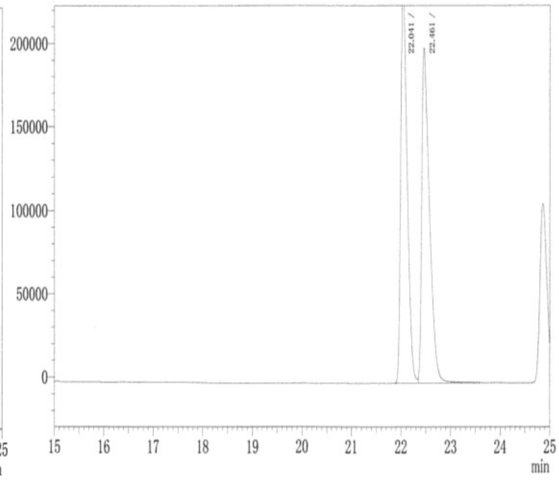

ピーク番号	保持時間	面積	高さ	濃度
1	22.041	2136055	237537	35.901
2	22.461	2162570	200602	36.346

^{1}H NMR spectrum (**5l**)

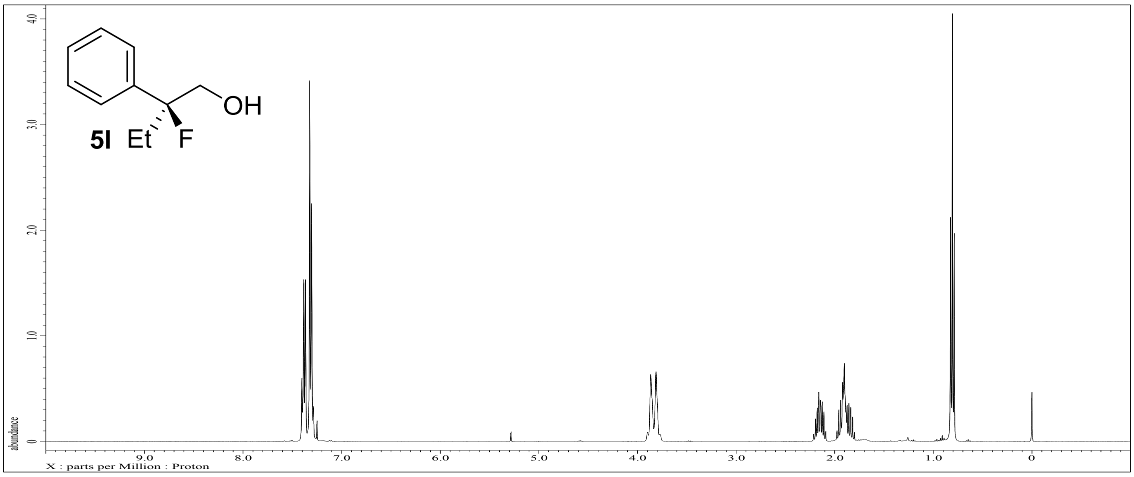

^{13}C NMR spectrum (**5l**)

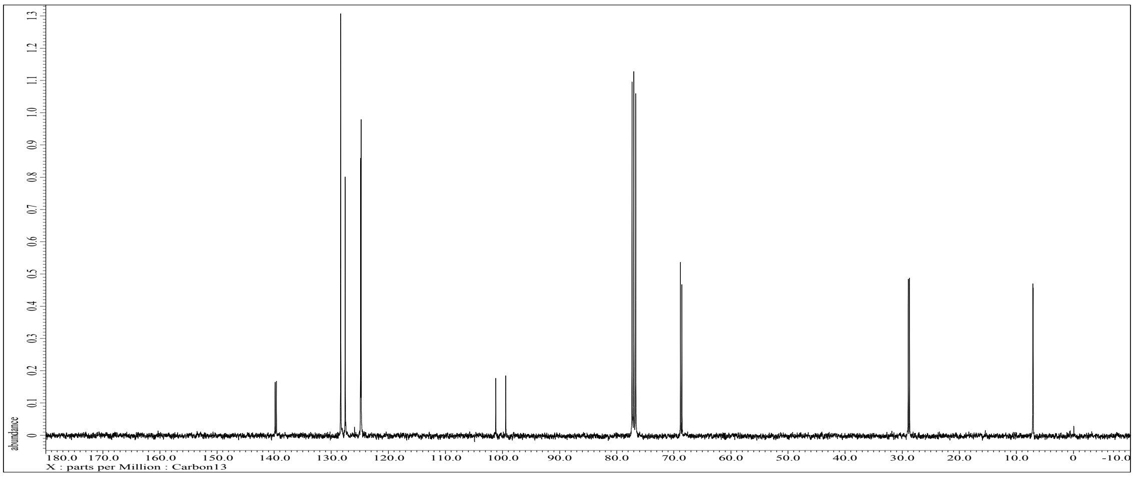

^{19}F NMR spectrum (**5l**)

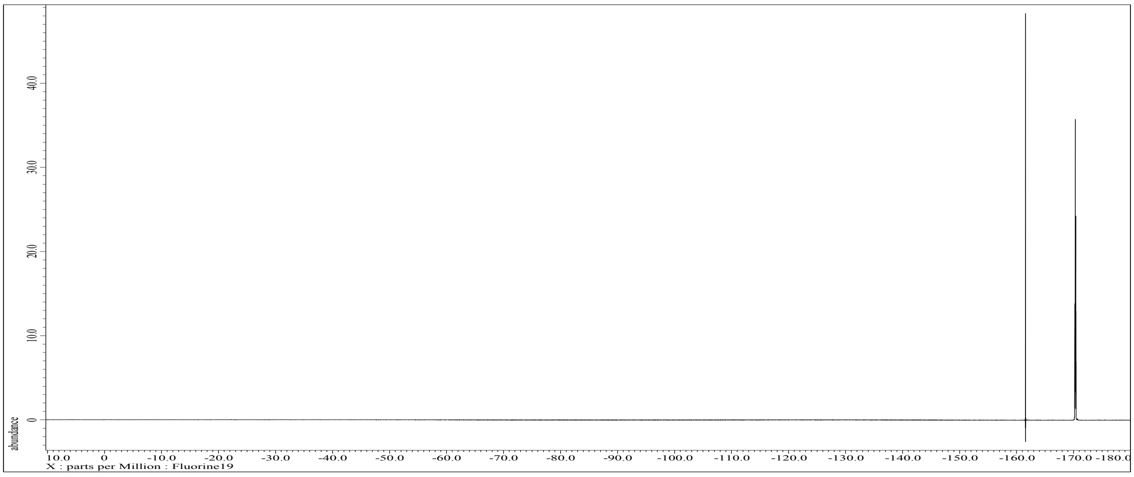

^{1}H NMR spectrum (**18l**)

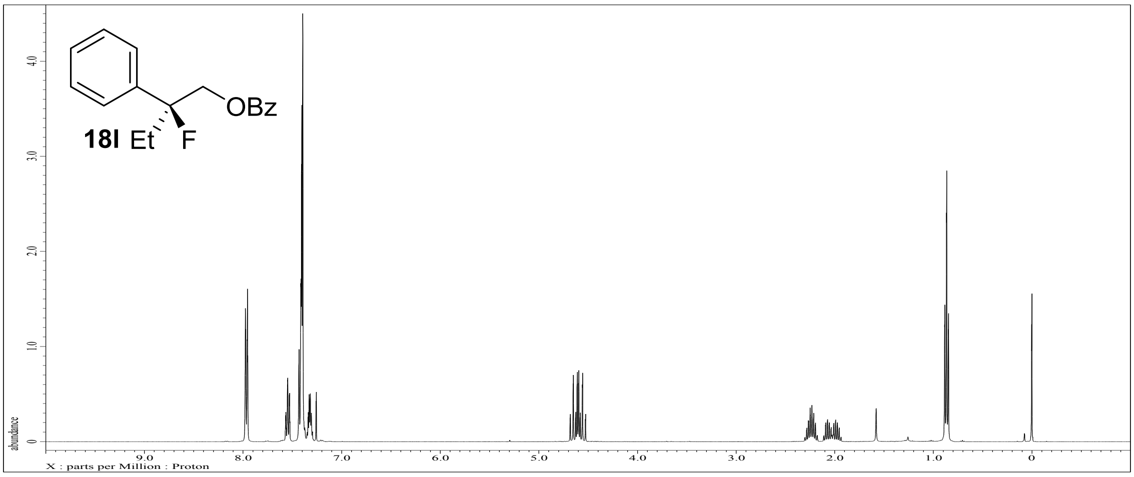

^{13}C NMR spectrum (**18l**)

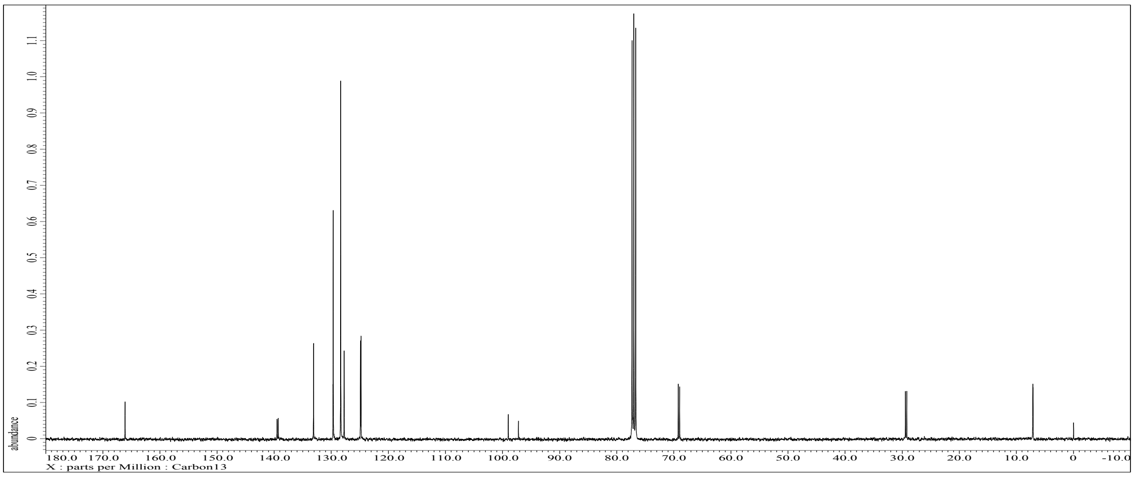

^{19}F NMR spectrum (**18l**)

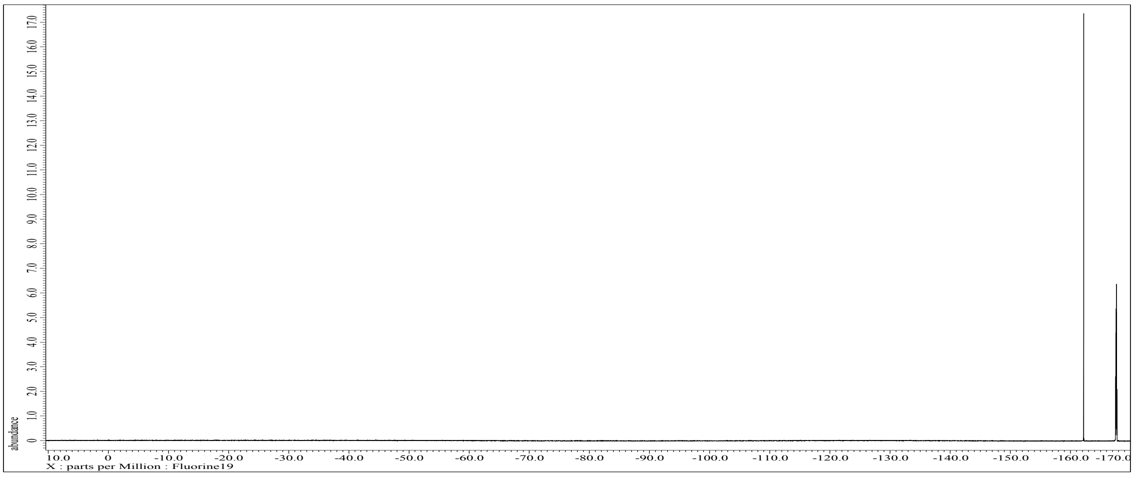

HPLC *optically active* (**18l**)

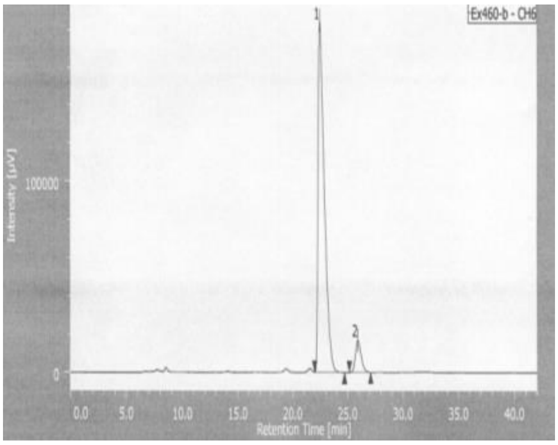

#	ピーク名	CH	tR [min]	面積 [µV·sec]	高さ [µV]	面積%	高さ%	定量値	NTP	分離度	シンメトリー係数	警告
1	Unknown	6	22.440	7164149	184308	92.237	91.627	N/A	8031	3.615	2.486	
2	Unknown	6	25.918	602979	16842	7.763	8.373	N/A	12488	N/A	1.263	

HPLC *racemic* (**18l**)

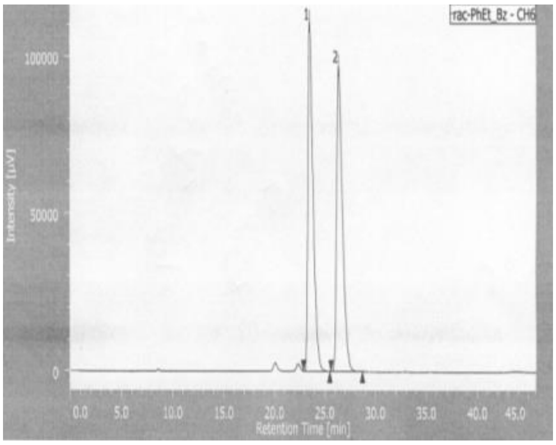

#	ピーク名	CH	tR [min]	面積 [µV·sec]	高さ [µV]	面積%	高さ%	定量値	NTP	分離度	シンメトリー係数	警告
1	Unknown	6	23.455	3927946	111122	50.246	53.397	N/A	10633	2.968	2.068	
2	Unknown	6	26.330	3889555	96984	49.754	46.603	N/A	10399	N/A	1.806	

^{1}H NMR spectrum (**5m**)

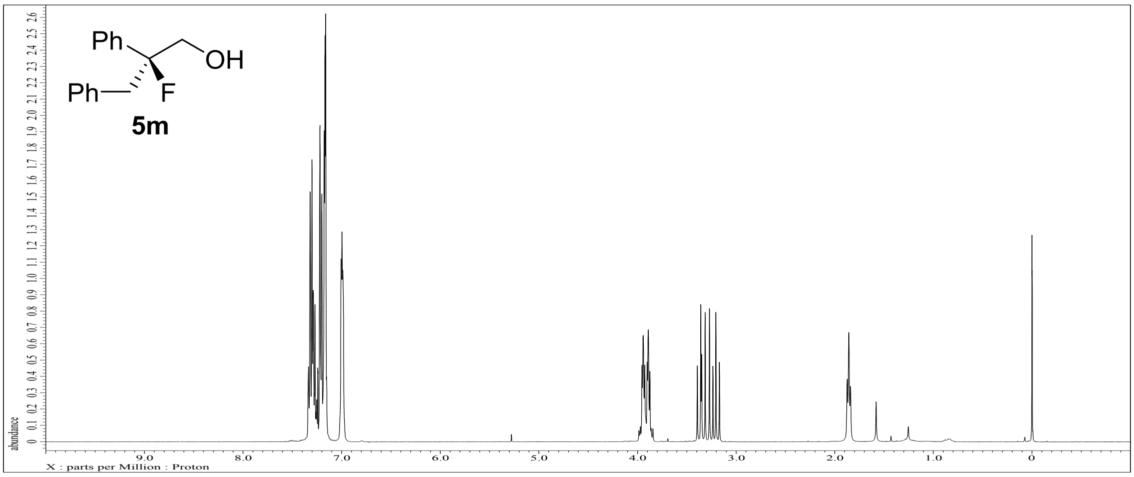

^{13}C NMR spectrum (**5m**)

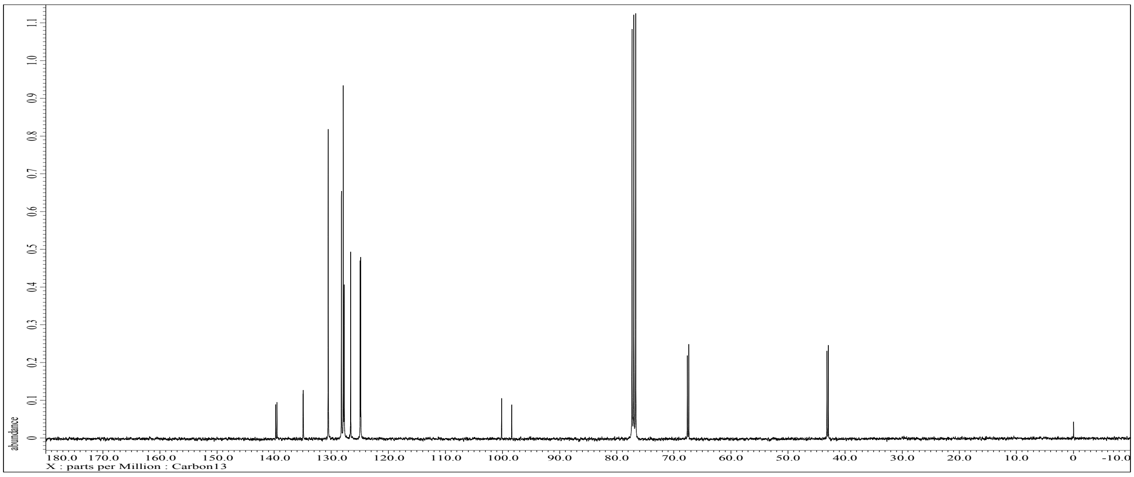

^{19}F NMR spectrum (**5m**)

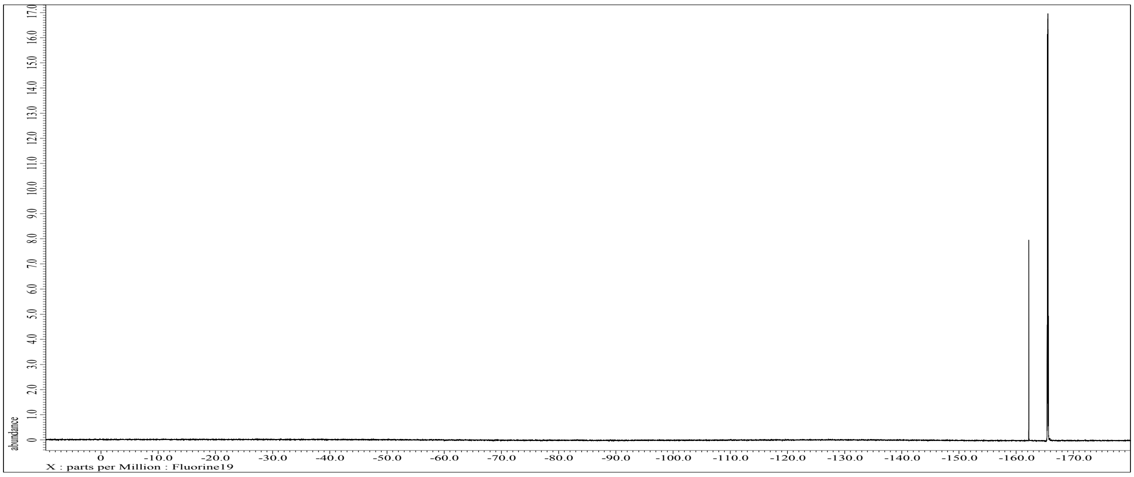

^{1}H NMR spectrum (**18m**)

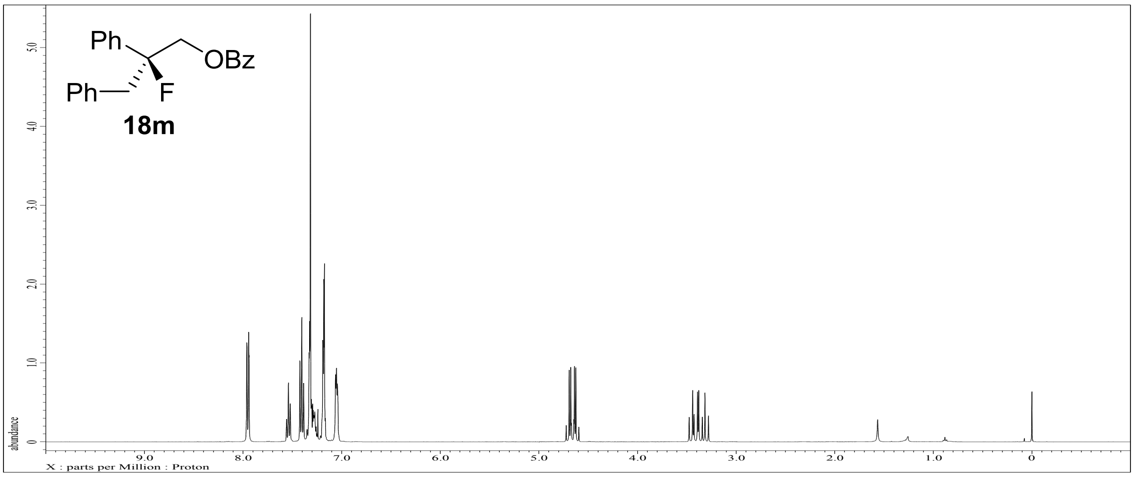

^{13}C NMR spectrum (**18m**)

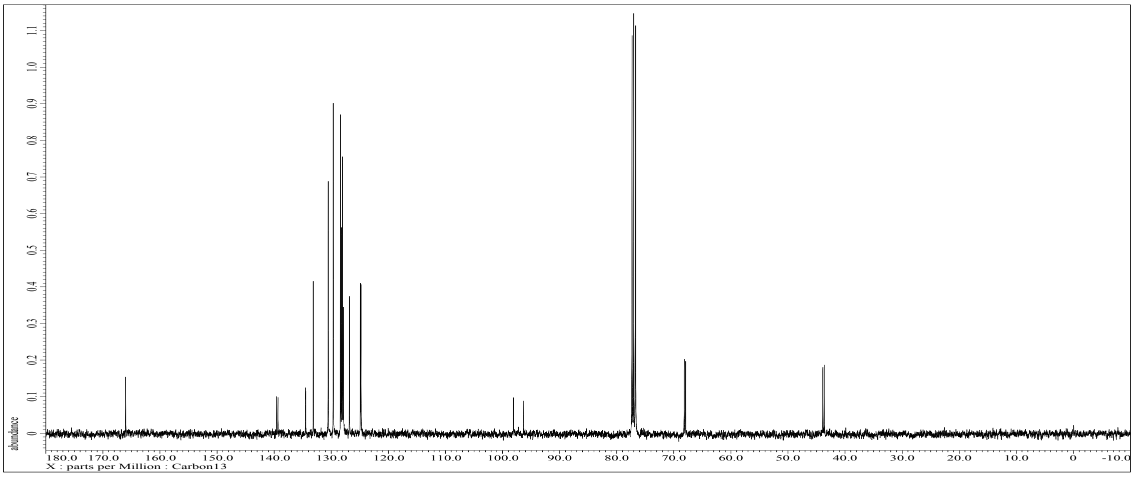

^{19}F NMR spectrum (**18m**)

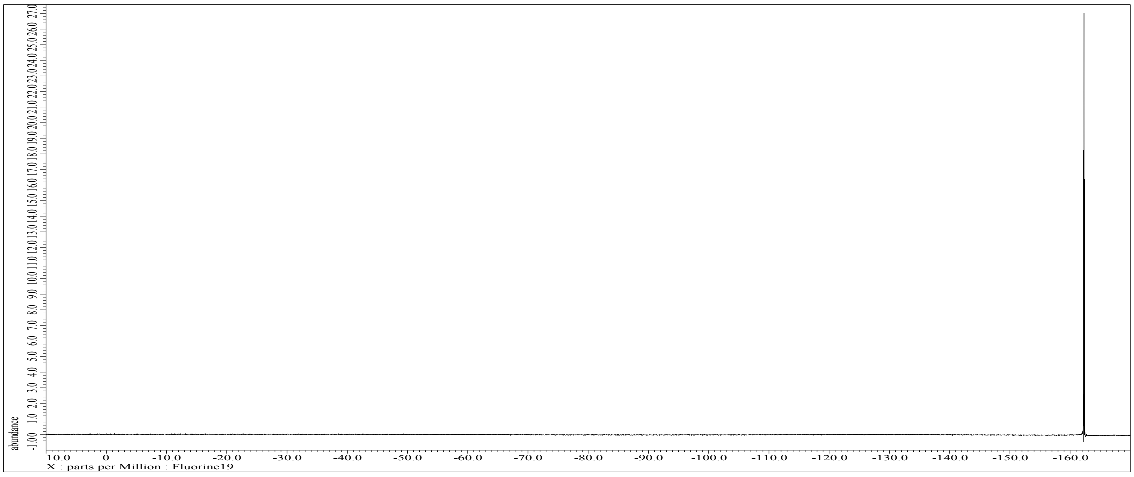

HPLC *optically active* (**18m**)

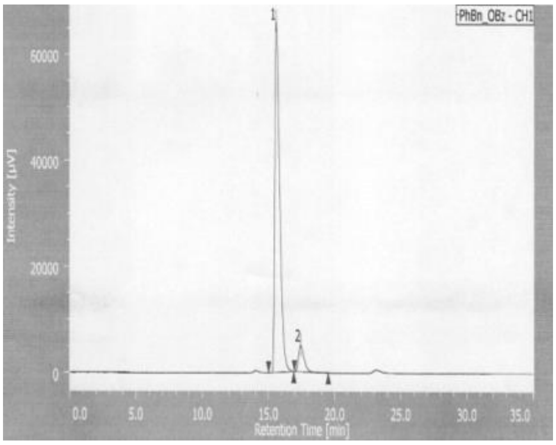

#	ピーク名	CH	tR [min]	面積 [µV·sec]	高さ [µV]	面積%	高さ%	定量値	NTP	分離度	シンメトリー係数	警告
1	Unknown	1	15.592	1706169	66289	92.007	92.947	N/A	8819	2.594	1.958	
2	Unknown	1	17.433	148211	5030	7.993	7.053	N/A	8432	N/A	1.473	

HPLC *racemic* (**18m**)

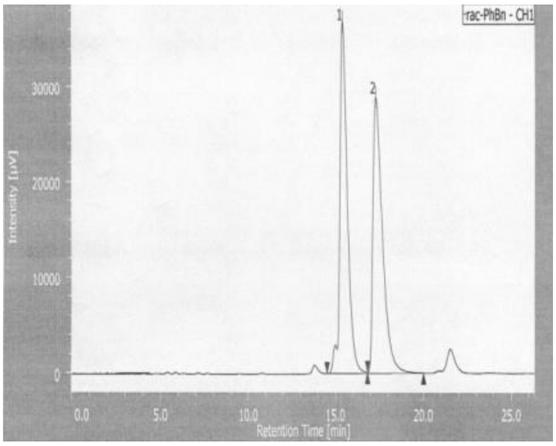

#	ピーク名	CH	tR [min]	面積 [µV·sec]	高さ [µV]	面積%	高さ%	定量値	NTP	分離度	シンメトリー係数	警告
1	Unknown	1	15.392	1104539	36778	50.382	56.052	N/A	7174	2.286	1.221	
2	Unknown	1	17.300	1087773	28836	49.618	43.948	N/A	5339	N/A	2.161	

^{1}H NMR spectrum (**5n**)

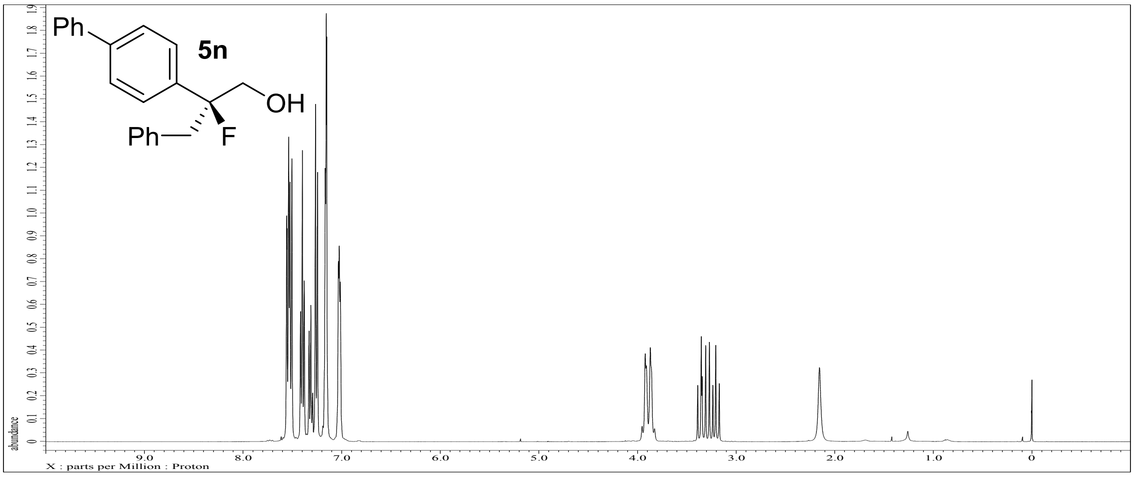

^{13}C NMR spectrum (**5n**)

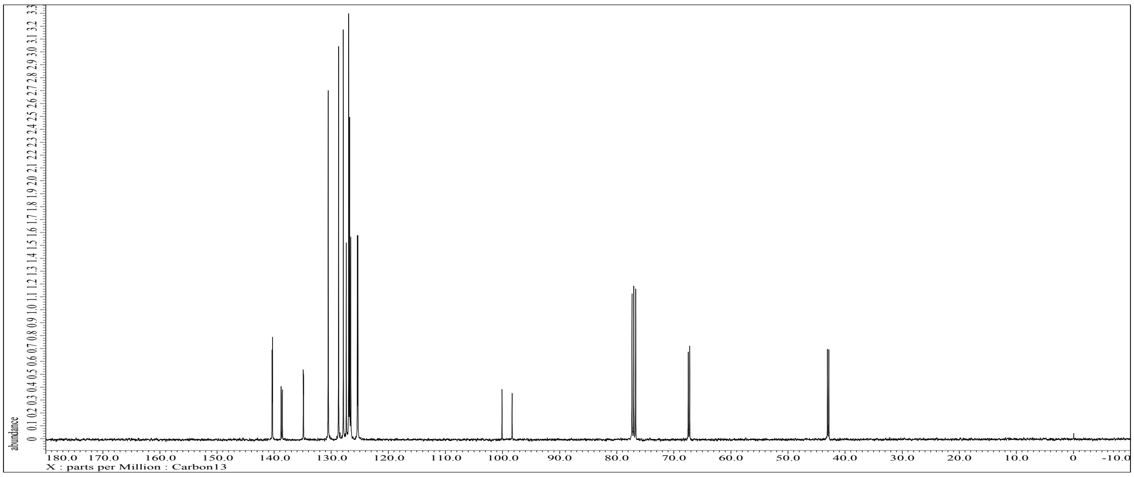

^{19}F NMR spectrum (**5n**)

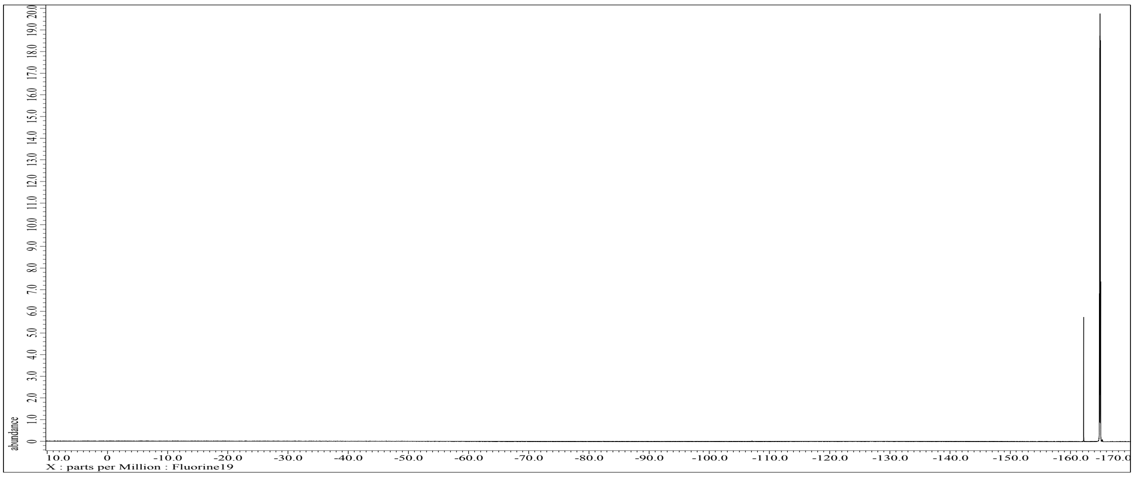

HPLC *optically active* (**5n**)

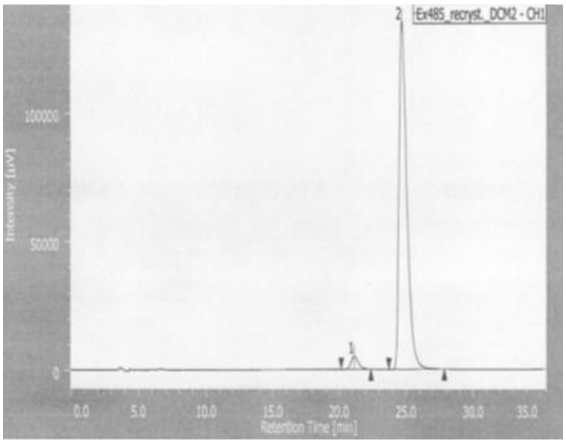

#	ピーク名	CH	tR [min]	面積 [µV·sec]	高さ [µV]	面積%	高さ%	定量値	NTP	分離度	シンメトリー係数	警告
1	Unknown	1	21.142	189586	4956	3.358	3.531	N/A	7276	3.527	1.330	
2	Unknown	1	24.692	5456613	135403	96.642	96.469	N/A	9250	N/A	1.725	

HPLC *racemic* (**5n**)

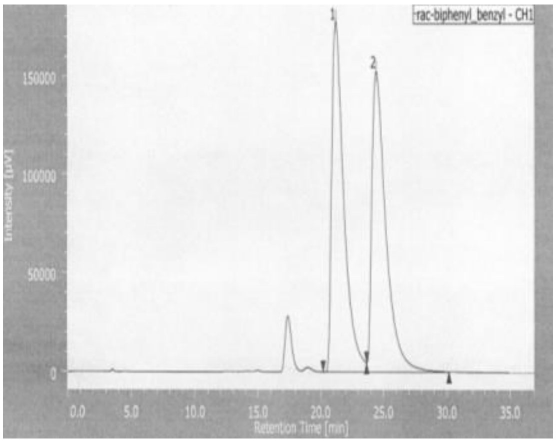

#	ピーク名	CH	tR [min]	面積 [µV·sec]	高さ [µV]	面積%	高さ%	定量値	NTP	分離度	シンメトリー係数	警告
1	Unknown	1	21.192	11963810	177645	50.698	53.809	N/A	2560	1.822	2.228	
2	Unknown	1	24.417	11634478	152493	49.302	46.191	N/A	2717	N/A	2.247	

^{1}H NMR spectrum (**5o**)

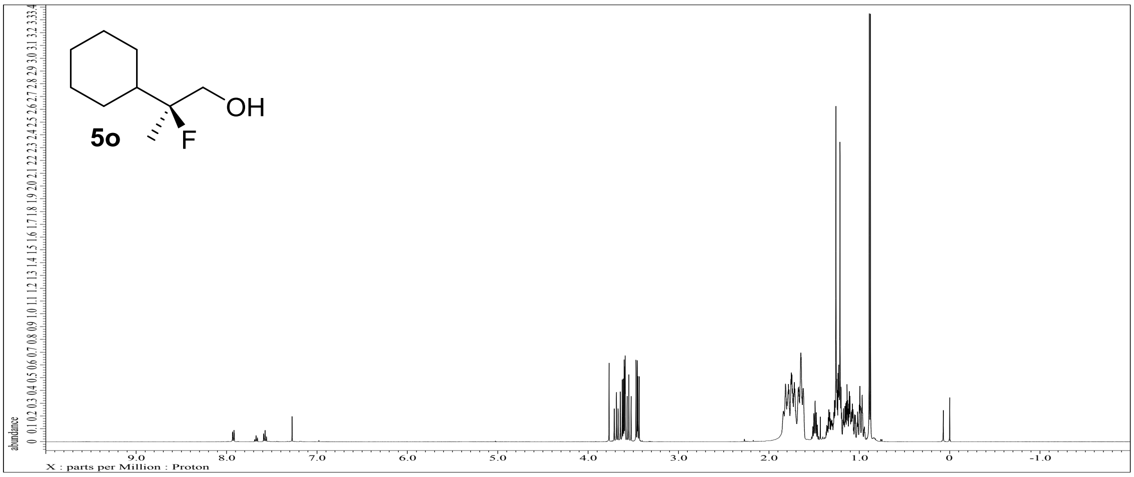

^{13}C NMR spectrum (**5o**)

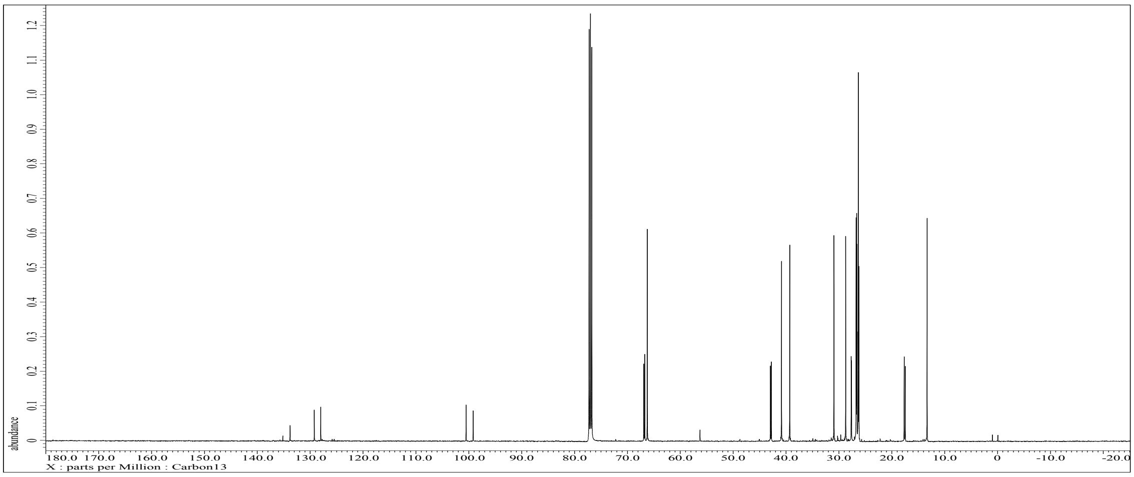

^{19}F NMR spectrum (**5o**)

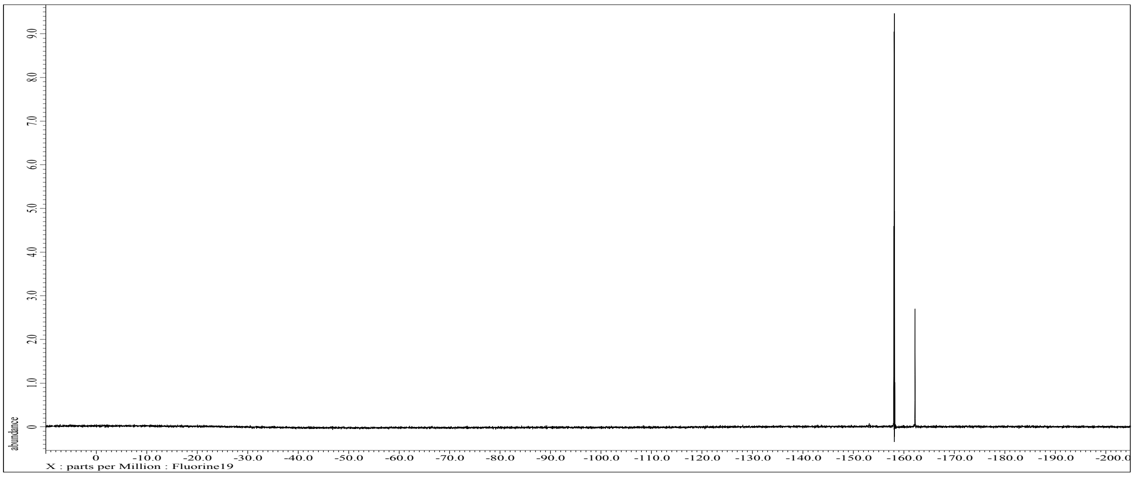

GC *optically active* (**5o**)

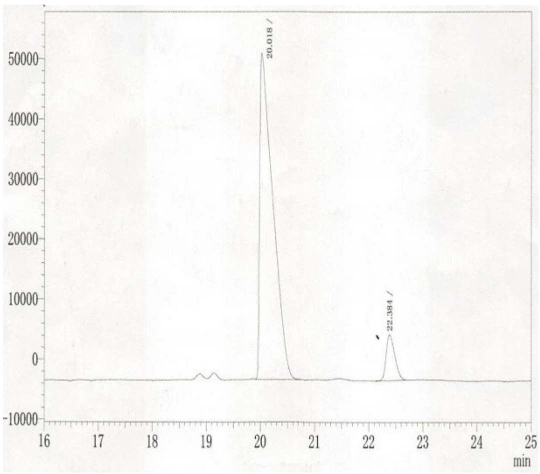

ピーク番号	保持時間	面積	高さ	濃度
1	20.018	913096	54304	91.525
2	22.384	84547	7682	8.475

GC *racemic* (**5o**)

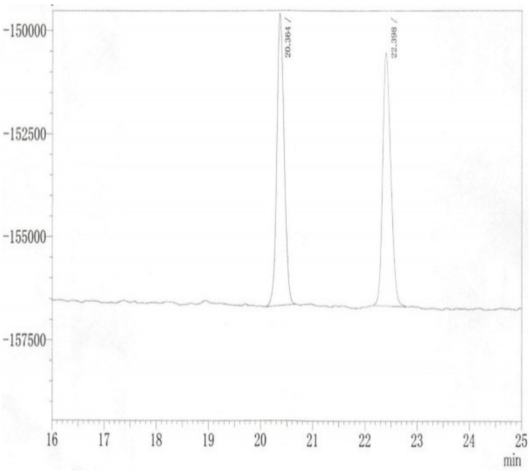

ピーク番号	保持時間	面積	高さ	濃度
1	20.364	68701	7063	50.844
2	22.398	66419	6135	49.156

^{1}H NMR spectrum (**5p**)

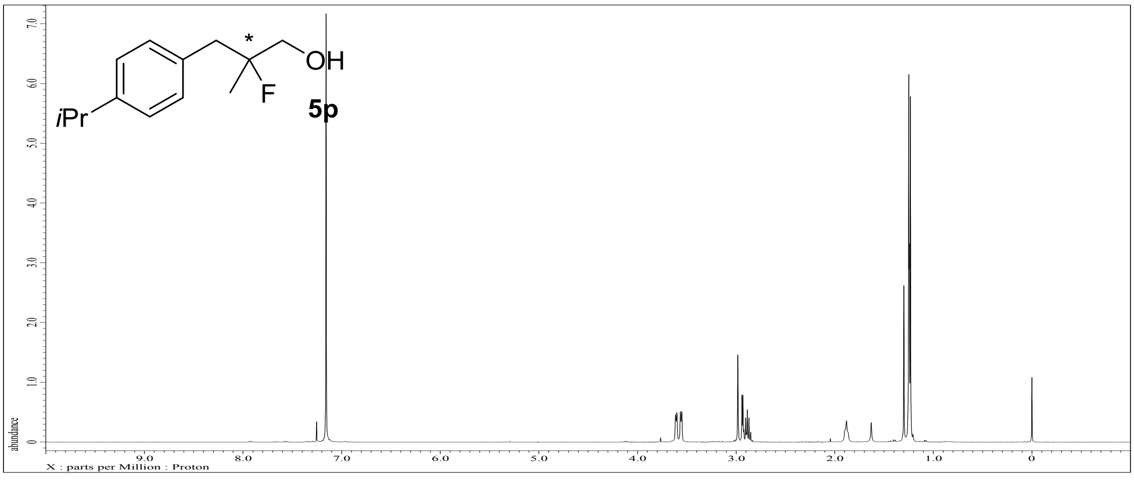

^{13}C NMR spectrum (**5p**)

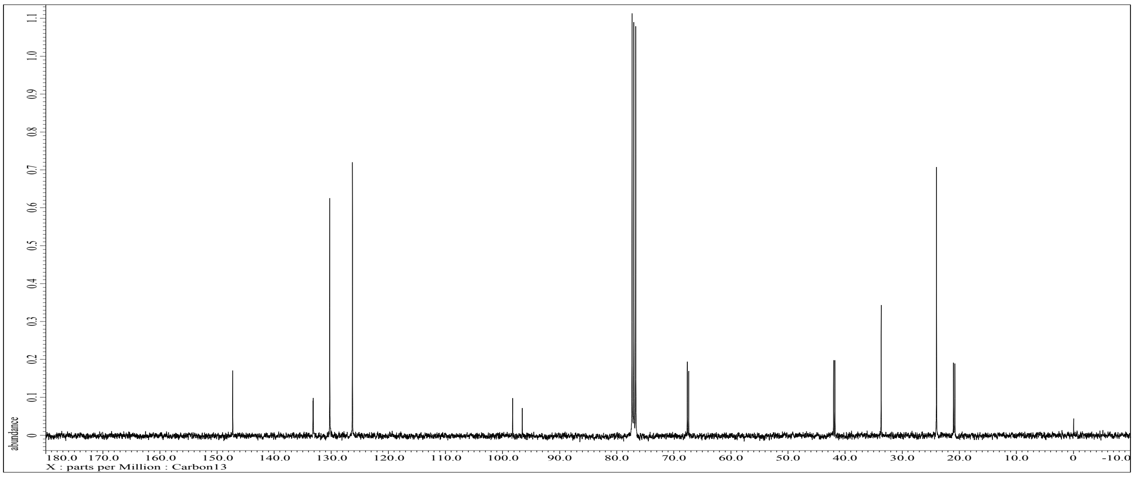

^{19}F NMR spectrum (**5p**)

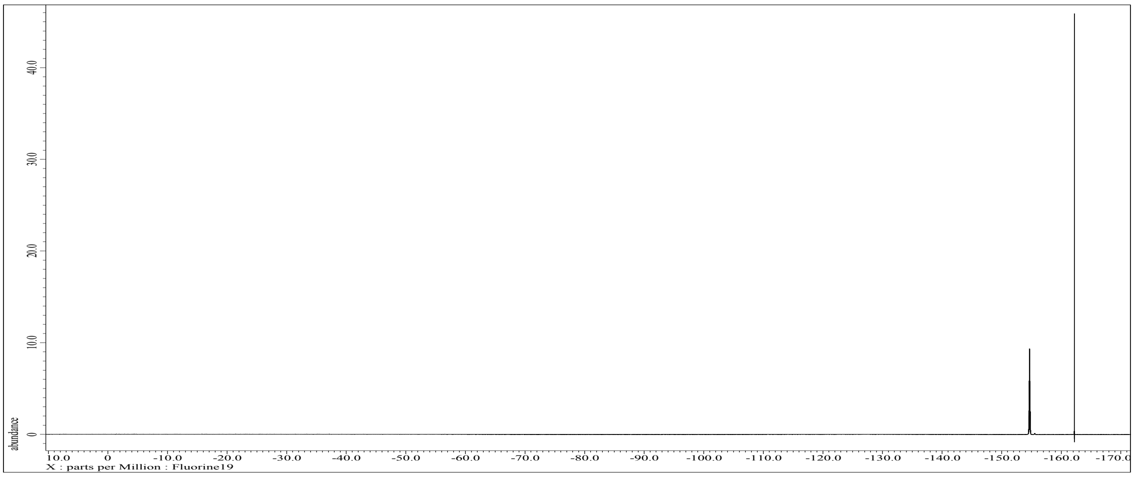

^{1}H NMR spectrum (**18p**)

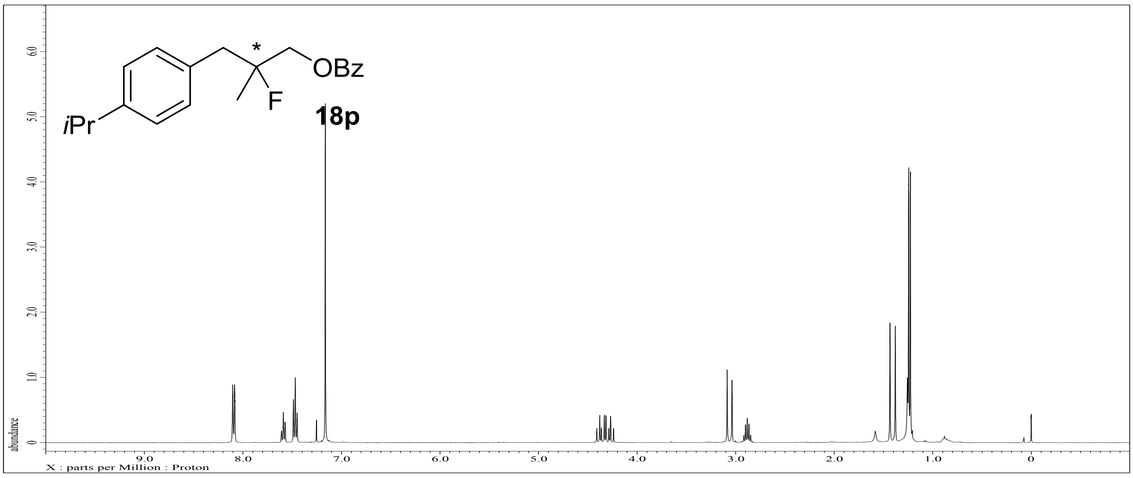

^{13}C NMR spectrum (**18p**)

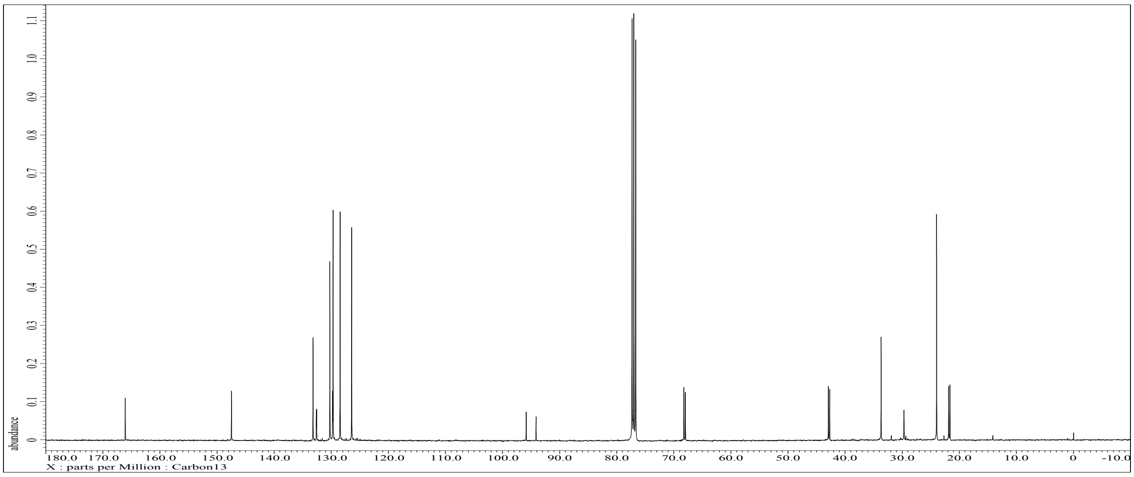

^{19}F NMR spectrum (**18p**)

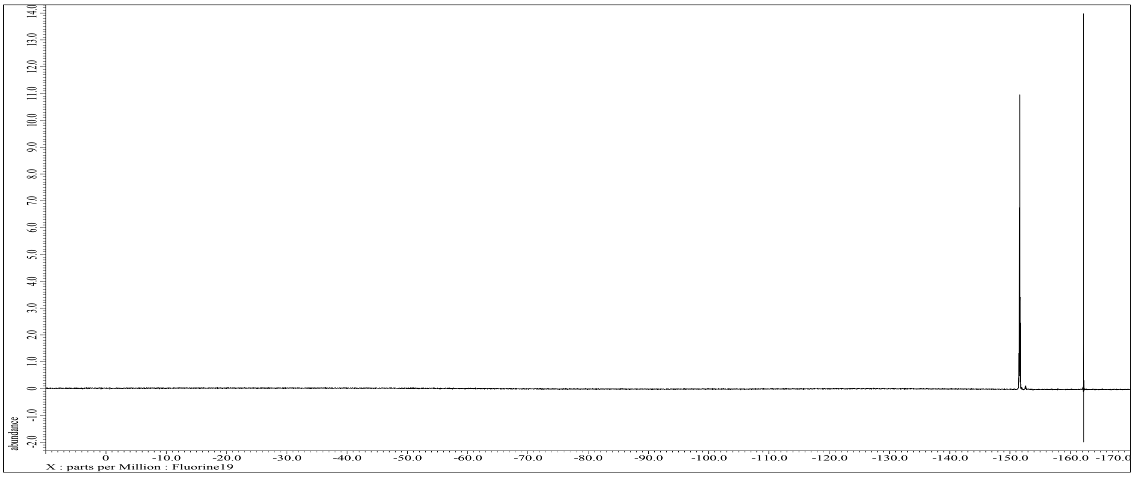

HPLC *optically active* (**18p**)

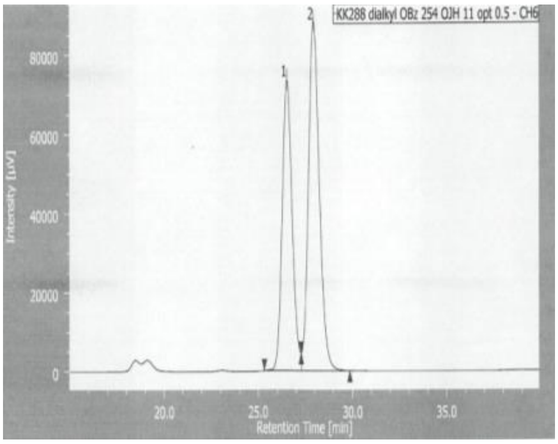

#	ピーク名	CH	tR [min]	面積 [µV·sec]	高さ [µV]	面積%	高さ%	定量値	NTP	分離度	シンメトリー係数	警告
1	Unknown	6	26.515	2690899	73434	43.149	45.386	N/A	12340	1.435	N/A	
2	Unknown	6	27.937	3545454	88366	56.851	54.614	N/A	11722	N/A	N/A	

HPLC *racemic* (**18p**)

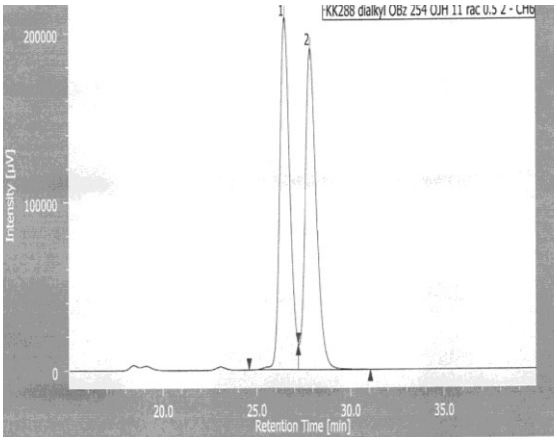

#	ピーク名	CH	tR [min]	面積 [µV·sec]	高さ [µV]	面積%	高さ%	定量値	NTP	分離度	シンメトリー係数	警告
1	Unknown	6	26.487	7724341	208617	49.548	52.320	N/A	12089	1.367	N/A	
2	Unknown	6	27.862	7865422	190115	50.452	47.680	N/A	11202	N/A	N/A	

^{1}H NMR spectrum (**8b**)

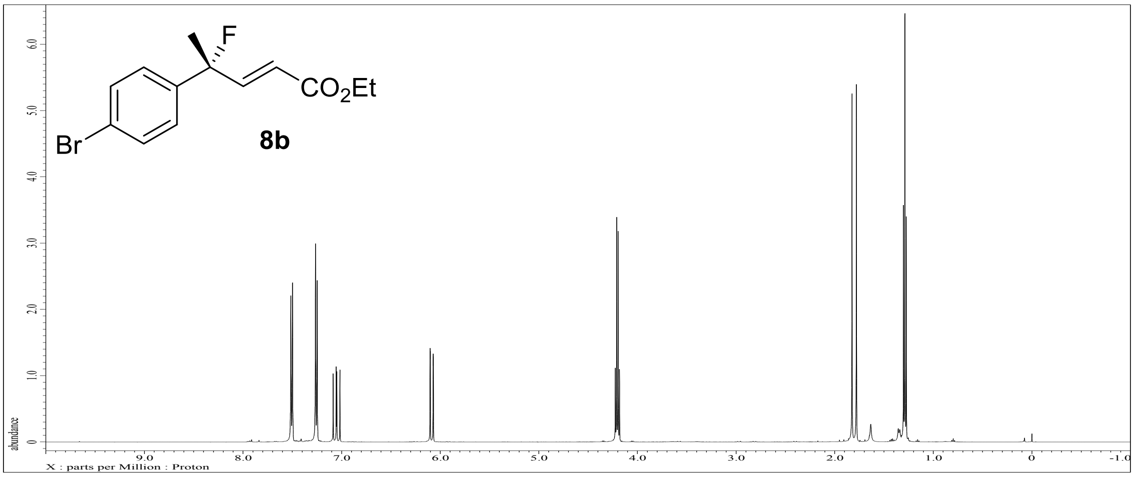

^{13}C NMR spectrum (**8b**)

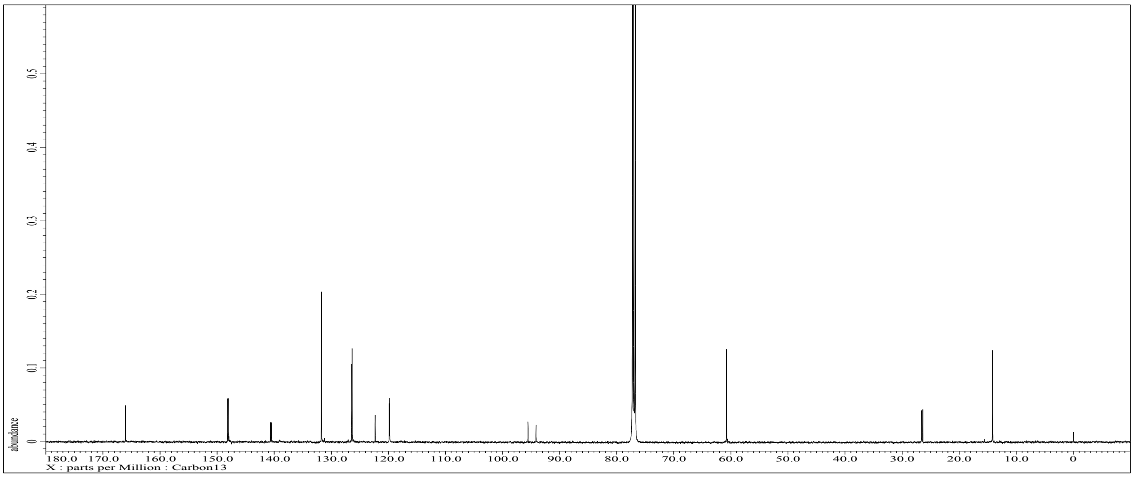

^{19}F NMR spectrum (**8b**)

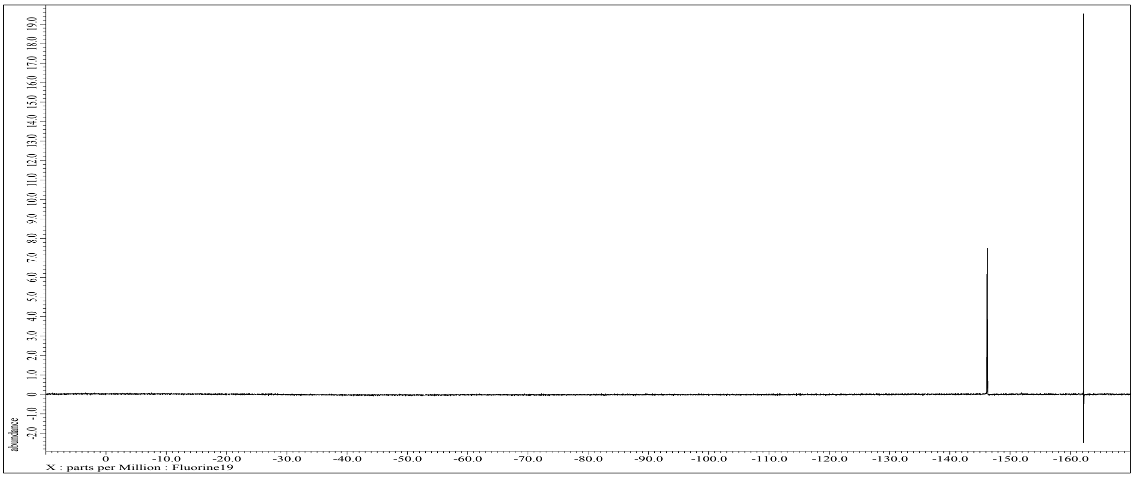

^{1}H NMR spectrum (**8h**)

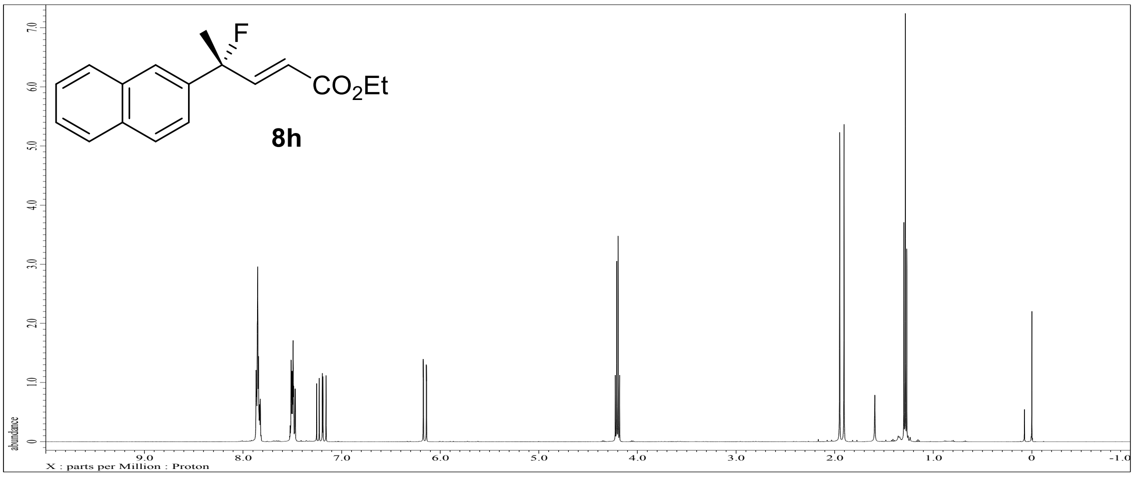

^{13}C NMR spectrum (**8h**)

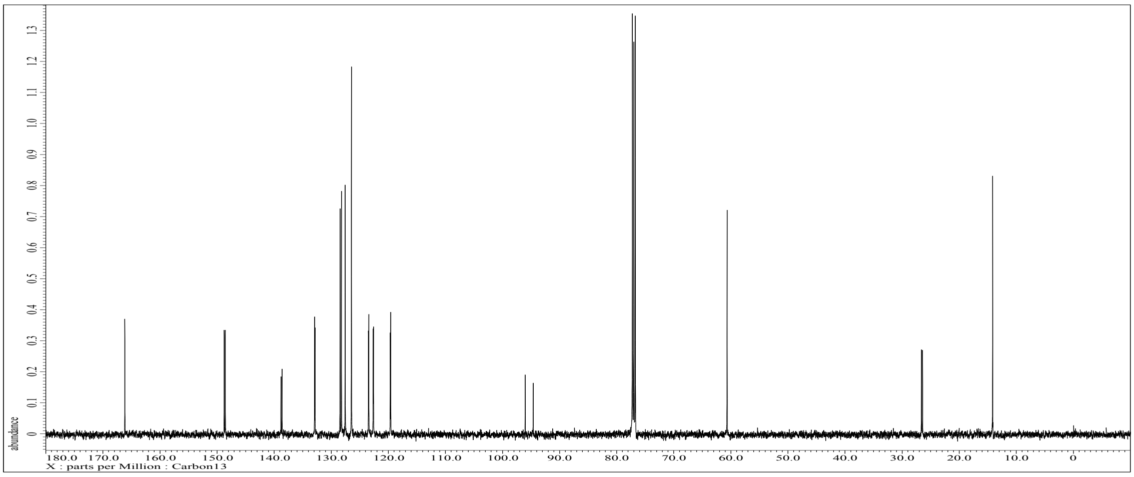

^{19}F NMR spectrum (**8h**)

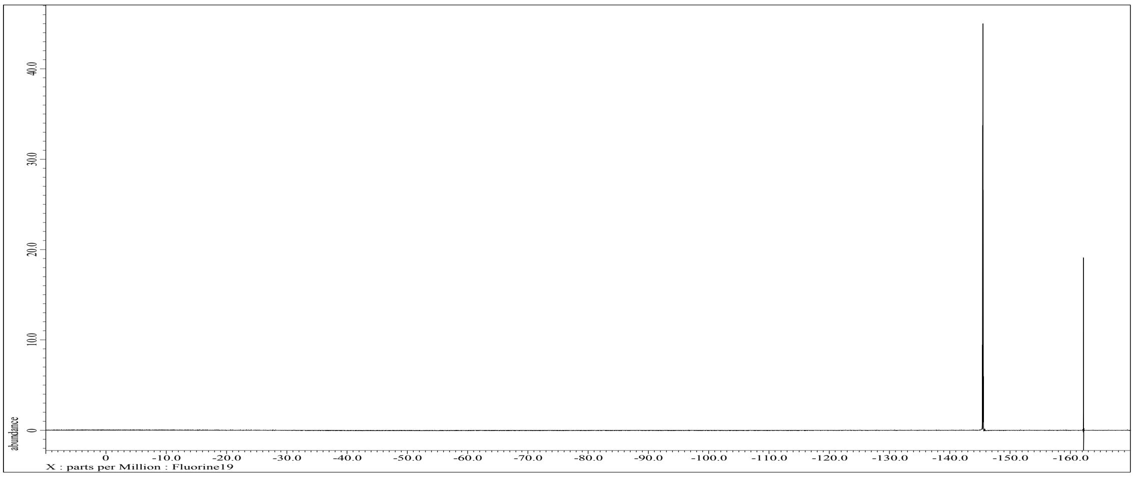

^{1}H NMR spectrum (**9**)

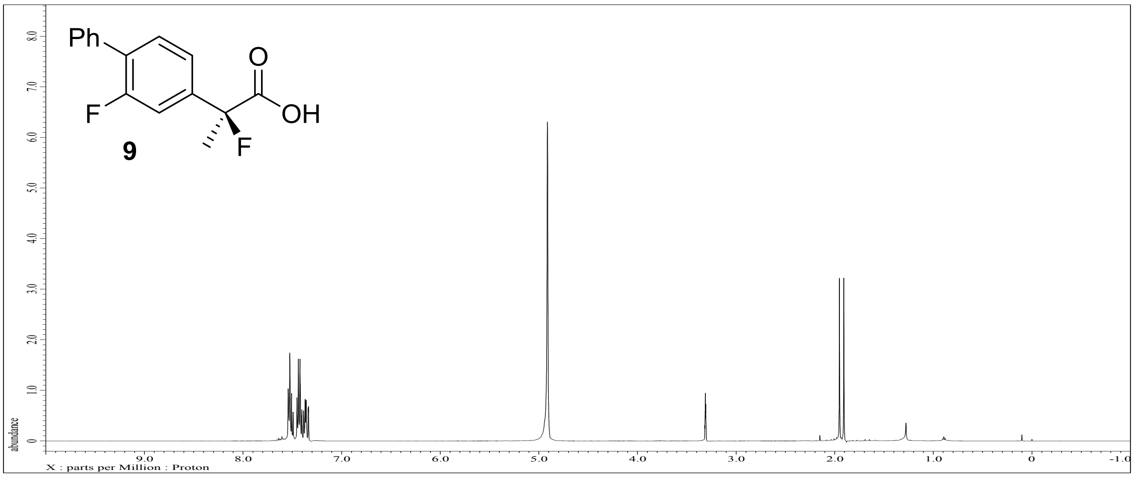

^{13}C NMR spectrum (**9**)

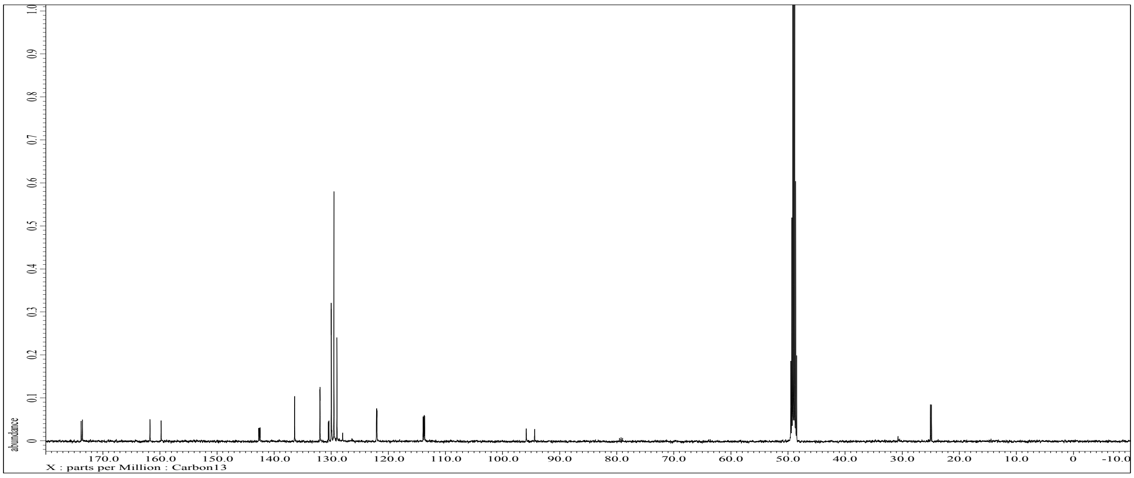

^{19}F NMR spectrum (**9**)

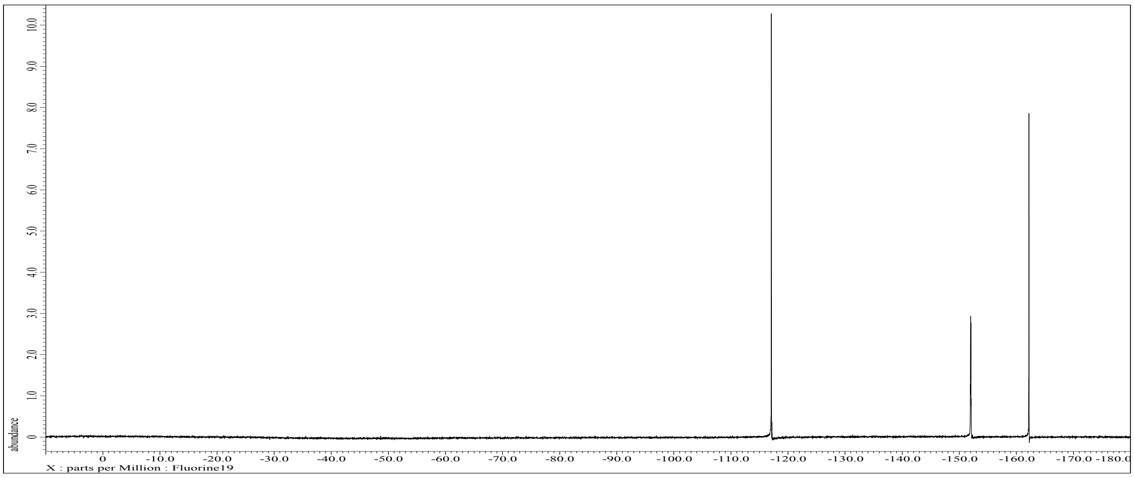

^{1}H NMR spectrum [(*R*)-**10a**]

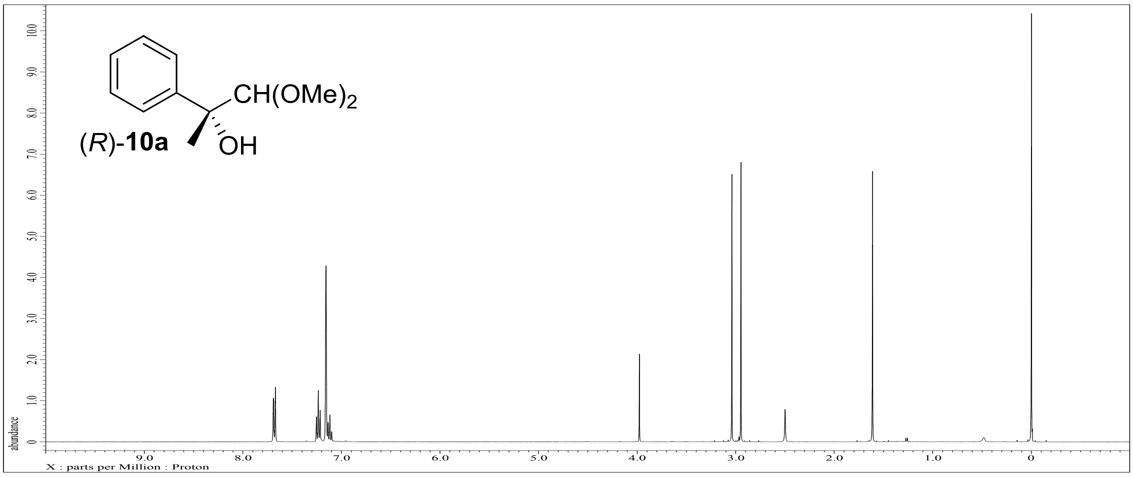

^{13}C NMR spectrum [(*R*)-**10a**]

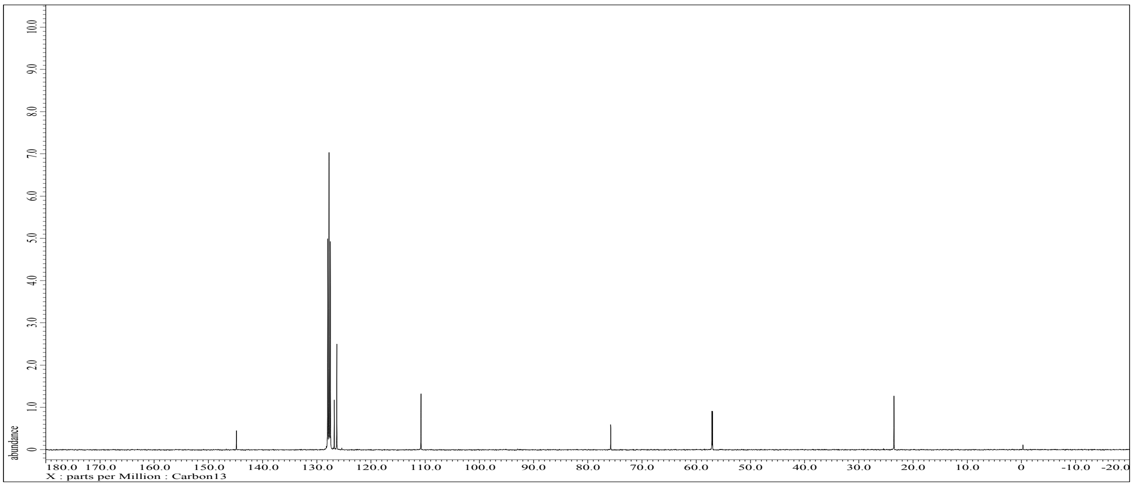

^{1}H NMR spectrum [(*R*)-**19a**]

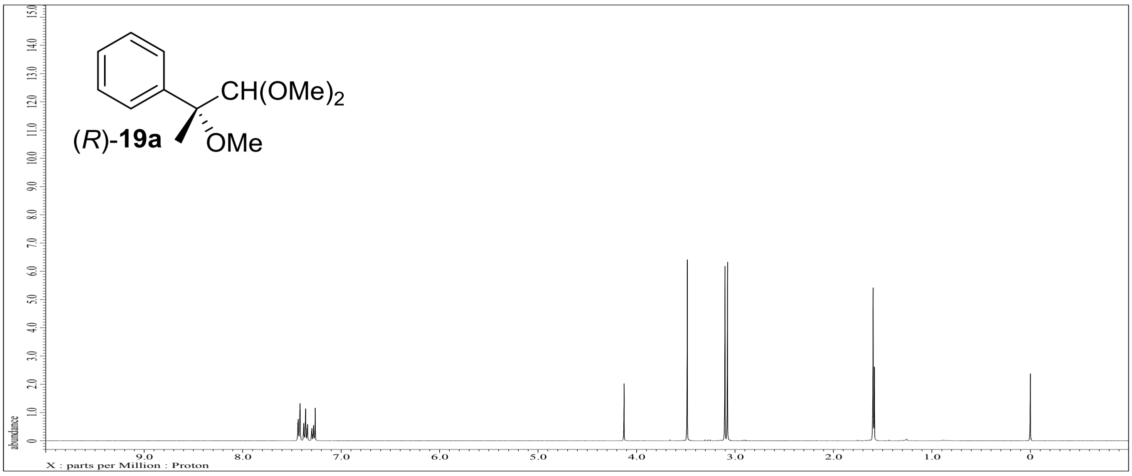

^{13}C NMR spectrum [(*R*)-**19a**]

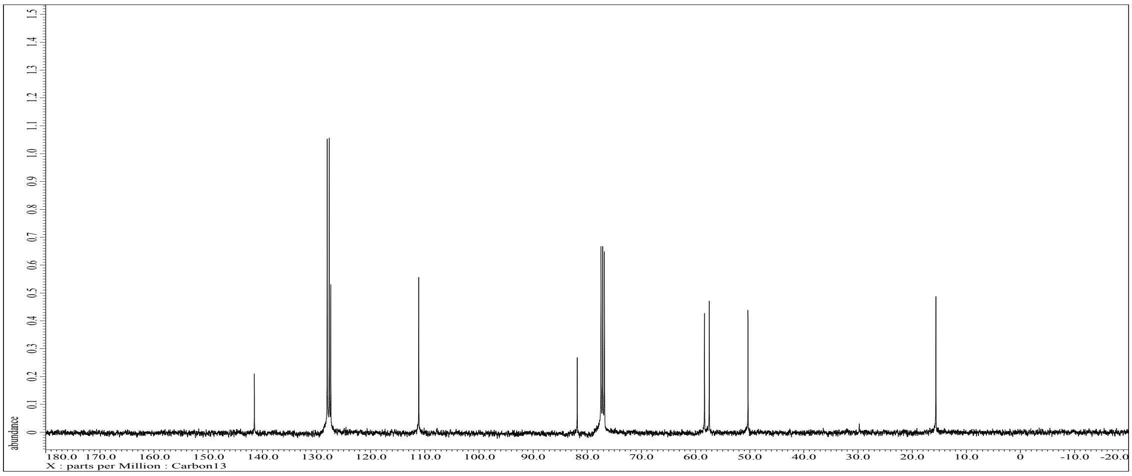

HPLC *optically active* [(*R*)-**19a**]

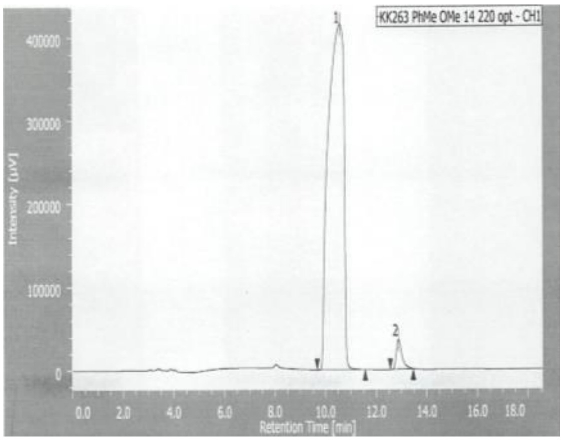

#	ピーク名	CH	tR [min]	面積 [μV·sec]	高さ [μV]	面積%	高さ%	定量値	NTP	分離度	シンメトリー係数	警告
1	Unknown	1	10.575	17602370	414230	96.971	92.058	N/A	1067	2.768	0.687	
2	Unknown	1	12.883	549750	35738	3.029	7.942	N/A	18672	N/A	1.659	

HPLC *racemic* (**19a**)

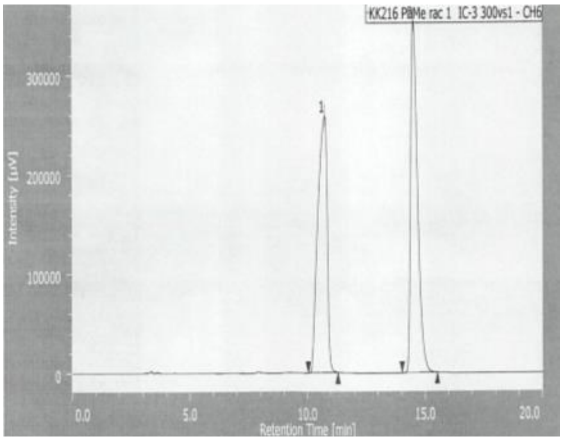

#	ピーク名	CH	tR [min]	面積 [μV·sec]	高さ [μV]	面積%	高さ%	定量値	NTP	分離度	シンメトリー係数	警告
1	Unknown	6	10.738	7071403	258609	49.363	42.172	N/A	3004	5.761	0.724	
2	Unknown	6	14.507	7253857	354614	50.637	57.828	N/A	12077	N/A	1.625	

^{1}H NMR spectrum (**10b**)

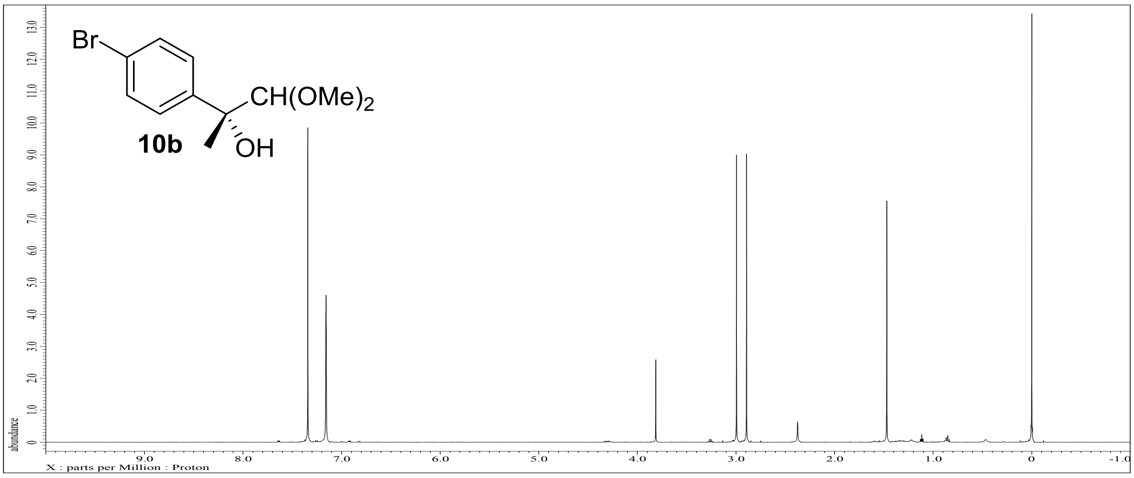

^{13}C NMR spectrum (**10b**)

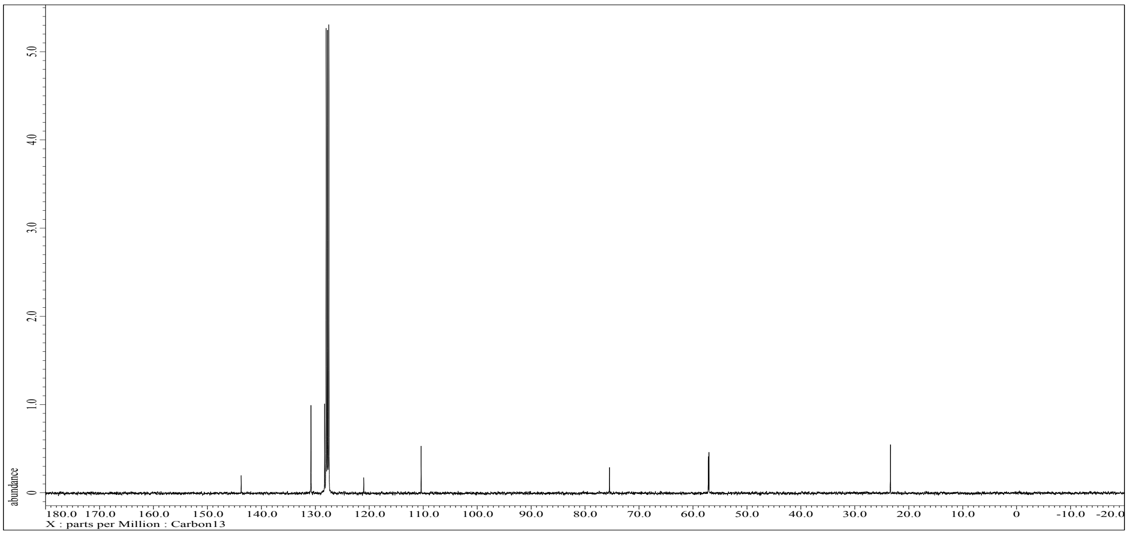

^{1}H NMR spectrum (**19b**)

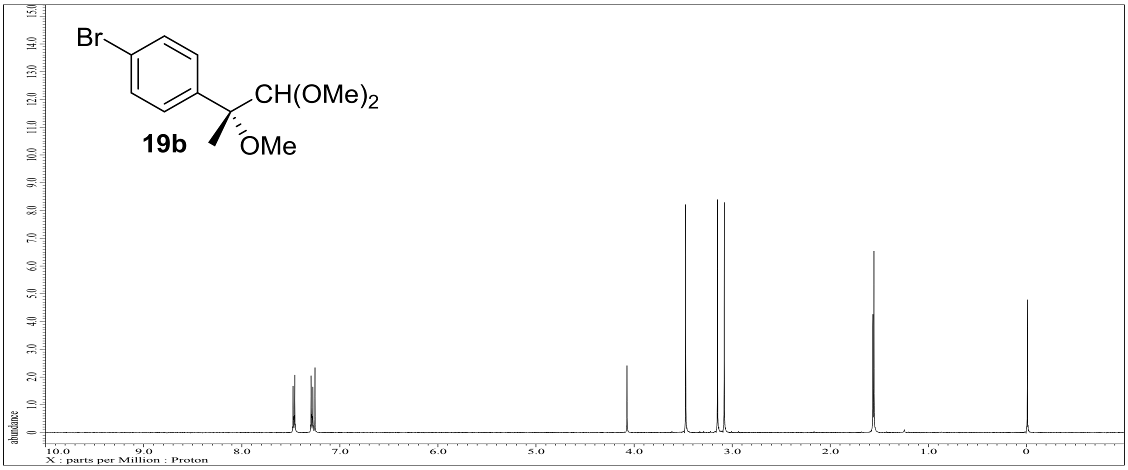

^{13}C NMR spectrum (**19b**)

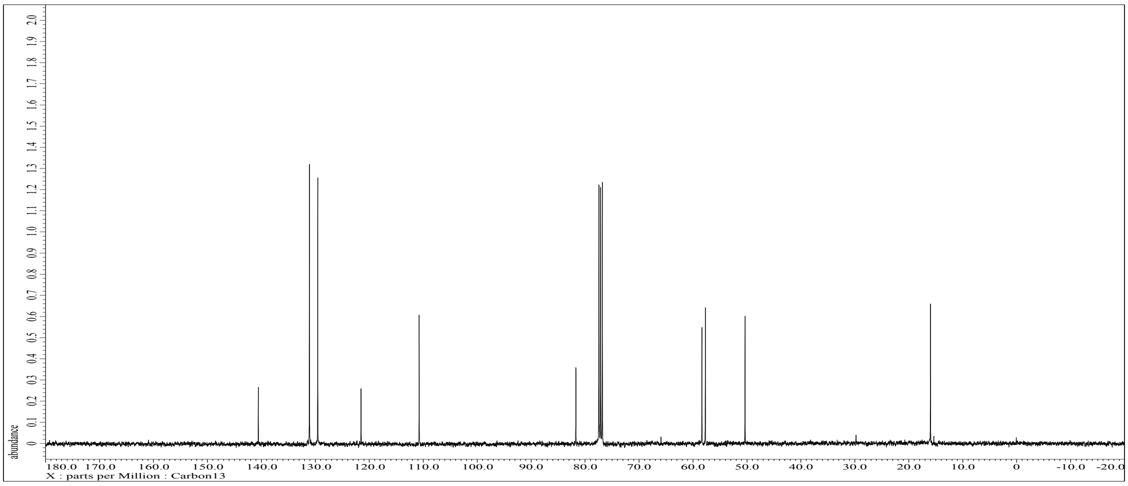

HPLC *optically active* (**19b**)

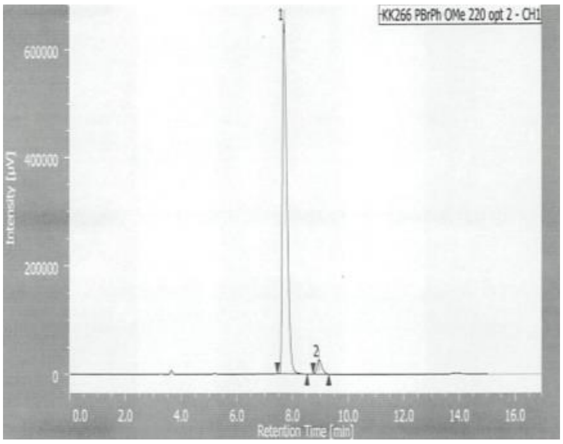

#	ピーク名	CH	tR [min]	面積 [μV·sec]	高さ [μV]	面積%	高さ%	定量値	NTP	分離度	シンメトリー係数	警告
1	Unknown	1	7.692	7339801	649186	95.921	96.127	N/A	10820	4.182	1.765	
2	Unknown	1	8.958	312154	26156	4.079	3.873	N/A	13219	N/A	1.319	

HPLC *racemic* (**19b**)

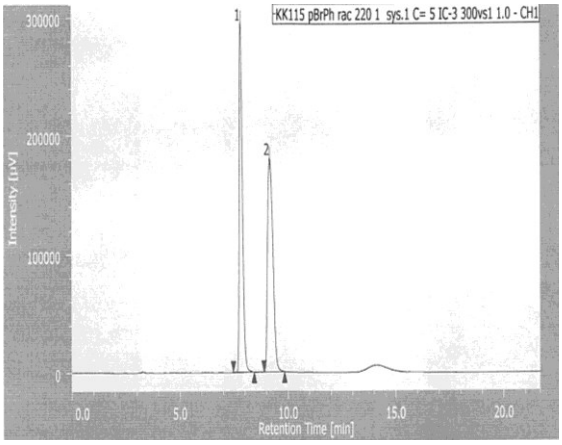

#	ピーク名	CH	tR [min]	面積 [μV·sec]	高さ [μV]	面積%	高さ%	定量値	NTP	分離度	シンメトリー係数	警告
1	Unknown	1	7.808	3006776	295724	49.078	62.222	N/A	14180	3.639	1.483	
2	Unknown	1	9.158	3119805	179551	50.922	37.778	N/A	5785	N/A	1.386	

^{1}H NMR spectrum (**10c**)

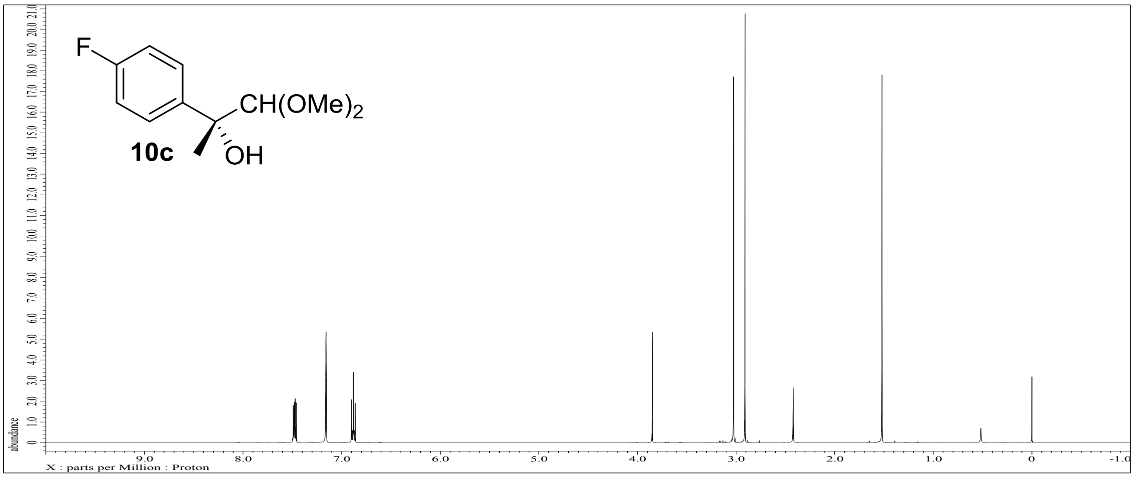

^{13}C NMR spectrum (**10c**)

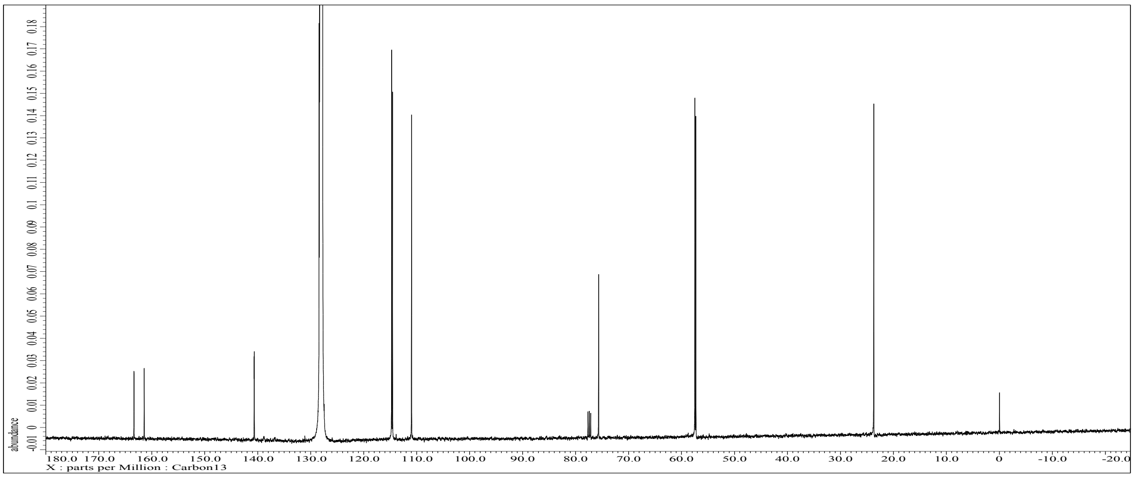

^{19}F NMR spectrum (**10c**)

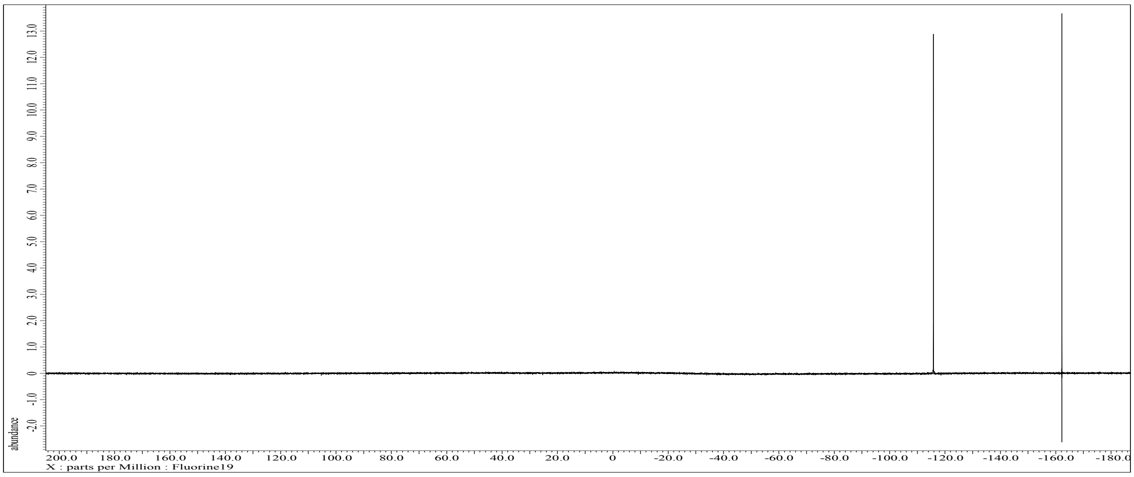

HPLC *optically active* (**10c**)

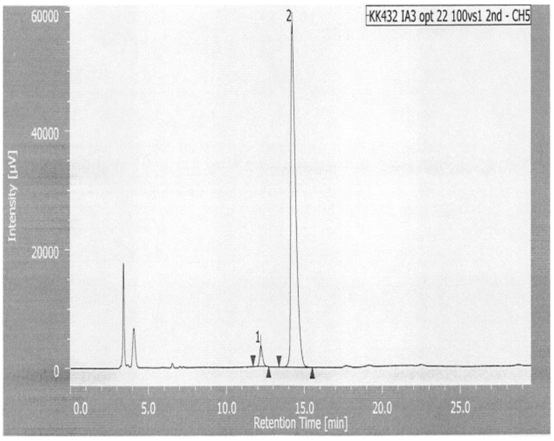

#	ピーク名	CH	tR [min]	面積 [µV·sec]	高さ [µV]	面積%	高さ%	定量値	NTP	分離度	シンメトリー係数	警告
1	Unknown	5	12.203	46374	3602	3.595	5.853	N/A	23119	4.620	1.310	
2	Unknown	5	14.205	1243498	57939	96.405	94.147	N/A	10756	N/A	2.359	

HPLC *racemic* (**10c**)

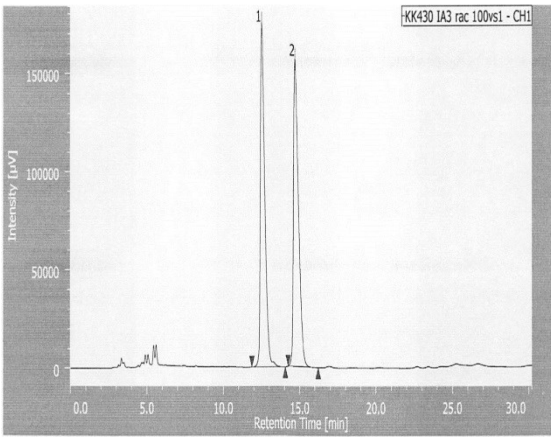

#	ピーク名	CH	tR [min]	面積 [µV·sec]	高さ [µV]	面積%	高さ%	定量値	NTP	分離度	シンメトリー係数	警告
1	Unknown	1	12.500	3511165	174524	49.482	52.769	N/A	9392	3.914	2.069	
2	Unknown	1	14.675	3584666	156207	50.518	47.231	N/A	9624	N/A	2.058	

^{1}H NMR spectrum (**10d**)

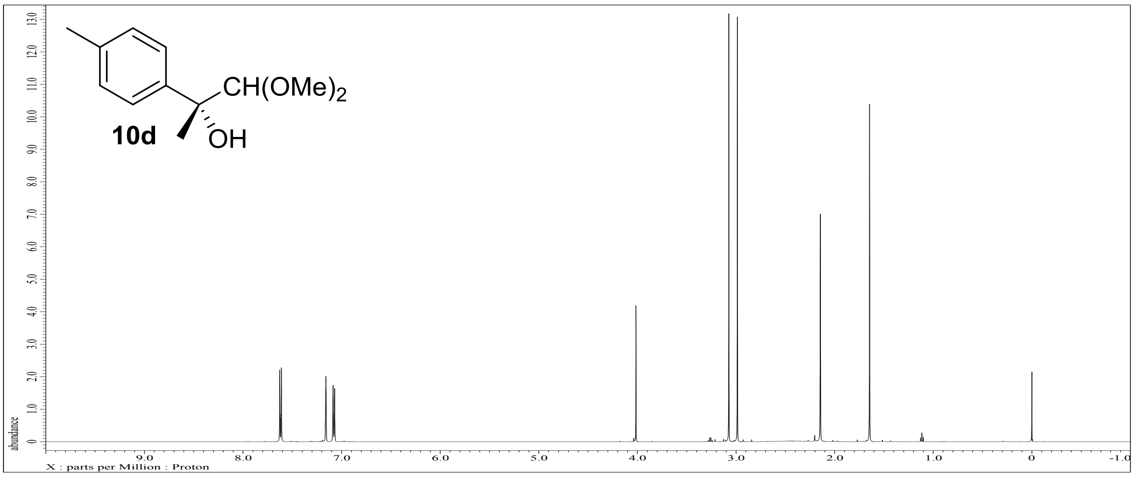

^{13}C NMR spectrum (**10d**)

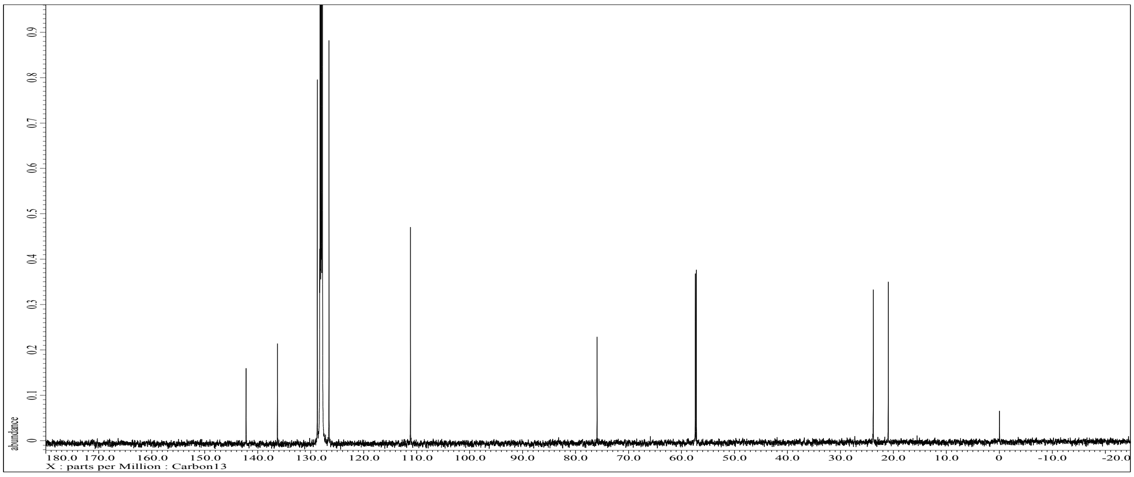

HPLC *optically active* (**10d**)

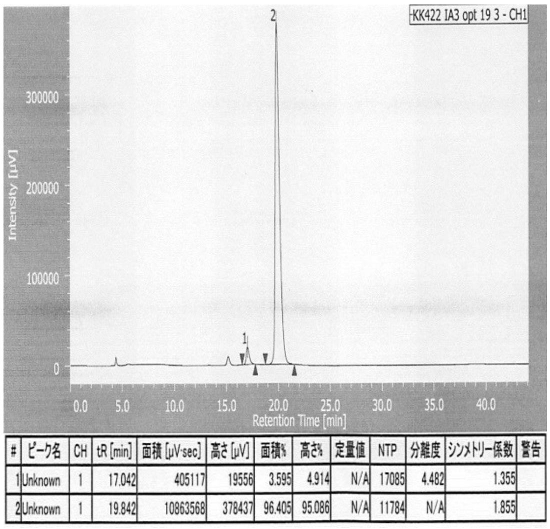

#	ピーク名	CH	tR [min]	面積 [μV·sec]	高さ [μV]	面積%	高さ%	定量値	NTP	分離度	シンメトリー係数	警告
1	Unknown	1	17.042	405117	19556	3.595	4.914	N/A	17085	4.482	1.355	
2	Unknown	1	19.842	10863568	378437	96.405	95.086	N/A	11784	N/A	1.855	

HPLC *racemic* (**10d**)

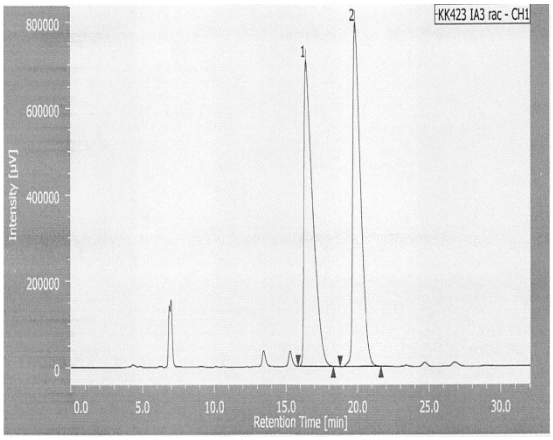

#	ピーク名	CH	tR [min]	面積 [μV·sec]	高さ [μV]	面積%	高さ%	定量値	NTP	分離度	シンメトリー係数	警告
1	Unknown	1	16.400	30215188	707099	50.168	47.058	N/A	3480	3.258	2.989	
2	Unknown	1	19.808	30013068	795500	49.832	52.942	N/A	6465	N/A	2.244	

^{1}H NMR spectrum (**10h**)

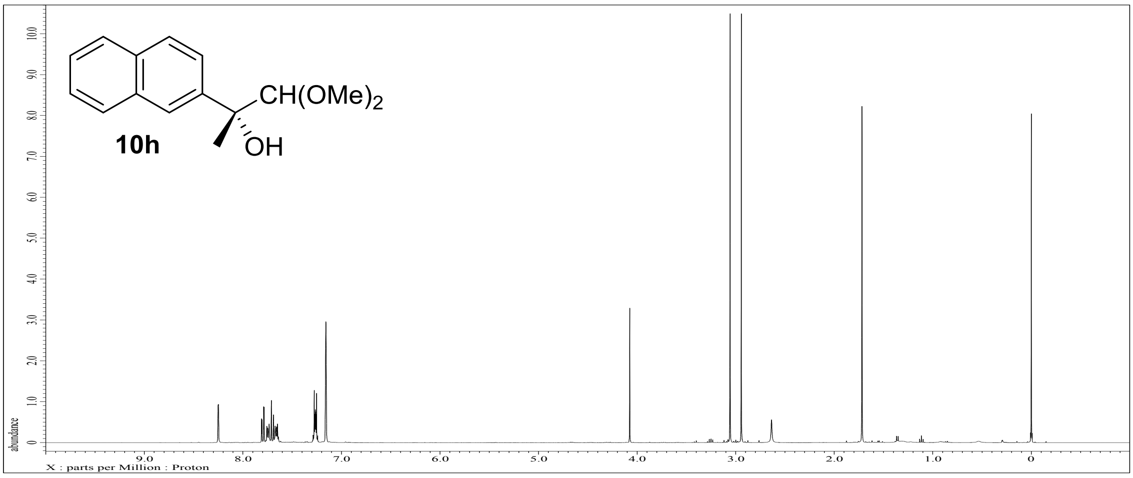

^{13}C NMR spectrum (**10h**)

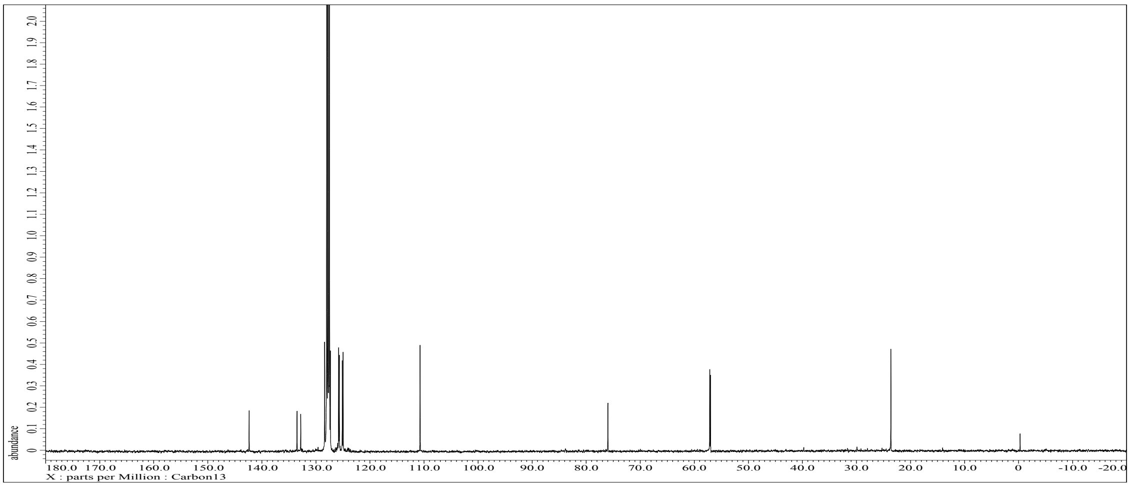

^{1}H NMR spectrum (**19h**)

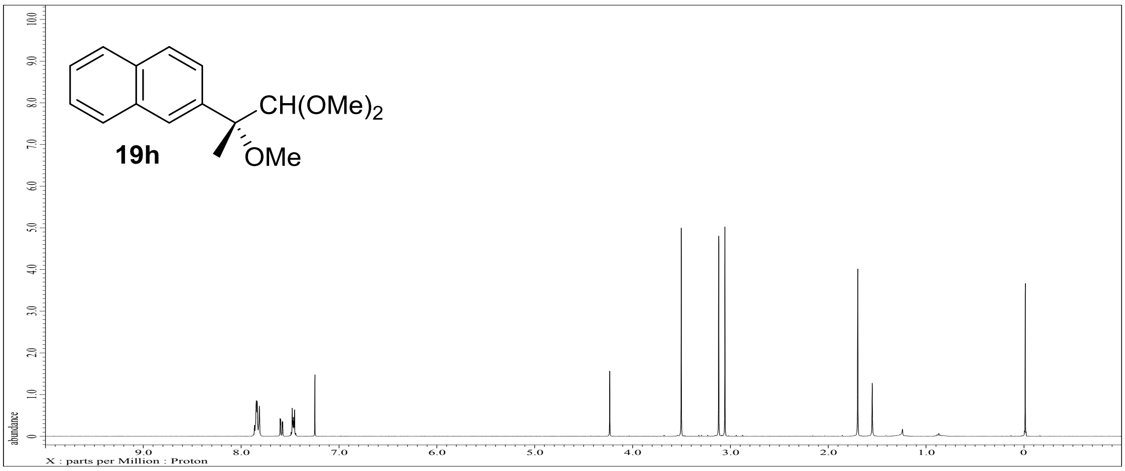

^{13}C NMR spectrum (**19h**)

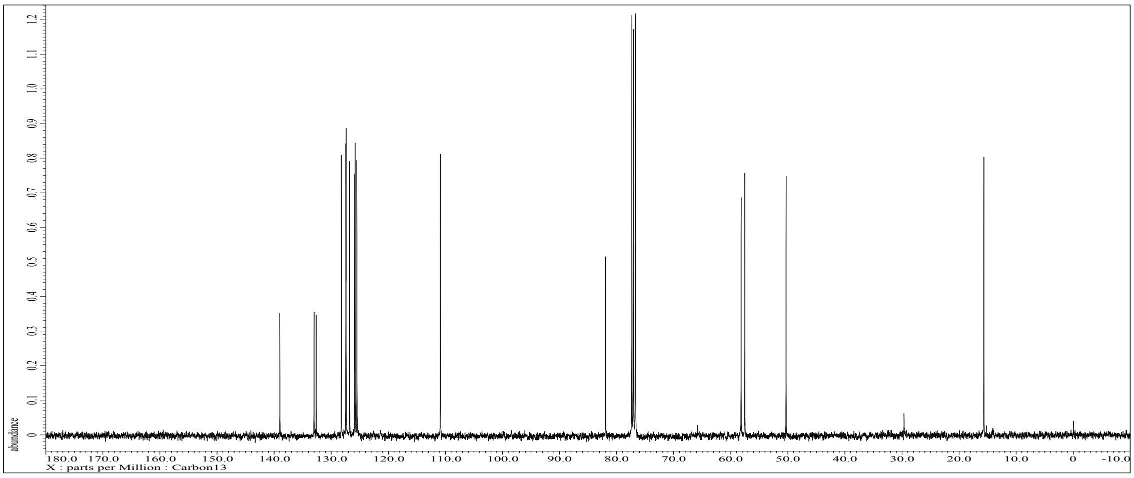

HPLC *optically active* (**19h**)

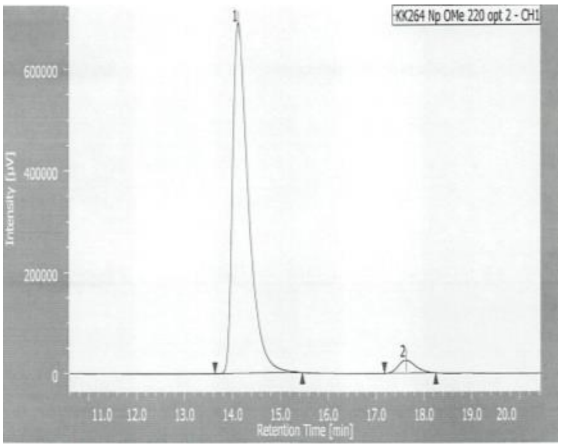

#	ピーク名	CH	tR [min]	面積 [µV·sec]	高さ [µV]	面積%	高さ%	定量値	NTP	分離度	シンメトリー係数	警告
1	Unknown	1	14.117	16020688	690588	96.158	96.456	N/A	9303	5.628	1.814	
2	Unknown	1	17.625	640099	25376	3.842	3.544	N/A	11254	N/A	1.244	

HPLC *racemic* (**19h**)

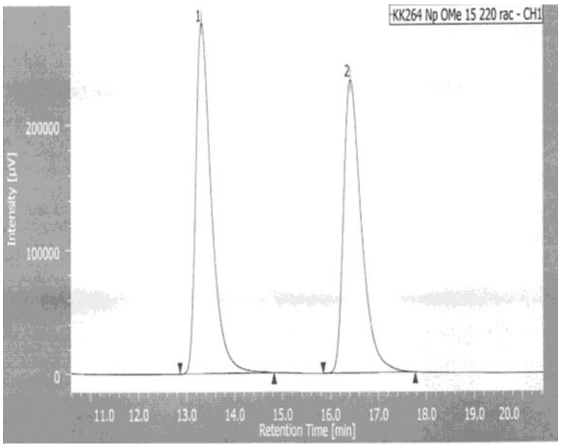

#	ピーク名	CH	tR [min]	面積 [µV·sec]	高さ [µV]	面積%	高さ%	定量値	NTP	分離度	シンメトリー係数	警告
1	Unknown	1	13.317	6388197	282002	50.932	54.511	N/A	8945	5.086	1.653	
2	Unknown	1	16.417	6154392	235333	49.068	45.489	N/A	9929	N/A	1.576	

^{1}H NMR spectrum (**10i**)

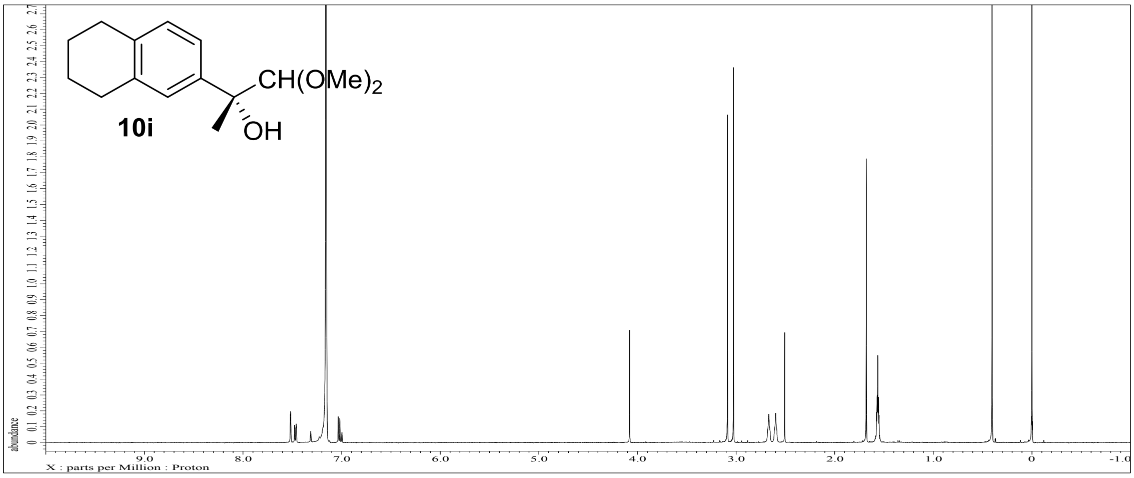

^{13}C NMR spectrum (**10i**)

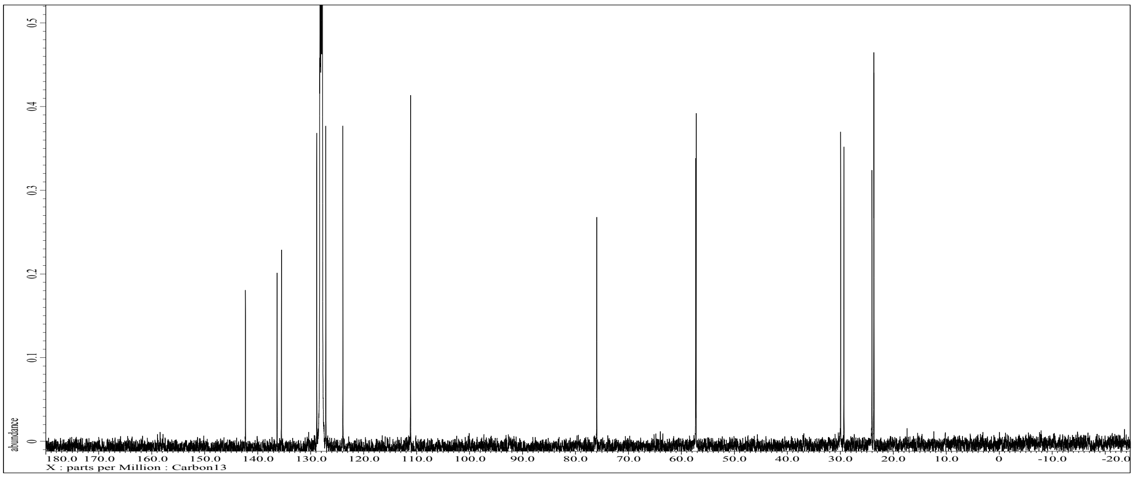

HPLC *optically active* (**10i**)

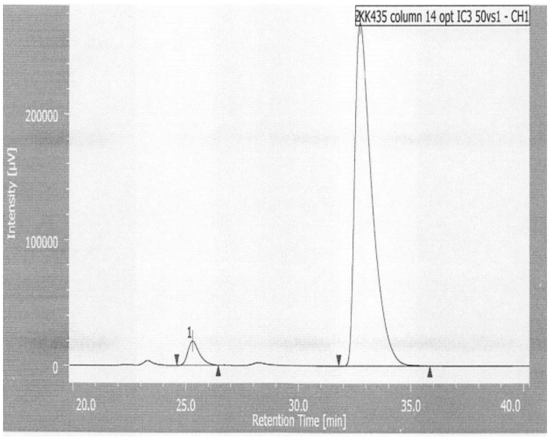

#	ピーク名	CH	tR [min]	面積 [μV·sec]	高さ [μV]	面積%	高さ%	定量値	NTP	分離度	シンメトリー係数	警告
1	Unknown	1	25.300	677122	19488	4.436	6.670	N/A	13377	6.650	1.413	
2	Unknown	1	32.733	14587405	272699	95.564	93.330	N/A	9180	N/A	2.030	

HPLC *racemic* (**10i**)

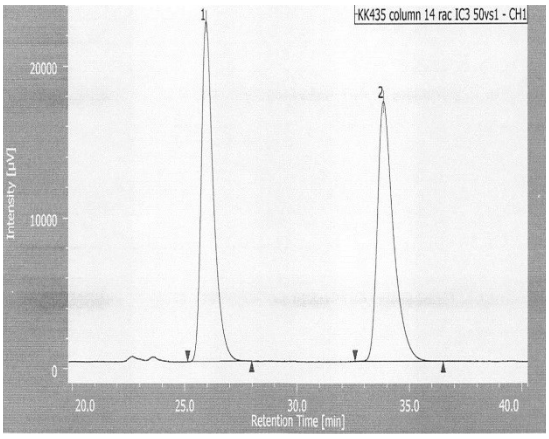

#	ピーク名	CH	tR [min]	面積 [μV·sec]	高さ [μV]	面積%	高さ%	定量値	NTP	分離度	シンメトリー係数	警告
1	Unknown	1	25.950	841184	22451	49.936	56.666	N/A	12332	7.328	1.493	
2	Unknown	1	33.850	843352	17168	50.064	43.334	N/A	12175	N/A	1.535	

^{1}H NMR spectrum (**10j**)

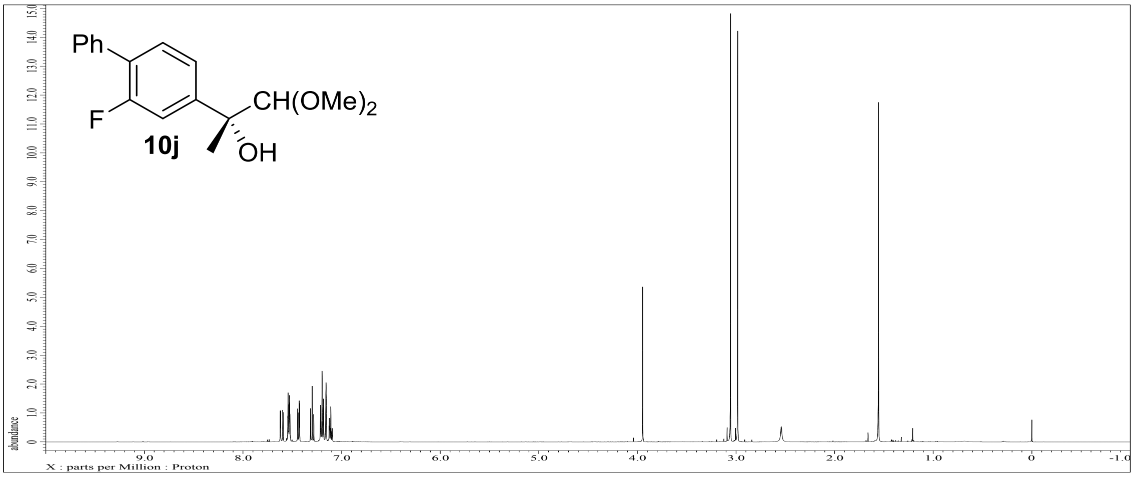

^{13}C NMR spectrum (**10j**)

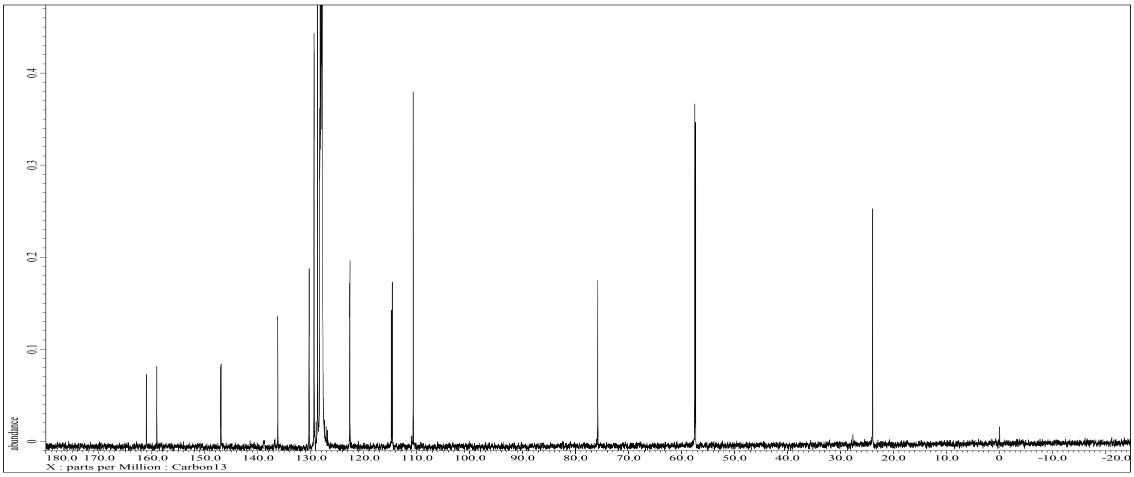

^{19}F NMR spectrum (**10j**)

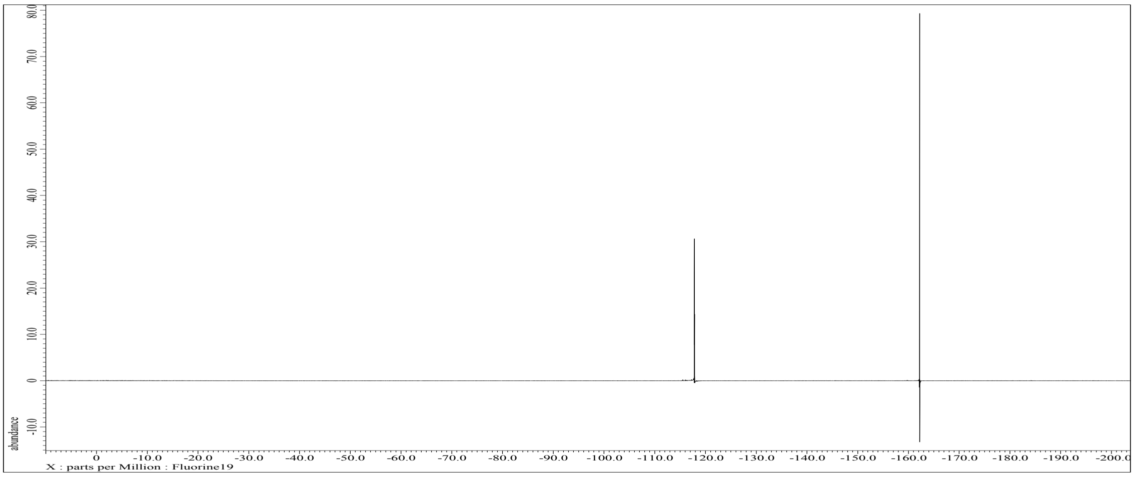

HPLC *optically active* (**10j**)

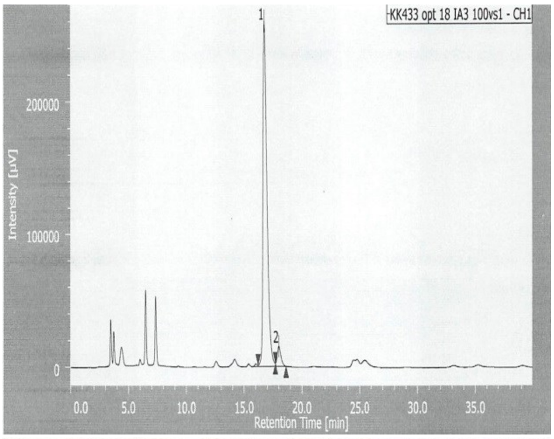

#	ピーク名	CH	tR [min]	面積 [µV·sec]	高さ [µV]	面積%	高さ%	定量値	NTP	分離度	シンメトリー係数	警告
1	Unknown	1	16.733	6223020	259333	95.006	94.518	N/A	12400	2.190	1.870	
2	Unknown	1	17.992	327125	15040	4.994	5.482	N/A	17053	N/A	N/A	

HPLC *racemic* (**10j**)

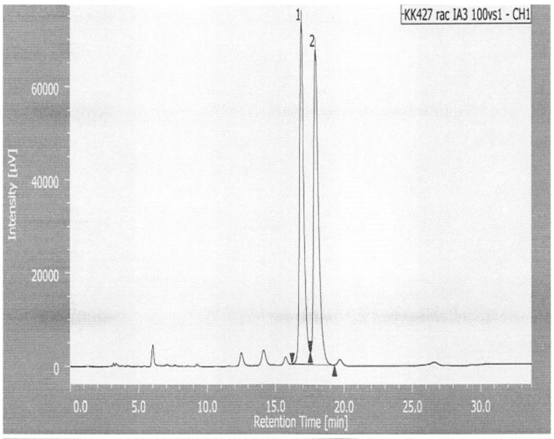

#	ピーク名	CH	tR [min]	面積 [µV·sec]	高さ [µV]	面積%	高さ%	定量値	NTP	分離度	シンメトリー係数	警告
1	Unknown	1	16.875	1639611	73291	49.896	52.123	N/A	14780	1.783	1.551	
2	Unknown	1	17.908	1646449	67321	50.104	47.877	N/A	13933	N/A	1.648	

^{1}H NMR spectrum (**10k**)

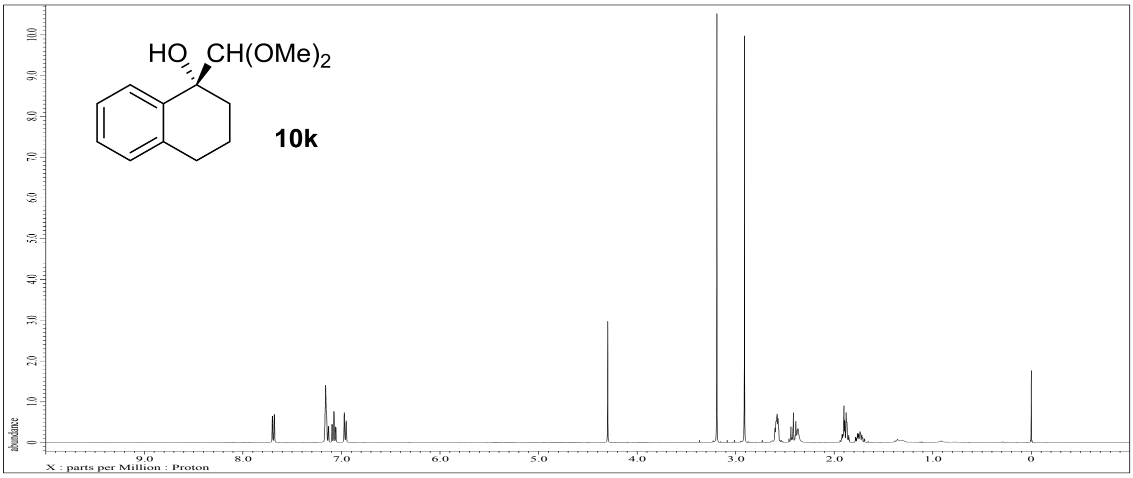

^{13}C NMR spectrum (**10k**)

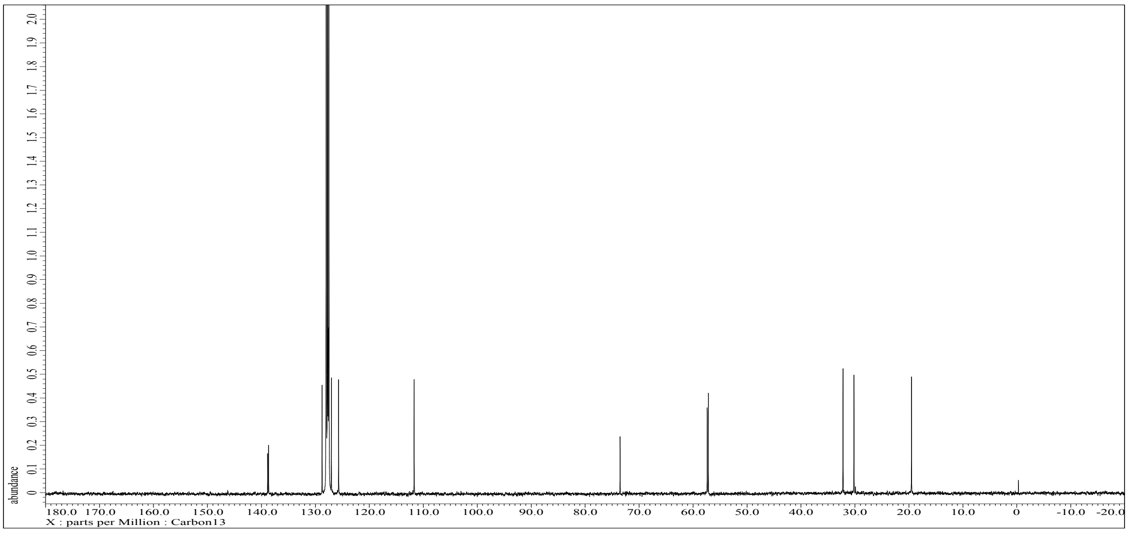

HPLC *optically active* (**10k**)

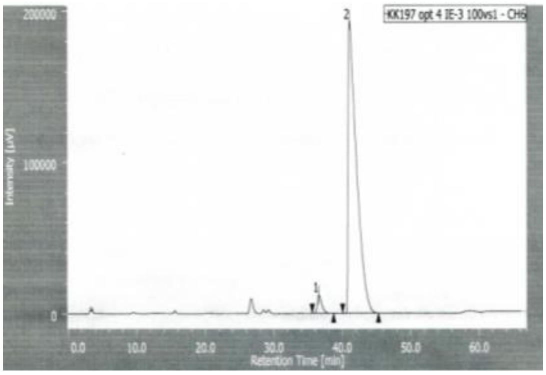

#	ピーク名	CH	tR [min]	面積 [µV·sec]	高さ [µV]	面積%	高さ%	定量値	NTP	分離度	シンメトリー係数	警告
1	Unknown	6	36.592	540064	12126	3.187	5.891	N/A	16736	2.747	1.613	
2	Unknown	6	41.132	16407744	193723	96.813	94.109	N/A	5683	N/A	3.755	

HPLC *racemic* (**10k**)

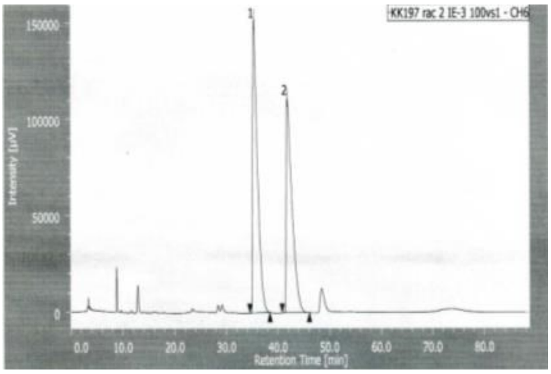

#	ピーク名	CH	tR [min]	面積 [µV·sec]	高さ [µV]	面積%	高さ%	定量値	NTP	分離度	シンメトリー係数	警告
1	Unknown	6	35.338	9623164	152485	52.220	57.728	N/A	7675	3.594	3.097	
2	Unknown	6	41.845	8804952	111657	47.780	42.272	N/A	6884	N/A	3.347	

^{1}H NMR spectrum (**10l**)

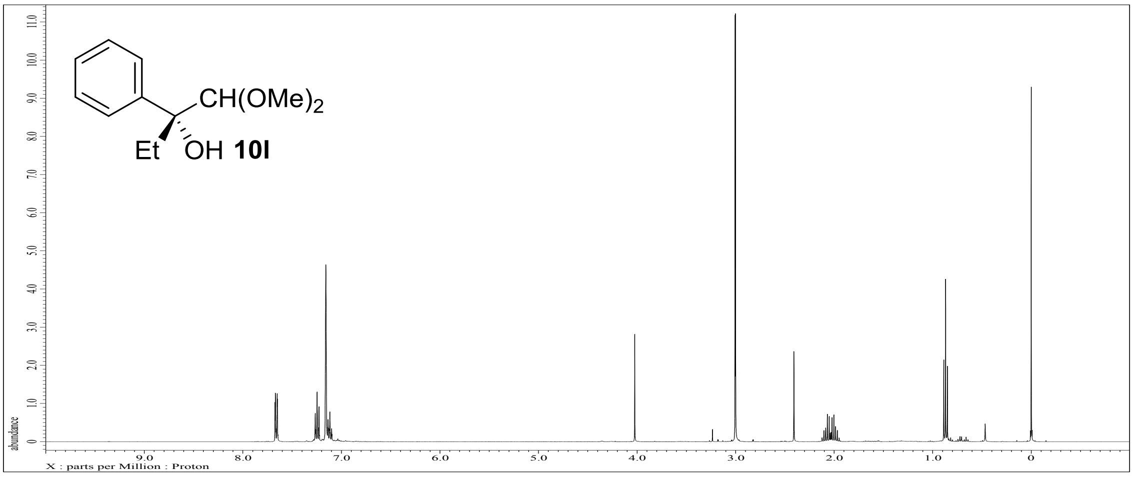

^{13}C NMR spectrum (**10l**)

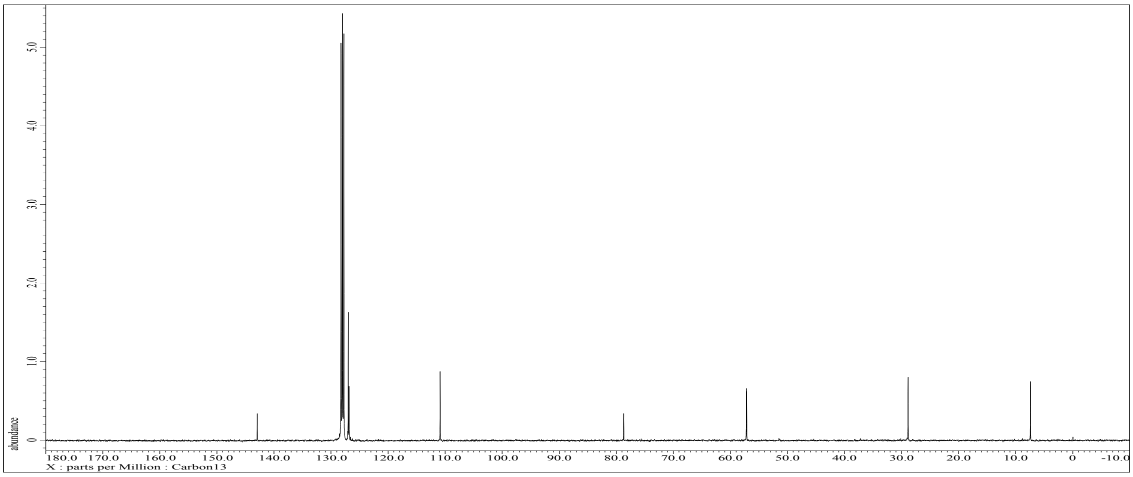

HPLC *optically active* (**10l**)

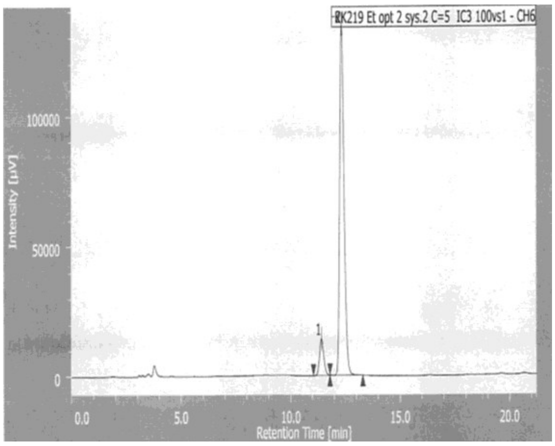

HPLC *racemic* (**10l**)

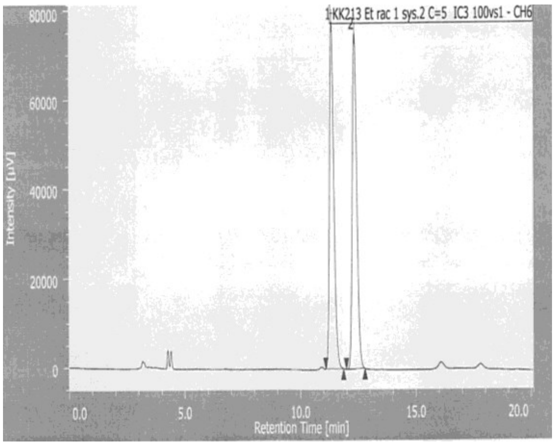

#	ピーク名	CH	tR [min]	面積 [µV·sec]	高さ [µV]	面積%	高さ%	定量値	NTP	分離度	シンメトリー係数	警告
1	Unknown	6	11.405	191326	14358	9.116	9.630	N/A	17519	2.615	1.190	
2	Unknown	6	12.335	1907501	134732	90.884	90.370	N/A	17920	N/A	1.261	

#	ピーク名	CH	tR [min]	面積 [µV·sec]	高さ [µV]	面積%	高さ%	定量値	NTP	分離度	シンメトリー係数	警告
1	Unknown	6	11.355	1026054	77875	50.285	50.857	N/A	17660	2.865	1.295	
2	Unknown	6	12.348	1014421	75251	49.715	49.143	N/A	19529	N/A	1.193	

^{1}H NMR spectrum (**11**)

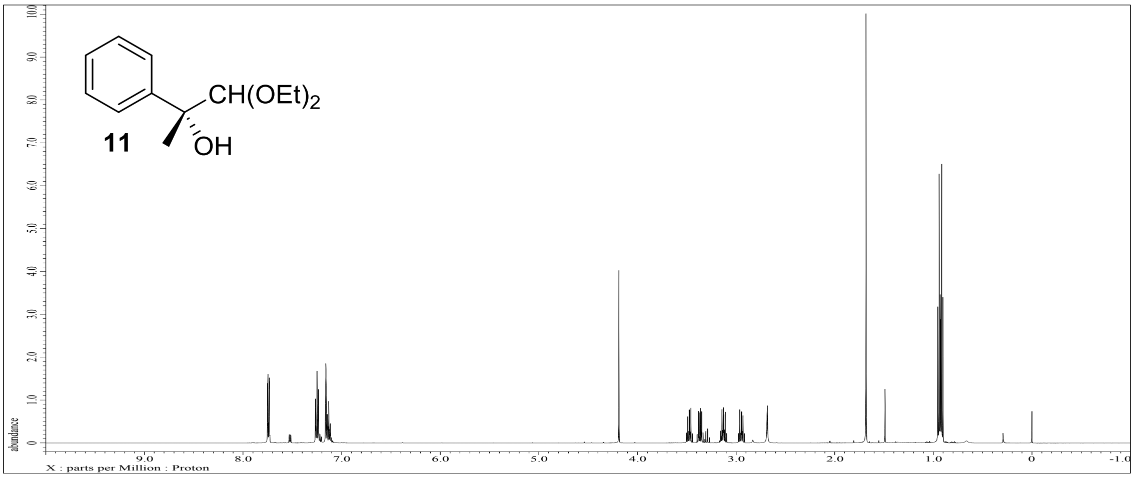

^{13}C NMR spectrum (**11**)

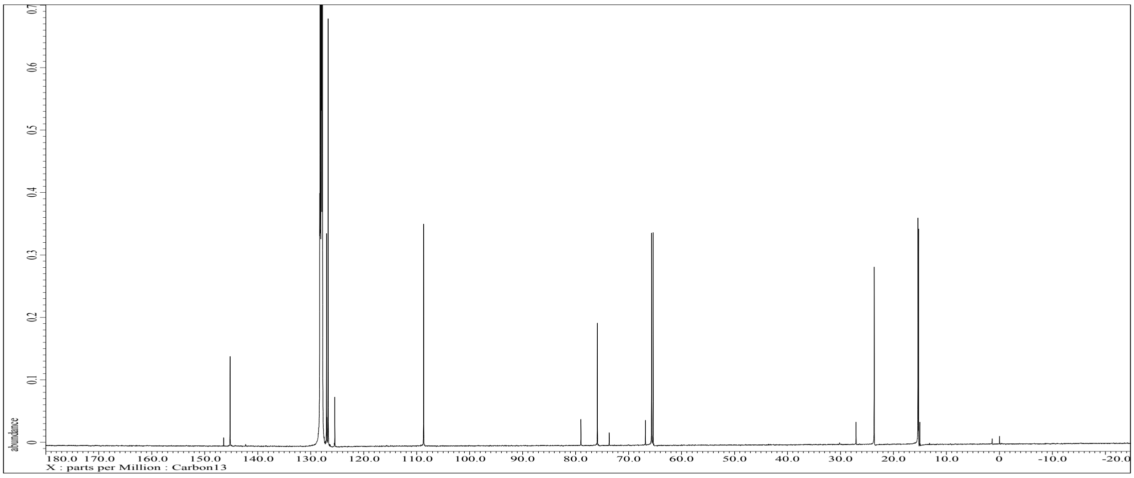

HPLC *optically active* (**11**)

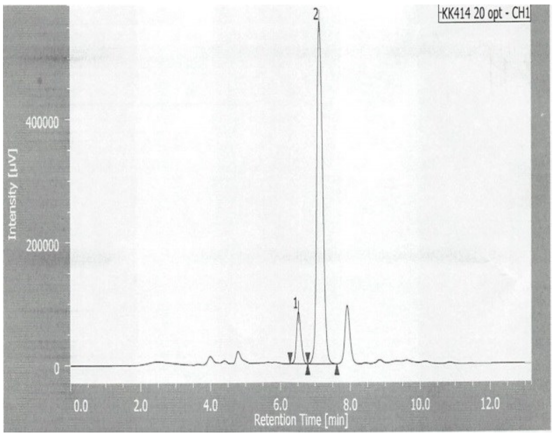

#	ピーク名	CH	tR [min]	面積 [μV·sec]	高さ [μV]	面積%	高さ%	定量値	NTP	分離度	シンメトリー係数	警告
1	Unknown	1	6.517	752349	84592	10.304	13.174	N/A	12574	2.238	1.156	
2	Unknown	1	7.125	6549056	557517	89.696	86.826	N/A	8305	N/A	1.174	

HPLC *racemic* (**11**)

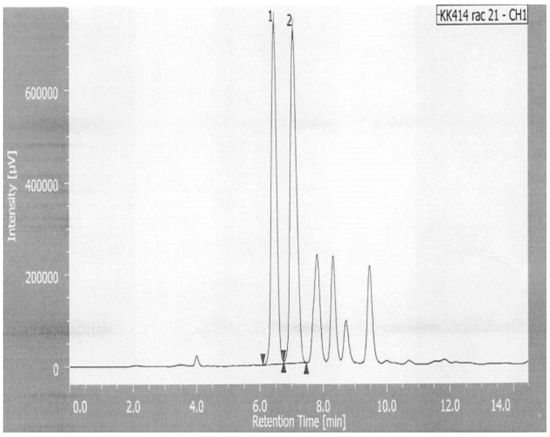

#	ピーク名	CH	tR [min]	面積 [μV·sec]	高さ [μV]	面積%	高さ%	定量値	NTP	分離度	シンメトリー係数	警告
1	Unknown	1	6.442	8685911	739751	46.223	50.540	N/A	6637	1.719	1.130	
2	Unknown	1	7.033	10105591	723936	53.777	49.460	N/A	5665	N/A	1.356	

^{1}H NMR spectrum (**12a**)

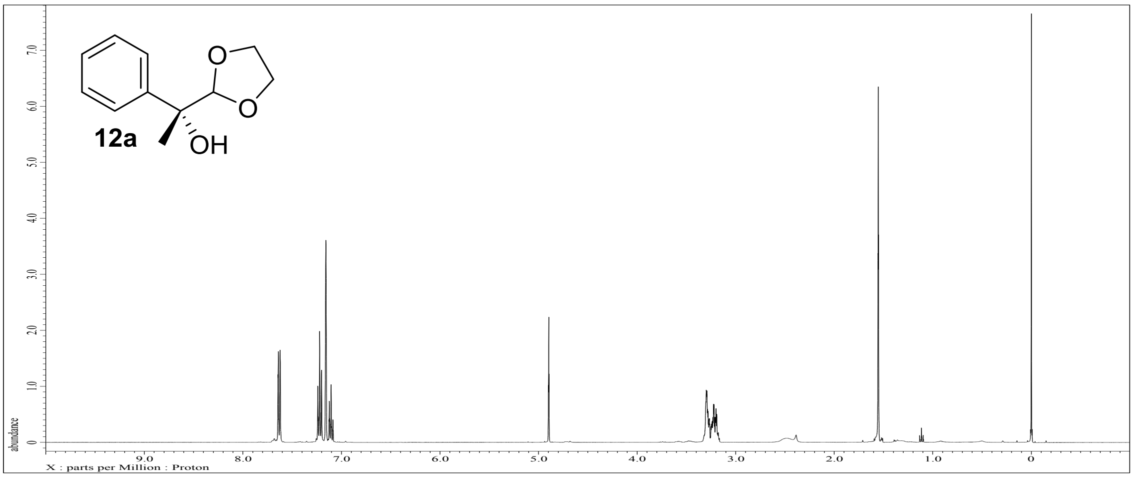

^{13}C NMR spectrum (**12a**)

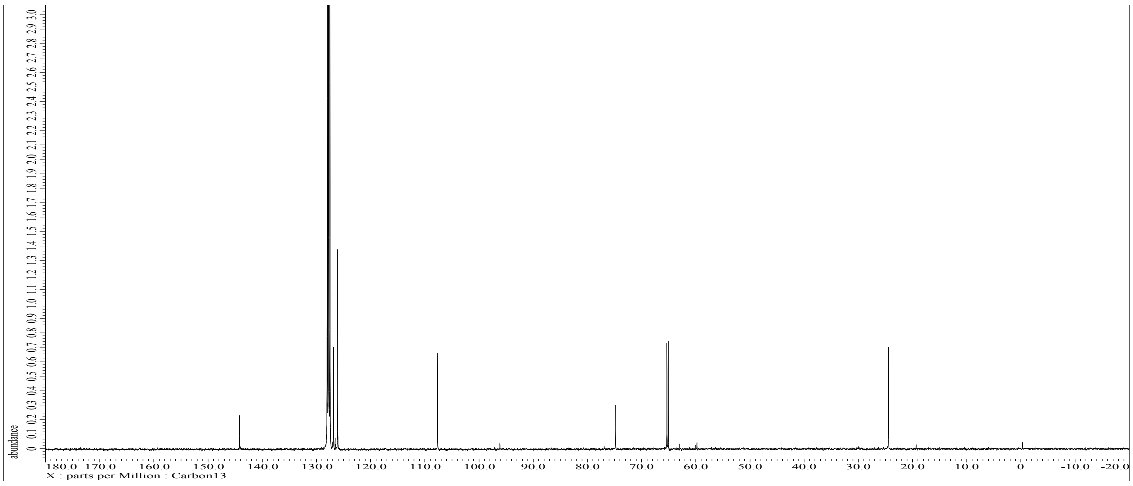

HPLC *optically active* (**12a**)

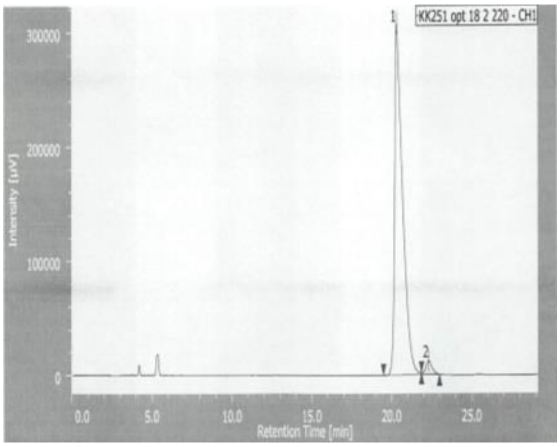

#	ピーク名	CH	tR [min]	面積 [µV·sec]	高さ [µV]	面積%	高さ%	定量値	NTP	分離度	シンメトリー係数	警告
1	Unknown	1	20.308	10567621	307717	96.905	96.151	N/A	9006	2.605	2.121	
2	Unknown	1	22.300	337564	12318	3.095	3.849	N/A	17352	N/A	N/A	

HPLC *racemic* (**12a**)

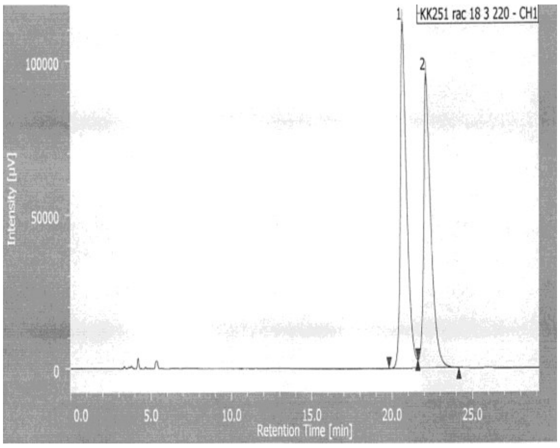

#	ピーク名	CH	tR [min]	面積 [µV·sec]	高さ [µV]	面積%	高さ%	定量値	NTP	分離度	シンメトリー係数	警告
1	Unknown	1	20.642	3128643	112697	50.834	54.073	N/A	14938	2.049	1.683	
2	Unknown	1	22.100	3025978	95721	49.166	45.927	N/A	13818	N/A	1.717	

^{1}H NMR spectrum (**12j**)

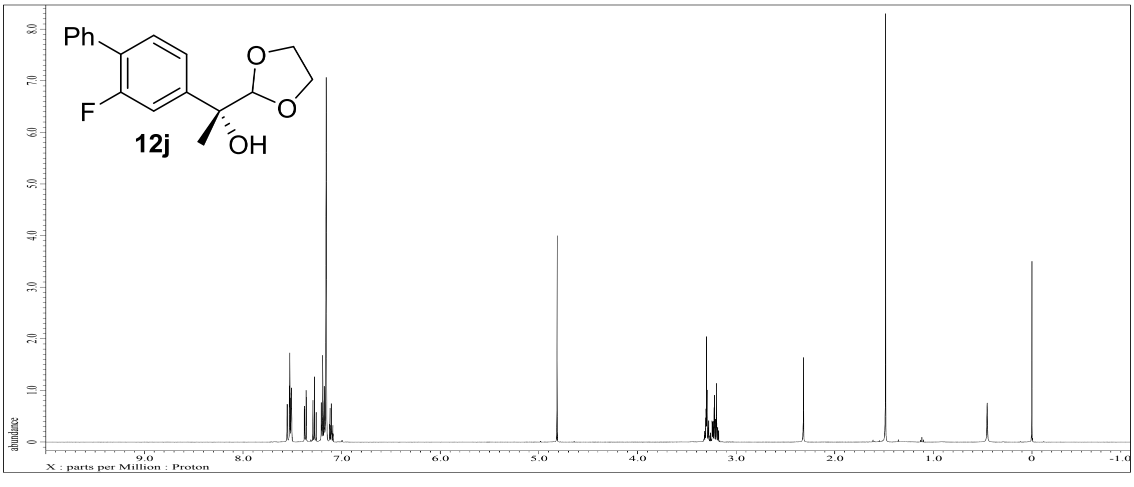

^{13}C NMR spectrum (**12j**)

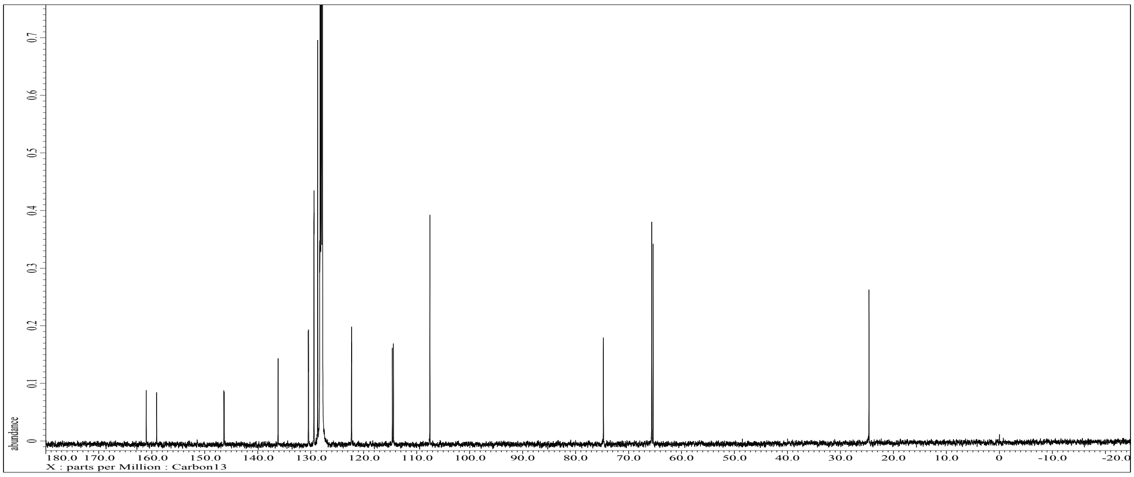

^{19}F NMR spectrum (**12j**)

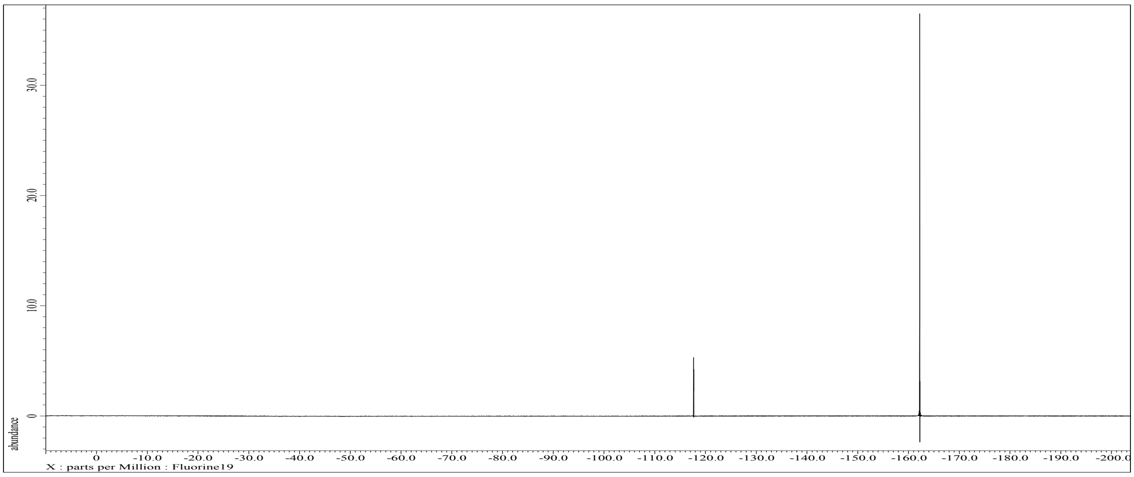

HPLC *optically active* (**12j**)

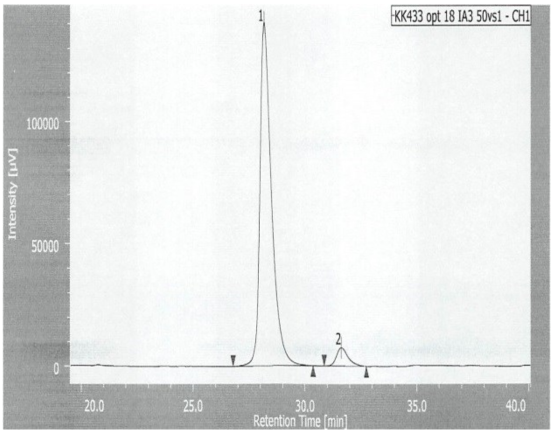

#	ピーク名	CH	tR [min]	面積 [µV·sec]	高さ [µV]	面積%	高さ%	定量値	NTP	分離度	シンメトリー係数	警告
1	Unknown	1	28.158	5033274	140355	94.881	94.839	N/A	16300	3.919	1.617	
2	Unknown	1	31.617	271559	7638	5.119	5.161	N/A	20318	N/A	1.369	

HPLC *racemic* (**12j**)

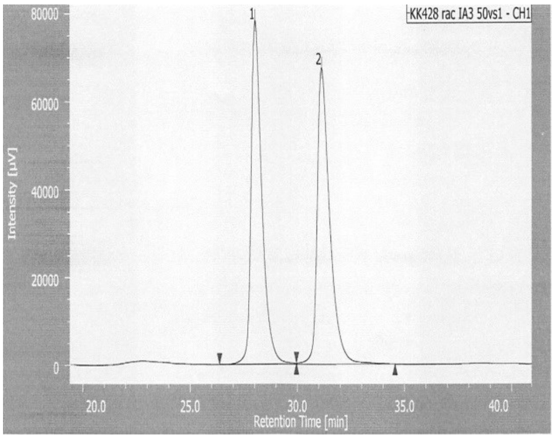

#	ピーク名	CH	tR [min]	面積 [µV·sec]	高さ [µV]	面積%	高さ%	定量値	NTP	分離度	シンメトリー係数	警告
1	Unknown	1	28.033	2711315	78195	49.695	53.693	N/A	17375	3.368	1.563	
2	Unknown	1	31.133	2744590	67439	50.305	46.307	N/A	15670	N/A	1.676	

^{1}H NMR spectrum [(*R*)-**13**]

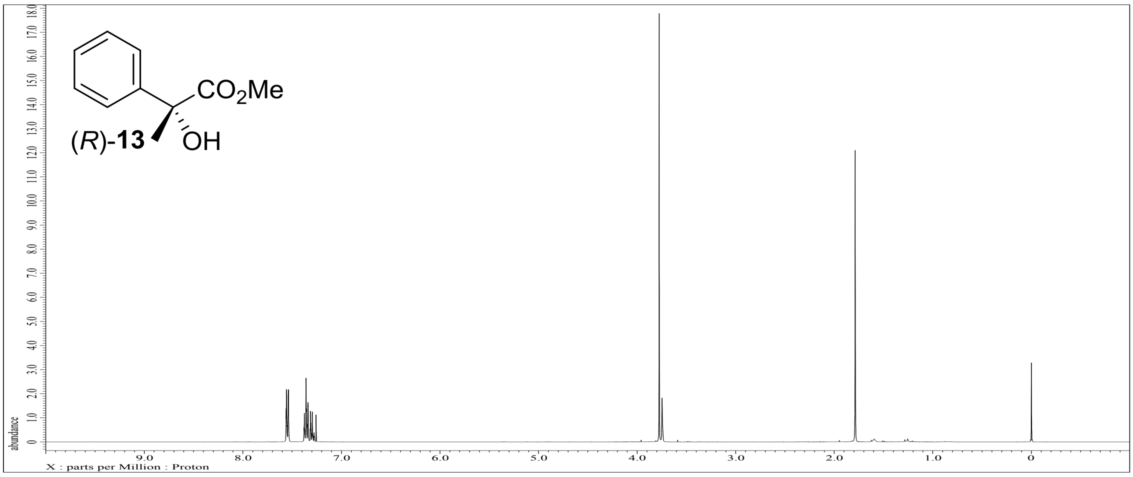

^{13}C NMR spectrum [(*R*)-**13**]

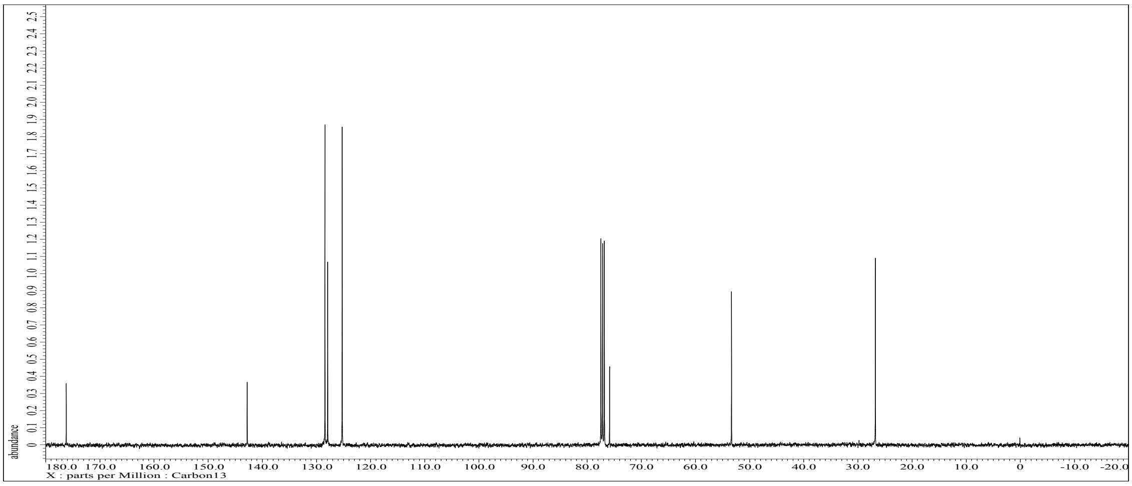

HPLC *optically active* [(*R*)-**13**]

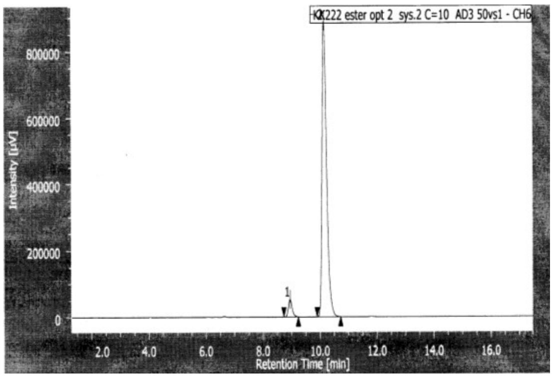

#	ピーク名	CH	tR [min]	面積 [µV·sec]	高さ [µV]	面積%	高さ%	定量値	NTP	分離度	シンメトリー係数	警告
1	Unknown	6	8.947	482599	52932	4.615	5.620	N/A	23567	4.651	1.212	
2	Unknown	6	10.150	9974732	888842	95.385	94.380	N/A	20187	N/A	1.385	

HPLC *racemic* [(*R*)-**13**]

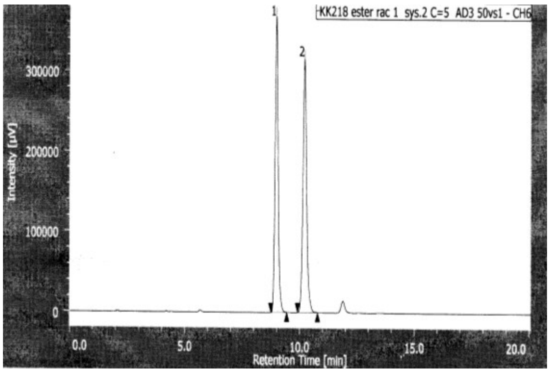

#	ピーク名	CH	tR [min]	面積 [µV·sec]	高さ [µV]	面積%	高さ%	定量値	NTP	分離度	シンメトリー係数	警告
1	Unknown	6	8.973	3452767	367434	49.284	53.836	N/A	22752	4.770	1.262	
2	Unknown	6	10.207	3553053	315066	50.716	46.164	N/A	21172	N/A	1.225	
